# The celery genome sequence reveals sequential paleo‐polyploidizations, karyotype evolution and resistance gene reduction in apiales

**DOI:** 10.1111/pbi.13499

**Published:** 2020-11-18

**Authors:** Xiaoming Song, Pengchuan Sun, Jiaqing Yuan, Ke Gong, Nan Li, Fanbo Meng, Zhikang Zhang, Xinyu Li, Jingjing Hu, Jinpeng Wang, Qihang Yang, Beibei Jiao, Fulei Nie, Tao Liu, Wei Chen, Shuyan Feng, Qiaoying Pei, Tong Yu, Xi Kang, Wei Zhao, Chunlin Cui, Ying Yu, Tong Wu, Lanxing Shan, Man Liu, Zhiji Qin, Hao Lin, Rajeev K. Varshney, Xiu‐Qing Li, Andrew H. Paterson, Xiyin Wang

**Affiliations:** ^1^ School of Life Sciences/Center for Genomics and Bio‐computing North China University of Science and Technology Tangshan Hebei China; ^2^ National Key Laboratory for North China Crop Improvement and Regulation Hebei Agriculture University Baoding Hebei China; ^3^ School of Life Science and Technology and Center for Informational Biology University of Electronic Science and Technology of China Chengdu China; ^4^ Key Laboratory of Bio‐Resource and Eco‐Environment of Ministry of Education College of Life Sciences Sichuan University Chengdu China; ^5^ College of Life Sciences Shaanxi Normal University Xi'an China; ^6^ Center of Excellence in Genomics & Systems Biology International Crops Research Institute for the Semi‐Arid Tropics (ICRISAT) Patancheru India; ^7^ Fredericton Research and Development Centre Agriculture and Agri‐Food Canada Fredericton New Brunswick Canada; ^8^ Plant Genome Mapping Laboratory University of Georgia Athens GA USA

**Keywords:** celery genome, paleo‐polyploidizations, karyotype reconstruction, resistance gene, coumarins

## Abstract

Celery (*Apium graveolens* L. 2*n* = 2*x* = 22), a member of the Apiaceae family, is among the most important and globally grown vegetables. Here, we report a high‐quality genome sequence assembly, anchored to 11 chromosomes, with total length of 3.33 Gb and N50 scaffold length of 289.78 Mb. Most (92.91%) of the genome is composed of repetitive sequences, with 62.12% of 31 326 annotated genes confined to the terminal 20% of chromosomes. Simultaneous bursts of shared long‐terminal repeats (LTRs) in different Apiaceae plants suggest inter‐specific exchanges. Two ancestral polyploidizations were inferred, one shared by Apiales taxa and the other confined to Apiaceae. We reconstructed 8 Apiales proto‐chromosomes, inferring their evolutionary trajectories from the eudicot common ancestor to extant plants. Transcriptome sequencing in three tissues (roots, leaves and petioles), and varieties with different‐coloured petioles, revealed 4 and 2 key genes in pathways regulating anthocyanin and coumarin biosynthesis, respectively. A remarkable paucity of NBS disease‐resistant genes in celery (62) and other Apiales was explained by extensive loss and limited production of these genes during the last ~10 million years, raising questions about their biotic defence mechanisms and motivating research into effects of chemicals, for example coumarins, that give off distinctive odours. Celery genome sequencing and annotation facilitates further research into important gene functions and breeding, and comparative genomic analyses in Apiales.

## Introduction

Celery (*Apium graveolens* L. 2*n* = 2*x* = 22), a globally important crop (Browers and Orton, 1986; Li *et al*., 2014a), is a popular herb and vegetable. In North America, usually the crisp petioles (leaf stalks) are eaten as a vegetable. In Europe, the hypocotyl is used as a root vegetable. Leaf celery (Chinese celery), in East Asia, has thin stalks and a stronger flavour than other cultivars (http://www.upcscavenger.com/wiki/celery/). According to the USDA, celery is among the top 10 most consumed vegetables in the USA (after tomato, potato, onion, lettuce and similar to carrot, broccoli and cabbage), with nearly $314 millions of market value in 2017 (https://www.agmrc.org/commodities‐products/vegetables).

In addition to being an important vegetable, celery has many pharmacologically active compounds, including flavonoids, volatile oils, unsaturated fatty acids and others (Lin *et al*., 2007; Sellami *et al*., 2012). It is rich in coumarins and their derivatives (Najda *et al*., 2015; Numonov *et al*., 2018; Poumale *et al*., 2013; Zobel and Brown, 1990), having prominent roles in defending against pathogens (Carpinella *et al*., 2005; Chong *et al*., 2002; Figueroa‐Guinez *et al*., 2015; Sun *et al*., 2015) and multiple pharmaceutical activities, such as anticoagulation, antibacterial and anti‐inflammatory properties (Kontogiorgis *et al*., 2006; Stanchev *et al*., 2008).

Originating from the Mediterranean region and Middle East, the word ‘celery’ derives from ancient Greek. Celery dated to the seventh century B.C. was recovered in the Heraion of Samos, and to the ninth century B.C. in Greece (Megaloudi, 2005; Zohary and Hopf, 2000). Celery leaves and inflorescences were part of the garlands in the tomb of Tutankhamen, pharaoh of ancient Egypt. In Homer's Iliad, the horses of Myrmidons grazed on wild celery that grew in the marshes of Troy, and there was mention of meadows of wild celery surrounding the cave of Calypso in the Odyssey (Megaloudi, 2005).

As an Apiaceae plant, celery has an ‘umbel’ or umbrella‐like inflorescence (Fu *et al*., 2013). The Apiaceae contain about 466 genera and about 3,820 species (Mezghani *et al*., 2019; Plunkett *et al*., 2018). Some Apiaceae species are poisonous, including poison hemlock (*Conium maculatum*), water hemlock (*Cicuta maculata*) and fool’s parsley (*Aethusa cynapium*). However, in addition to celery, many Apiaceae species are widely used as vegetables, including parsley (*Petroselinum crispum*), carrot (*Daucus carota*) and fennel (*Foeniculum vulgare*). Apiaceae species used as herbs and spices include coriander (*Coriandrum sativum*), cumin (*Cuminum cyminum*), caraway (*Carum carvi*), dill (*Anethum graveolens*) and anise (*Pimpinella anisum*) (https://www.britannica.com/plant/Apiaceae).

Research on celery has mainly focused on phenotypes related to physiology, stress resistance, genetic diversity, gene expression and metabolites (Chen *et al*., 2017; Chen *et al*., 2019; Fu *et al*., 2014; Jia *et al*., 2015; Li *et al*., 2014b; Li *et al*., 2014a). Apiaceae comparative and functional genomic studies have been scarce – only the carrot genome has previously been sequenced, with 86% anchored to chromosomes (Iorizzo *et al*., 2016). To clarify Apiaceae biology and evolution, we recently produced a high‐quality chromosomal‐level assembly accounting for more than 95% of the estimated coriander genome, with total length of 2.11 Gb, and N50 scaffold length over 160 Mb (http://cgdb.bio2db.com) (Song *et al*., 2020; Song *et al*., 2019). Still more recently, a draft celery genome sequence, with scaffold N50 length of 35.57Kb, was made available (Li *et al*., 2020).

Here, we report a high‐quality chromosomal‐level genome sequence of celery, deciphered by integrating PacBio, Hi‐C and 10X Genomics technologies. The aims of the present research are to deduce the evolutionary trajectories of Apiaceae chromosomes and identify important gene families and genes regulating disease resistance and coumarins.

## Results

### Genome sequencing and assembly

The genome of the celery cultivar **‘**Ventura’ (*Apium graveolens* L.) was sequenced using several technologies (Table 1; Notes S1‐S2). We initially analysed the celery genome by Kmer = 17 using Illumina HiSeq4000 sequencing data with average 52.48X coverage depth (Table 1; Table S1). The heterozygosity rate was estimated to be 0.20%, the repetitive sequence percentage was 87.10%, and the genome size was 3.47 Gb (Figure S1; Table S2; Note S1). The PacBio Sequel I platform was used to produce a total of 269.85 Gb data with average 78.13X coverage depth (Table 1; Note S2). We obtained high‐quality and long PacBio reads with the N50 length of 15 066 bp, and the average read length was 9126 bp (Table S3). In addition, a 10X Genomics library was sequenced using an Illumina HiSeq4000, with an average 101.61X coverage depth. A total of 802.05 Gb (232.22X genome coverage) of celery DNA sequence was *de novo* assembled, with a cumulative scaffold length of 3.35 Gb and scaffold N50 of 2.53 Mb (Tables S4‐S9; Note S2).

**Table 1:** Summary of celery genome sequencing data

Paired‐end libraries	Insert size (bp)	Data size (Gb)	Read length (bp)	Coverage (X)
Illumina reads	350	181.27	–	52.48
PacBio reads	–	269.85	92 325/9126^†^	78.13
10X Genomics	–	350.93	150	101.61
Sub‐total	–	802.05	–	232.22
Hi‐C	–	378.06	–	109.46
Total	–	1180.11	–	341.68

^†^ The maximum and average length of the PacBio reads.

**Figure 1:**
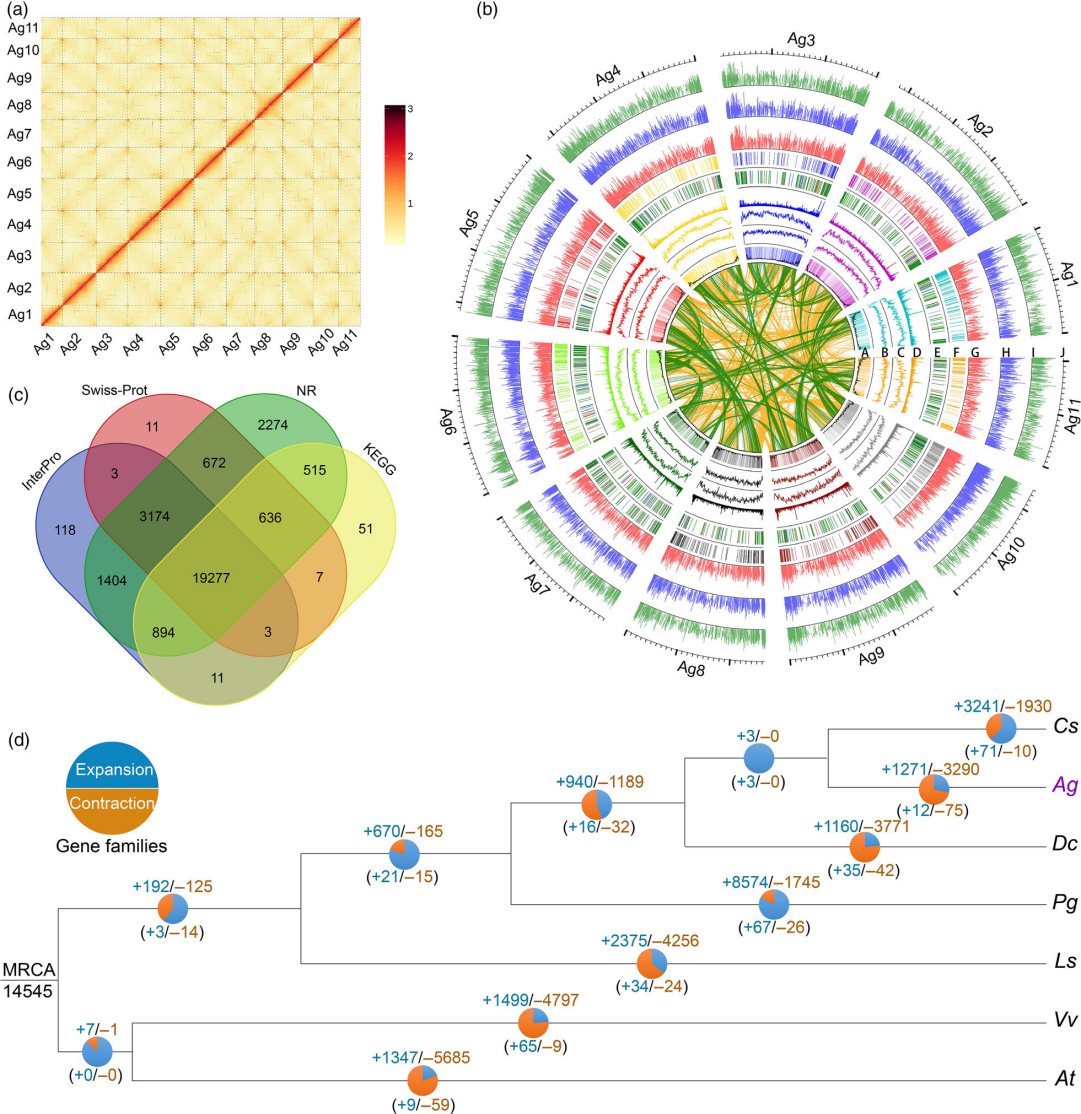
Hi‐C map, chromosomal features, function annotation and gene family analyses of celery genome. (a) Genome‐wide all‐by‐all interactions among all celery chromosomes (Ag1 to Ag11). (b) A, Gene density and distribution (non‐overlapping, window size, 50 kb); B, Density of Copia‐type transposons (non‐overlapping window size is 1 Mb); C, Density of Gypsy‐type transposons (non‐overlapping, window size, 1 Mb); D, Density of DNA repeats (non‐overlapping, window size, 1 Mb); E, Distribution of SSRs. The red, blue, green and grey lines represent P1, P2, P3 and other types of SSRs; F, Distribution of tandem genes; G, Gene expression levels (Log2FPKM) in celery roots; H, Gene expression levels (Log2FPKM) in celery petioles; I, Gene expression levels (Log2FPKM) in celery leaves; J, Celery chromosomes, showing colinear gene pairs produced by Apiaceae α (green lines) and ω events (orange). (c) Venn diagram of gene function annotations supported by 4 databases, including InterPro, Swiss‐Prot, NR and KEGG. (d) Gene family expansion/contraction. The blue and/or orange circles and corresponding numbers indicate gain (expansion) or loss (contraction) of gene families in different species, and the numbers in brackets indicate significantly expanded or contracted gene families (*P* < 0.05).

We conducted Hi‐C analysis to improve the genome assembly and obtained 378.06 Gb high‐quality sequences (Table 1; Tables S10‐S12; Note S3). A Hi‐C heat map was adopted to separate distinct regions on different chromosomes (Figure 1a). The revised assembly was 3.33 Gb, 95.96% of the estimated genome size, with contig N50 length reaching 790.58 Kb, and scaffold N50 of 289.78 Mb (Table 2; Tables S9‐S10), the largest scaffold N50 among 32 representative plant species recently sequenced (Table S13). A total of 3.047 Gb sequences, 91.50% of the revised genome, were anchored to 11 chromosomes.

**Table 2:** Statistics of celery genome assembly quality

Type	Length		Number	
	Contig (bp)^†^	Scaffold (bp)	Contig^†^	Scaffold
Total	3 323 719 648	3 332 579 003	9496	4863
Max	4 191 222	321 389 515	–	–
Number ≥ 2000	–	–	9334	4701
N50	790 578	289 786 985	1329	6
N60	643 053	272 646 905	1794	7
N70	500 788	267 513 317	2378	8
N80	357 519	207 320 945	3153	10
N90	202 303	207 269 490	4366	11

^†^ Assembled scaffolds >100 bp.

We obtained a relatively complete celery genome, and the mapping rate of reads reached 99.71% (Table S6). To assess the quality of genome assembly and annotation, the genome was examined by core eukaryotic gene mapping approach (CEGMA) and Benchmarking universal single‐copy orthologs (BUSCO) methods. The CEGMA analysis showed that 95.56% (237) of core eukaryotic genes were covered by assembled genome (Table S8). The BUSCO analysis indicated that 91.7%, 2.1% and 6.2% of 1,440 BUSCO genes were complete, fragmented and missing, respectively (Table S9).

### Genome annotation and gene family expansion analysis

By implementing *de novo* repeat prediction tools at Repbase, we found that 92.91% of the estimated celery genome was composed of repetitive sequences, more than twice that in carrot (46%) and consistent with the larger celery genome size (Figure 1b; Table S14). Most transposable elements (TEs) belonged to the long‐terminal repeat (LTR) category, with total length over 2.85 Gb, accounting for 85.75% of the whole genome (Table S15; Note S4). The two most frequent LTR types were Copia and Gypsy, respectively, accounting for 46.43% and 36.57% of the genome (Figure 1b; Figure S2). Retrotransposons and DNA transposable elements were nearly inversely distributed, with the former infrequent in gene‐rich terminal chromosomal regions, and the latter co‐occurring with genes, SSR and tandem clusters (Figure 1b). The LTR expansion in celery, coriander and carrot occurred after their split during evolution, and interestingly at similar times in different lineages, increasing their genome sizes in parallel (Figure S3). The occurrence of many highly similar LTR families between different Apiaceae plants, with divergence levels each corresponding to the same LTR burst, suggests inter‐specific exchanges of LTRs. Tandem, long interspersed nuclear element (LINE) and short interspersed nuclear element (SINE) repeat sequences only account for 4.75%, 1.10% and 0.01% of the celery genome, respectively (Figures S4‐S5; Tables S16‐S17; Note S4). Furthermore, 11 putative centromere regions were detected on the celery chromosomes. Both telomeres were predicted for 9 chromosomes, while only one telomere was detected in chromosomes Agr3 and Agr10 (Figure S6; Table S18).

Among 31,326 annotated celery genes (Figure S7; Table S19), non‐redundant protein (NR), Swiss‐Prot, KEGG and InterPro databases provided evidence of function for 29 050 (92.73%), with 19 277 annotated by all four databases (Figure 1c; Table S20, Note S4). Interestingly, 27.90%, 43.57% and 62.12% of genes occur in the 5%, 10% and 20% most terminal regions of chromosomes, respectively, as a result of repeat expansion in pericentromeric regions of celery (Figure S8a; Table S21). Similar phenomena were detected in the coriander genome, but contrasted with those of carrot, lettuce, Arabidopsis and grape. In addition, 327 miRNAs, 694 tRNAs, 649 rRNAs and 7,141 snRNAs were identified, accounting for 0.06% of the celery genome (Table S22), most (except snRNA) also in terminal chromosomal regions (Figures. S8b, S9; Table S23). Among rRNA genes, most (290) encode 5S rRNA, followed by 28S (187), 18S (121) and 5.8S (51) rRNAs (Table S22).

The distribution of gene numbers and family sizes was investigated in celery, two Apiaceae (coriander and carrot), one Apiales (ginseng: *Panax ginseng*) (Kim *et al*., 2018), one Asterid (lettuce: *Lactuca sativa*) (Reyes‐Chin‐Wo *et al*., 2017) and two other eudicots (the botanical model *Arabidopsis thaliana* and the genome structure model *Vitis vinifera*) (Figure S10a). In comparison to these taxa, celery has 530 species‐specific gene families, fewer than the 614 (carrot) to 2487 (ginseng) in the compared plants (Figure S10b). The 7 species have 26,293 gene families in total, including 9945 common gene families and 422 single‐copy ones. Notably, celery, carrot and coriander shared 863 Apiaceae‐specific gene families (Figure S10b). Among an inferred 14 545 gene families in their most recent common ancestor (Figure 1d), we detected 1271 gene family expansions in celery, similar to carrot (1160), but fewer than in coriander (3241) or ginseng (8574); and 3290 gene family contractions in celery, fewer than in carrot (3771), but more than in coriander (1930) and ginseng (1745). Expansion and contraction of 12 and 75 celery gene families, respectively, were statistically significantly (*P* < 0.05) (Table S24). The most expanded gene families were related to DUF, F‐box and ATP‐synthesis, while contracted gene families were mainly related to LRR, P‐kinase and p450 (Table S24).

### Genome organization and sequential polyploidization in Apiales plants

**Figure 2:**
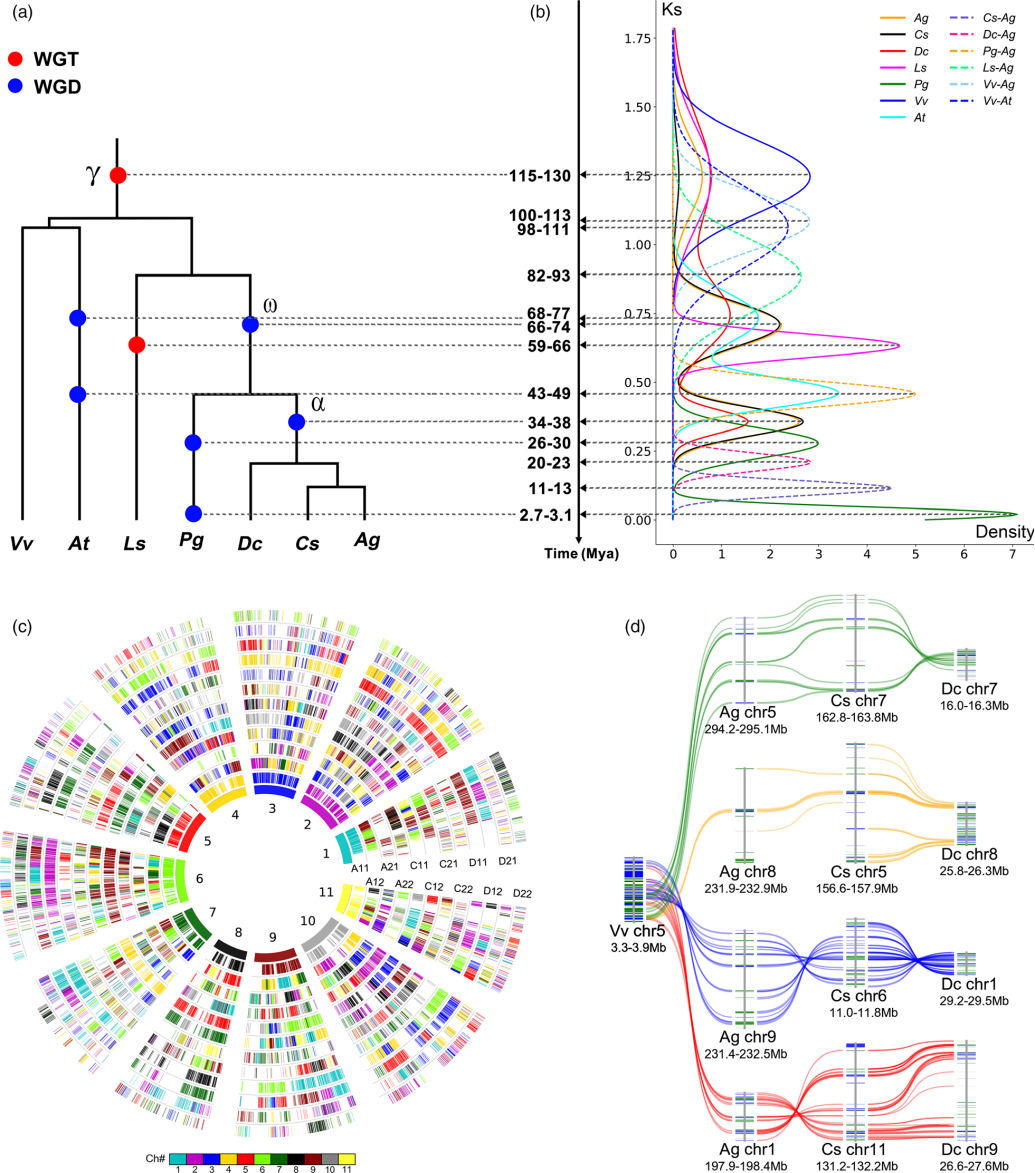
Evolutionary dating, global and local alignment of genomes. (a) Species phylogenetic trees for celery (Ag), coriander (Cs), carrot (Dc), *Panax ginseng* (Pg), Lettuce (Ls), Arabidopsis (At) and grape (*Vitis vinifera*, Vv). (b) Corrected number of synonymous substitutions per synonymous site (Ks) among colinear genes, within (continuous lines) and between genomes (dashed). Dates of polyploidization and speciation are estimated. (c) Global alignment of homologous regions in celery (A11, A12, A21, A22), coriander (C11, C12, C21, C22) and carrot (D11, D12, D21, D22) genomes with celery as a reference. The short lines forming the innermost celery chromosome circles represent predicted genes. Colinear genes are shown in the other circles, coloured as to chromosome number in their respective source plant, as shown in the inset colour scheme. (d) Local alignment of genes among grape (Vv), celery (Ag), coriander (Cs) and carrot (Dc). Using a grape segment as the reference, four colinear regions are detected in each of celery, coriander and carrot genomes. Chromosome numbers and location are shown.

Besides the whole‐genome triplication affecting most eudicots (γ, WGT) (Jaillon *et al*., 2007), two whole‐genome duplication (WGD) events (Apiaceae‐α and Apiaceae‐ω) shaped celery genome organization (Figure 2a,b; Figure S11a). We identified 394 homoeologous blocks within the celery genome, involving 9,834 pairs of colinear genes (Table S25). By inferring inter‐genomic gene colinearity, we mapped celery genome sequences onto selected eudicot genomes. The ratios of the best‐matched homologous regions between four species (grape, lettuce, carrot and coriander) and celery were 1:4, 3:4, 1:1 and 1:1, respectively (Figure 2c; Figure S11b,c, S12a‐e; Tables S26‐S28). This indicates that the Apiaceae species experienced two additional polyploidization events after their split from lettuce and other eudicots (Figure 2c,d; Figure S13). The celery homologous regions fell into two groups based on median synonymous substitution rates at synonymous sites (Ks) of colinear duplicated genes in each duplicated region (Table S26). The two groups of duplicated regions, containing 2452 and 3718 colinear gene pairs, cover 40.8% and 57.7% of the total genes, respectively (Tables S29‐S30).

Differences in Ks between colinear duplicated genes revealed divergent evolutionary rates among Apiaceae plants, with coriander evolving the slowest, and celery and carrot evolving 14.3% and 27.0% faster, respectively (Figure S14 and 15; Table S31). A correction‐by‐shared‐event approach was used to date the WGDs, with α (shared by the Apiaceae) inferred to occur ~34–38 and ω (shared by the Apiales) ~66–74 million years ago (Mya) (Figure 2a,b; Figure S15; Table S32). Accordingly, the divergence of coriander and celery was inferred to have occurred 11–13 Mya, consistent with the phylogenetically inferred date using the MCMCtree (Figure S16).

### Randomness of celery gene loss and gradual genome fractionation

The overwhelming majority of genes duplicated by the Apiaceae and Apiales WGDs have been lost. One grape gene would have 4 celery orthologs if there was no gene loss or translocation. The chromosomal regions duplicated by the WGDs often have divergent gene retention levels (Figure S17a‐c). Grossly, average loss rates of colinear celery genes were 47.78%, 50.09% and 69.95% using carrot, coriander and grape as reference, respectively, showing large‐scale genome fractionation and instability of the celery genome even after its split from other Apiaceae (Table S33). A total of 16%–20% of best‐matched genes between celery and the other two Apiaceae plants were not in their colinear locations. These genes might have been removed from their ancestral locations by TE‐associated translocations.

We investigated the scale and potential mechanisms of post‐polyploidization gene loss. In the celery genomic regions orthologous to a reference genome, intervening collinear orthologs, there were often genes in one genome without collinear orthologs in another genome. Respectively, 73.23%, 73.88% and 43.69% of non‐colinear genes from coriander, carrot and grape were singleton ones, or forming close neighbours of two, bordered by collinear genes (Figure S18; Table S34). This showed reciprocal genomic fractionation, and that a lower percentage of singleton and neighbouring non‐colinear genes with increasing evolutionary distance suggested a cumulative effect of gene removal to erode gene colinearity. Consecutive gene removals appeared at large random, as they could be modelled by geometric distributions. The extension parameters of geometric distributions were 0.26, 0.63 and 0.59 using grape, coriander and carrot as references, respectively (Figure S18; Table S35).

### Correlated gene expression between different‐coloured plants and balanced expression between subgenomes

To explore celery gene expression, RNA‐seq was performed, obtaining a total of 493 437 770 clean reads and 74.02 Gb sequencing data for root, petiole and leaf (Table S36; Note S5). In addition, we compared three celery varieties with different‐coloured petioles (green, red and white), obtaining another 441 271 066 clean reads and 66.18 Gb sequencing data (Table S37). Highly correlated gene expression was observed among three replications of each of these samples (Figure S19). More than 95% of reads could be mapped onto the celery genome, with more than 90% uniquely mapped and similar mapping ratios for the 3 varieties (Tables S38‐S39). A total of 26 930 (85.97%) celery genes showed expression in at least one tissue, while 4396 had no expression in all three tissues (Table S40; Figure S20). Similarly, 26 755 (85.41%) celery genes were detected in at least one variety, and 4571 genes had no expression in any of the three varieties (Table S41).

Furthermore, we detected 3207 common/shared differentially expressed genes (DEGs) among three tissues (Figure S21a, Note S5). A total of 85 common DEGs were identified among three celery varieties with different petiole colours, likely playing important roles in colour formation (Figure S21b). GO analysis showed that many DEGs between leaf and root were related to catalytic activity, while there was no related GO term enrichment of DEGs between leaf and petiole, or root and petiole (Figure S22). Similarly, the GO term of ‘catalytic activity’ was enriched for the DEGs between white and green, while no related GO term was enriched between red and green, or between red and white (Figure S23). The GO term ‘photosynthesis’ was also enriched for the DEGs between red and green or white and green celery varieties, while no related GO term was enriched between red and white. The KEGG analyses showed that DEGs related to biosynthesis of secondary metabolites were enriched among different tissues (Figure S24). The DEGs related with metabolic pathways were enriched between red and green, or between white and green, while no related KEGG term enrichment between red and white (Figure S25).

To compare expression patterns of duplicated copies of genes produced in Apiaceae α and ω duplications, homoeologous regions in celery were grouped into subgenomes A1‐A4 relative to mapped grape chromosomes (Note S6). Among all 4 subgenomes using grape as a reference, ~1% of duplicated genes showed clearly higher expression, ~10% showed clearly lower expression, and more than ~85% showed no significant differences using RNA‐seq of 3 tissues and 3 varieties of celery (Figure S26a‐f; Table S42, Note S6).

### Reconstructing ancestral karyotypes and deducing chromosome change trajectories

**Figure 3:**
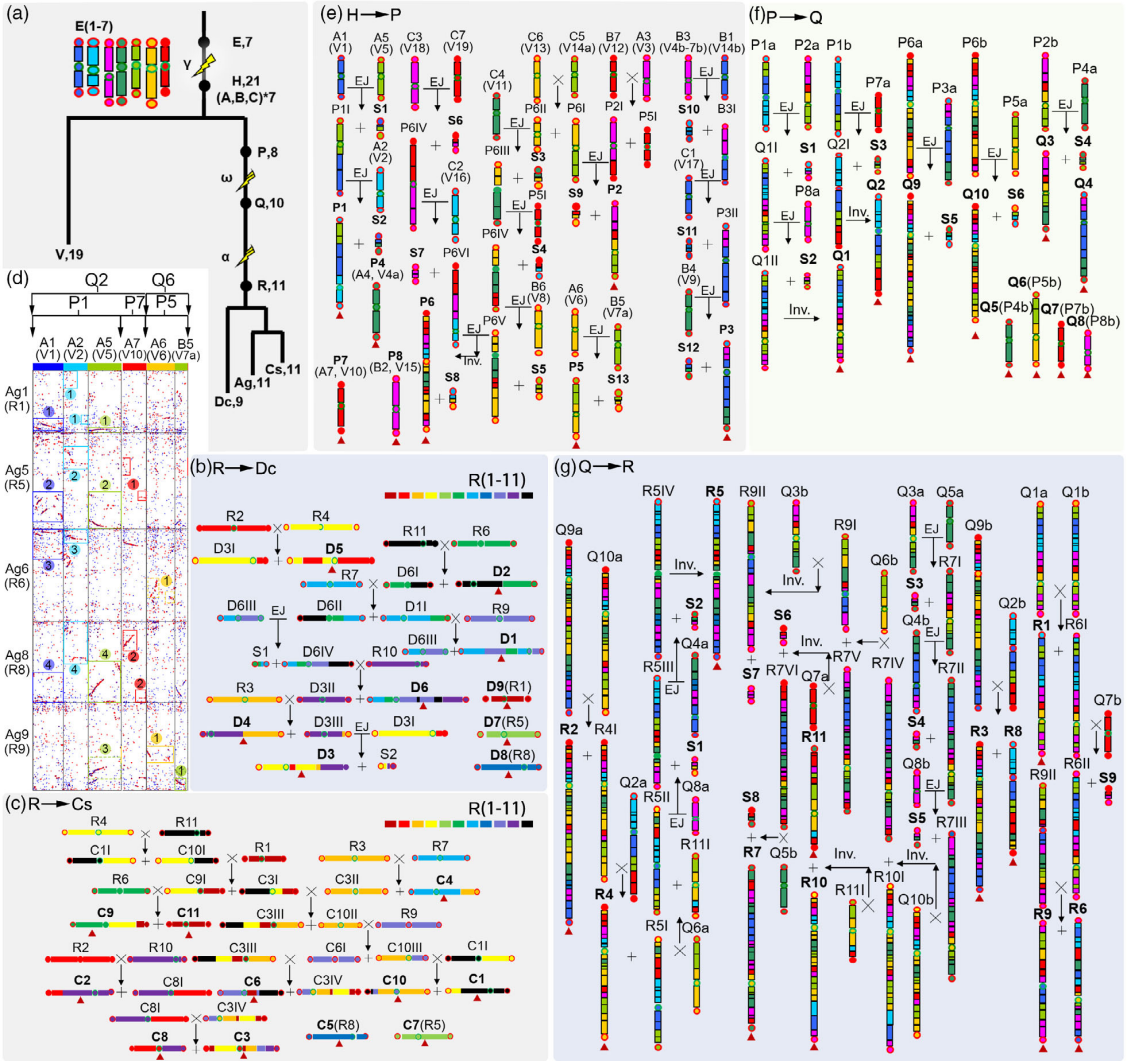
Evolutionary trajectories of the extant Apiaceae chromosomes. (a) Phylogenetic tree of the studied species. The seven haploid γ chromosomes, E1‐7, are shown following a previous colour scheme(Jaillon *et al*., 2007). After the γ, there had been 21 chromosomes (node H, the γ triplicated chromosomes: A1‐7; B1‐7; C1‐7), which reduced to 8 chromosomes (node P) before the Apiales‐common polyploidization (ω). These 8 P chromosomes might have duplicated to 16, then reduced to 10 (node Q) before the Apiaceae‐common polyploidization (α), then doubled to 20 which reduced to 11 (node R) Apiaceae proto‐chromosomes. These Apiaceae proto‐chromosomes were completely preserved in celery (Ag), but rearranged to produce the 11 coriander (Cs) and 9 carrot (Dc) chromosomes. Grape (V) chromosomes were used to infer the γ chromosomes; Flash marks are used to show polyploidizations. (b) Chromosome changes from R to Dc. For clearer illustration, a new colour scheme is adopted to represent the R chromosomes. (c) Chromosome changes from R to Cs. (d) A homologous gene dot plot between celery (with initials as Ag or R) chromosomes and the γ‐triplicated chromosomes (with initials as A or B), the latter of which were related to the derivative P and Q chromosomes; numbers in circles show duplicated regions in celery, being orthologous to corresponding γ chromosomes. Under each A or B chromosome code, the corresponding grape (V) chromosome is shown. (e) Chromosome changes from H to P. (f) Chromosome changes from P to Q. (g) Chromosome changes from Q to R. ‘X’ shows a crossing‐over between neighbouring chromosomes in a subfigure and after each crossing‐over, two newly produced chromosomes are shown with ‘+’. ‘EJ’ shows two chromosomes’ end to end joining. ‘Inv.’ shows segmental inversion. ‘a’ and ‘b’ after a chromosome code mean their being duplicated copies, such as Q1a and Q1b being duplicates of Q1. Roman numbers after chromosome code mean intermediate chromosomes, for example Q1I and Q1II are intermediate chromosomes; satellite chromosomes are shown with ‘S’, for example S1 being a satellite chromosome. Some ancestral chromosomes are preserved in descendent species, and they are just renamed, for example P7(A2) means that P7 is a direct derivative from the ancestral chromosome A2.

We reconstructed the Apiaceae proto‐chromosomes and the evolutionary trajectories by which they became extant chromosomes. Using homologous gene dot plots, we characterized the correspondence between genomes of Apiaceae plants and grape (Figure S12). The Apiaceae proto‐chromosomes were rather well represented by the modern celery chromosomes. The undisturbed integrity of celery chromosomes Ag1‐5 and Ag8 were evident from complete correspondence to carrot chromosomes (Figure S12a). The proto‐integrity of the other celery chromosomes is supported by homology with grape chromosomes (Figure 3a; Figure S12b). Taking celery chromosome Ag10 as an example, ignoring local reciprocal DNA inversions, over most of its length Ag10 shared orthology with grape Vv13, being paralogous to Vv6 and Vv8 due to the γ WGT (Figure S12b). In contrast, the same Ag10 region corresponds to regions in Dc3, Dc4 and Dc6 (Figure S12a), showing that Ag10 most likely preserved much the proto‐chromosome structure, while Dc3, Dc4 and Dc6 were rearranged after the Apium‐Daucus split. The remaining part of Ag10, merged from Vv16 (Figure S12b), was shared with the other Apiaceae (Figure S12c‐e). Re‐assembled, Ag10 could represent an Apiaceae proto‐chromosome.

By exploiting orthologous correspondence between genomes, we deduced the ancestral karyotypes at key evolutionary nodes and evolutionary trajectories to infer and draw extant chromosomes (Figure 3a). Starting from the 11 Apiaceae proto‐chromosomes, renamed R1‐11, corresponding to Ag1‐11 in order, we inferred how the carrot and coriander chromosomes formed. Specifically, crossing‐over between R6 and R11 produced Dc2 and an intermediate chromosome, which then sequentially crossed‐over with R7 and R9 to produce Dc1 (Figure 3b). Similarly, we reconstructed the evolutionary trajectories leading to the other carrot and coriander chromosomes (Cs1‐Cs11) (Figure 3b,c; Note S6). During the formation of carrot chromosomes, two putative satellite or B chromosomes (S1‐2), each formed mainly by the two telomeres, might have been produced but lost, resulting in chromosome number reduction.

The Apiaceae proto‐chromosomes were compared to grape chromosomes to deduce karyotypes before and after Apiaceae α and ω polyploidization events (Figure 3a). The 19 grape chromosomes were used to reconstruct 21 proto‐chromosomes of early eudicot plants (A1‐A7; B1‐B7; C1‐C7), tripled from 7 pre‐γ proto‐chromosomes: E1‐E7 (Figure 3a; Table S43; Note S6). Repetitive co‐occurrence of the 21 post‐γ chromosomes in the celery chromosomes permitted deductions about the relative timing of rearrangements. That is, if two or more grape chromosomes showed common homology four times to celery chromosomes, parsimony suggests that they had merged before the ω (Figure 3d). In contrast, if two or more grape chromosomes showed corresponding homology only two times in celery chromosomes, they most likely had merged after the ω but before the Apiaceae α. For example, the post‐γ chromosomes A5, A1 and A2 coincided in each of Ag1, Ag5, Ag6 and Ag8, which could be explained by their fusion into a proto‐chromosome P1 before the ω (Figure 3d). A segment of A5 unexpectedly appearing in Ag9 but not in Ag6 as part of a P1 duplicate could be explained by accidental crossing‐over between the P1 duplicate and a P5 duplicate, mainly formed by A6 and the part of B5 (Figure 3e,f). In contrast, A7 appeared twice in homologies with R5 and R8, but not in R1 or R6, which implied that after ω as part of another proto‐chromosome P7, A7 fused with P1, and formed a relatively recent chromosome Q2 before α (Figure 3d,f). After the Apiaceae α, Q2 duplicated to produce Q2a and Q2b, with the former crossing‐over with an intermediate chromosome R4I to produce R4, and with the latter crossing‐over with Q9b (formed by steps of fusion or crossing‐over) to make R3 and R8 (Figure 3g).

In summary, from γ chromosomes we inferred the formation of 8 P chromosomes before the ω, about 10 Q chromosomes after the diploidization following ω and before the Apiaceae α, and about 11 R chromosomes after diploidization following the Apiaceae α that became the extant Apiaceae chromosomes (Figure 3).

### Significant reduction and even halted production of disease‐resistant genes during last ~ ten million years in celery

**Figure 4:**
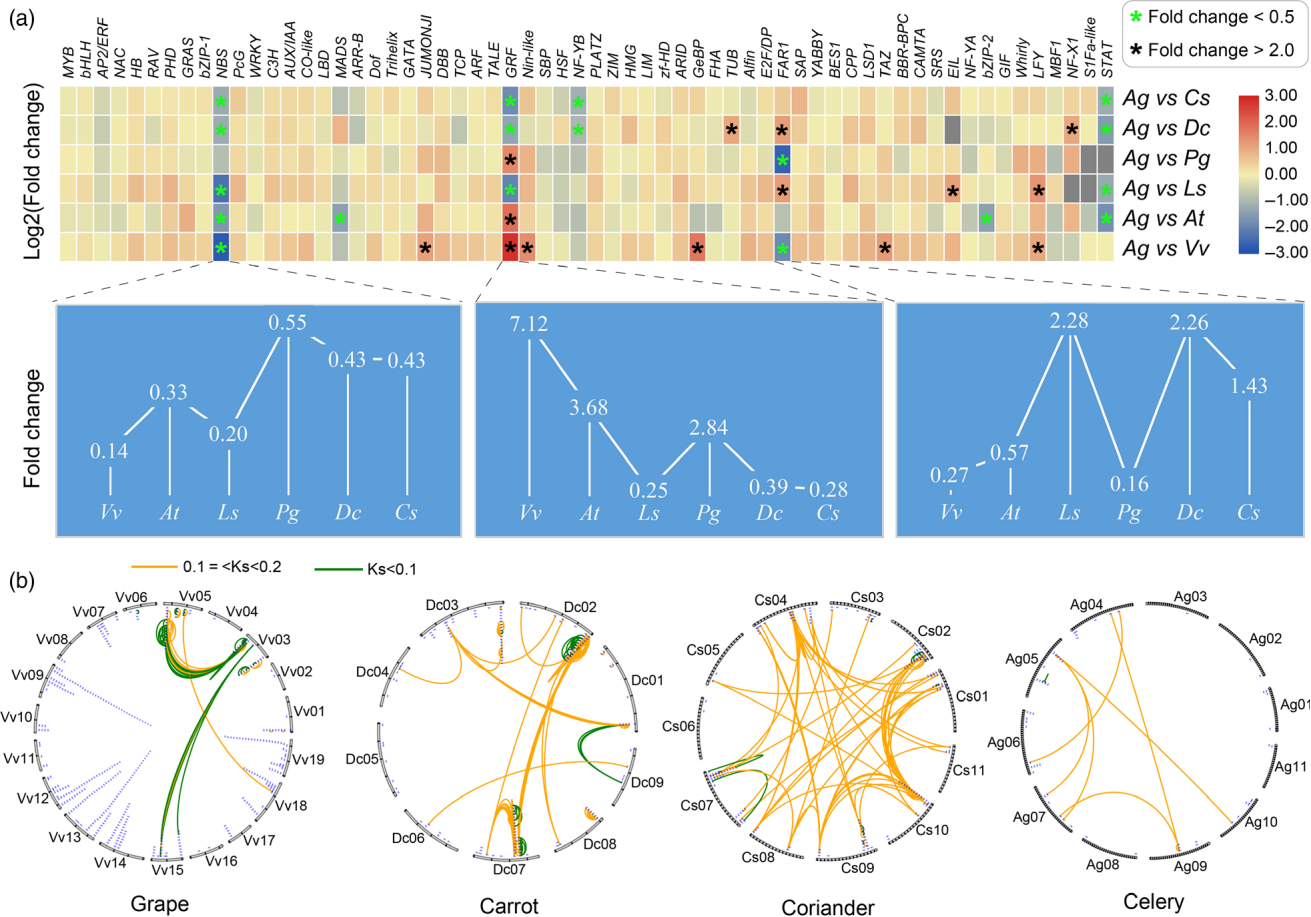
Comparative analysis of the transcription factor families of celery and 6 representative plants. (a) The heatmap constructed by the transcription factor number of fold change between celery and 6 other species. The black or green asterisk represents the fold change greater than 2.0 or lower than 0.5, respectively. The line chart represents the partial enlarged map of three gene families, including *NBS*, *GRF* and *FAR1*. (b) The distribution of *NBS* family genes on the chromosomes of celery and 3 other species. Lines represent Ks values of *NBS* gene pairs that are less than 0.1 (green); or larger than 0.1 but less than 0.2 (orange).

A total of 2090 transcription factor (TF) genes in 62 families were identified in the celery genome, with the most being members of the *MYB* (240), basic helix‐loop‐helix genes (*bHLH*, 131) and *APETALA2*/ethylene response factor (*AP2*/*ERF*, 129) families, mainly involved in stress tolerance, growth and development (Figure S27a; Table S44; Figure S28a,b). The number of members of the growth‐regulating factor (*GRF*) gene family was more in celery than in grape, Arabidopsis and ginseng, but less than in coriander, carrot and lettuce. The far‐red‐impaired response (*FAR1*) gene family was larger in celery than carrot and lettuce, but smaller than in ginseng and grape (Figure 4a; Figure S27b; Figures. S29a‐c, S30a,b; Note S6).

Despite its large genome size, celery has only 62 *NBS* genes, including 10 TNL, 44 CNL and 8 RNL subtypes, respectively, far fewer than grape (442), lettuce (392), ginseng (215), carrot (148), or coriander (189) (Figure S27b; Figure 4a; Table S45). Indeed, among 106 plant species, celery has fewer *NBS* genes than most (Figure S31; Table S46). Only two pairs of celery *NBS* genes have Ks < 0.1 (i.e. diverged in the past ~10 million years), and only six have 0.1 < Ks < 0.2 (Figure 4b), diverged in the past ~20 million years.

Although other disease‐resistant genes, for example encoding receptor‐like proteins (*RLP*) and receptor‐like kinase (*RLK*), have copy numbers in celery similar to those in other plants, extreme paucity of celery NBS genes is consistent with a general trend among Apiaceae (Figure S32; Table S47). Based on inferred colinearity, a group of 25 relatively old *NBS* genes on coriander chromosome CS02 correspond to only 2 *NBS* genes at the orthologous region on celery chromosome AG02, implying loss of at least 23 celery *NBS* genes at this location, with similar losses inferred on several other chromosomes (Figure 4b). Similarly, only 5 and 22 new NBS gene duplication events were found in coriander and carrot in the past ~10 million years, but large‐scale gene loss occurred in the two species. For example, 12 *NBS* genes on AG06 correspond to 4 *NBS* genes on CS09, implying at least 8 coriander genes lost (Figure 4b). Phylogenetic analysis of Apiaceae *NBS* genes also showed large‐scale gene loss, many branches having only a singleton celery gene and some with none (Figure S33).

### Exploration of key genes in anthocyanin and coumarin biosynthesis pathway

**Figure 5:**
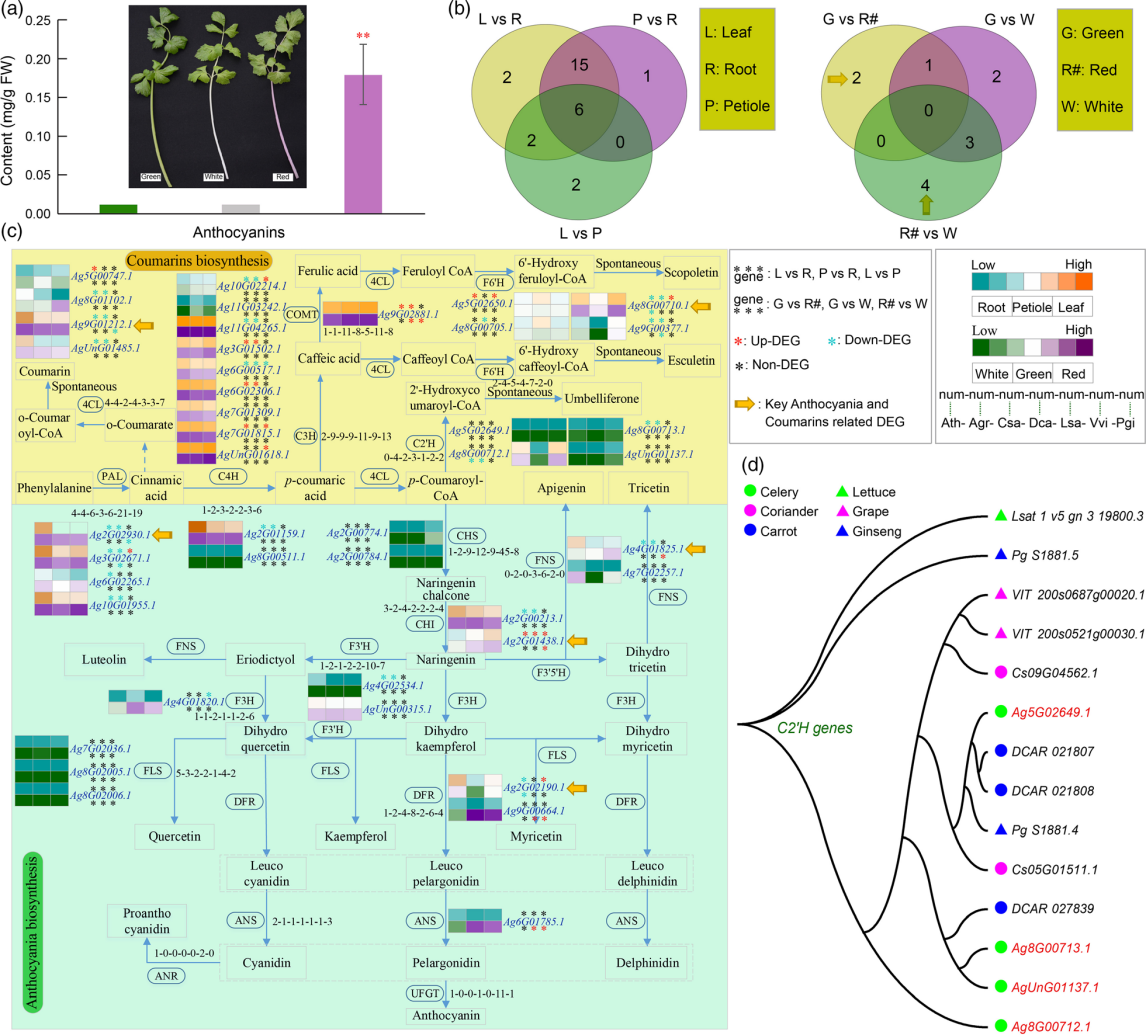
Inferred celery anthocyanin and coumarin biosynthesis genes. (a) Content (mg/g) of anthocyanins of three celery varieties with different colour petiole. The red asterisk represents significantly different (*P* < 0.01) content between the red and other two varieties. (b) The Venn diagrams show the DEGs among three tissues (root, leaf and petiole) and three varieties (green, red and white). (c) The anthocyanin (green background) and coumarin (yellow background) biosynthesis gene identification in celery and other 6 species. The notation ‘1‐1‐1‐1‐1‐1‐1’ indicates that one gene was identified in Arabidopsis, celery, coriander, carrot, lettuce, grape and ginseng, respectively. Gene expression was detected in the 3 different tissues (root, leaf and petiole) and 3 different colours’ varieties of celery. The orange and purple colours indicate high expression level in different tissues and varieties, respectively. Asterisks represent the up‐DEGs (red), down‐DEGs (blue) and non‐DEGs (black). The yellow arrow represents the key anthocyanins and coumarins related DEGs between red and green/white varieties. (d) The phylogenetic tree constructed using *C2'H* genes from celery and other five species.

The average anthocyanin content in red celery varieties (0.1790 mg/g) was 15.6 and 16.0 times (*P*‐value < 0.01) more than in green (0.0115 mg/g) and white (0.0112 mg/g) varieties (Figure 5a; Tables S48 and S49). No significant difference was found between white and green varieties.

The sequences for 30 Arabidopsis genes encoding 17 enzymes implicated by KEGG in plant anthocyanin and coumarin biosynthesis were used as seeds to identify homologs in celery and six other species (Figure 5; Table S50). Most nodes in the regulatory pathway have one or more gene copies among the 7 species. There were 4 trans‐4‐coumaroyl‐CoA 2‐hydroxylase (*C2'H*) genes in celery, more than in the other six species (Figure 5c). The gene *Ag8G00712.1* was differently expressed between the green and red varieties (Figure 5c,d), suggesting a role in celery coumarin biosynthesis variation. More generally, among a total of 43 anthocyanin and coumarin biosynthesis‐related genes in celery, 6 were differentially expressed among leaf, root and petiole tissues (Figure 5b; Table S51), including four and two for anthocyanin and coumarin biosynthesis pathways, respectively.

An inferred regulatory network (Figure S34) involved 77 differently expressed transcription factors (DETs) and 5 key anthocyanin and coumarin related DEGs. The DETs in the network were classified into 33 categories, of which *MYB* genes had the most TFs (7), followed by *bZIP*‐1 and *MADS*. There were more network connections (99) showing positive regulation (PCC > 0.9) between DEGs and DETs than negative regulation (77) (PCC < −0.9) (Table S52).

## Discussion

A high‐quality chromosome‐level celery reference genome and transcriptome, together with genomic data for carrot and coriander, provide resources for both fundamental and applied research into celery and other Apiaceae plants as well as new insights into the evolution of a group of under‐explored plant taxa.

To cope with the celery genome complicated with repeat sequences, we selected the Falcon software to assembly, which is well suited to assembling of PacBio reads and works well for such a complex genome (Chin *et al*., 2016; Michael *et al*., 2018; Ruan and Li, 2020). As to evaluating assembly standards, Falcon software is based on its own algorithms (Chin *et al*., 2016). Here, we obtained a relative integrative genome sequence using the Falcon software. Furthermore, we conducted sequence consistency assessment, sequence integrity assessment using CEGMA and BUSCO (Tables S5‐S9), which comprehensively reflected the good quality of our assembled genome (Parra *et al*., 2007; Simao *et al*., 2015; Korlach *et al*., 2017; Waterhouse *et al*., 2018).

The curious finding of limited production of new NBS duplicates and large‐scale removal of existing NBS duplicates in the last ~10 million years or more raised questions about how Apiaceae plants withstand diseases and pests (Wan *et al*., 2019). One option may be the production of chemicals with odours that repel potential pests. Different Apiaceae plants have been exploited to produce fumigants against pests (BIBI *et al*., 2010; López *et al*., 2008; Mahboubi *et al*., 2010; Mukesh Kumar, 2008). Indeed, while some vegetable extracts have highly attractive effects on whitefly adults, such as cabbage and tomato, celery essential oil exhibits a repellent effect (Li *et al*., 2014c). Celery seed‐derived oil has been extracted to repel lesser grain borer, dengue vector, mosquito and bacteria (Baananou *et al*., 2013; Ebadollahi, 2018; Kumar *et al*., 2014; Tuetun *et al*., 2004). In addition, celery and other Apiaceae species are rich in coumarins and their derivatives (Najda *et al*., 2015; Numonov *et al*., 2018; Poumale *et al*., 2013; Zobel and Brown, 1990), a major group of plant secondary metabolites with multiple pharmaceutical activities and important roles in defence against pathogens (Carpinella *et al*., 2005; Chong *et al*., 2002; Figueroa‐Guinez *et al*., 2015; Kontogiorgis *et al*., 2006; Stanchev *et al*., 2008; Sun *et al*., 2015). Here we showed that several genes of the coumarin biosynthesis pathway in celery and some other Apiaceae species were expanded in copy number compared with Arabidopsis and other studied non‐Apiaceae species.

Two sequential WGDs leading to the formation of celery and other Apiaceae genomes exerted great impact on gene regulatory networks and may have contributed to diversification of the Apiaceae (Iorizzo *et al*., 2016; Plunkett *et al*., 2018; Song *et al*., 2020). Average colinear gene removal rates in celery were ~50%–70% relative to different reference genomes, respectively, showing high instability since these Apiaceae taxa diverged. A cross‐genome best‐matched gene search showed that about three‐fourths of gene removals may be caused by deletion from celery, the remainder being gene relocation in the genome. Further analysis of the length of DNA containing removed genes in celery and other Apiaceae suggested a segment‐by‐segment removal model (Cheng *et al*., 2018; Freeling *et al*., 2015), with progressive accumulation resulting in genome fractionation after polyploidization. High and continuous gene removals show the instability of the early Apiaceae genome, conferring enormous opportunities for natural selection, plant divergence and new species formation. In an effort to reconstruct plant chromosome evolution in the deep past, inferences herein about the ancestral karyotypes from early eudicots to extant plants are valuable to understanding chromosome biology.

Transcriptome analysis contributes to understanding of both the celery evolutionary generally, and functions of specific genes salient to important traits. Approximately balanced gene expression was observed between duplicated copies of chromosomes produced in Apiaceae α and ω duplications, with little evidence of ‘expression dominance’ by one subgenome. The molecular basis of celery anthocyanin and coumarin biosynthesis regulatory networks were comprehensively analysed in combination with transcriptome analysis. Several key genes related to their biosynthesis were identified in celery and their expression was explored between different tissues and varieties, laying a solid foundation for dissecting genetic mechanisms associated with their production.

## Materials and methods

### Genome sequencing

Leaf samples were collected from *Apium graveolens* ‘Ventura’ and processed for genomic DNA isolation and library construction. General standards and methods were used for DNA extraction by Tris‐Phenol‐Chloroform. The following three sequencing strategies were used: (a) Library construction included 2 paired‐end Illumina libraries (Illumina Inc, CA, USA) with 350 bp fragments. In total, 181.27 Gb clean data were obtained, which covered the genome ~52.48X. The 17nt k‐mers was used to estimate the genome size (Marcais and Kingsford, 2011). (b) Third‐generation libraries were constructed according to PacBio protocol and sequenced using PacBio Sequel platform (Pacific Biosciences, CA, USA). In total, 269.85 Gb clean data were obtained, ~78.13 X coverage of the genome. (c) 10X Genomics Library construction: 350.93 Gb clean data were obtained, ~101.61 X coverage. Sequencing was performed by the Novogene Corporation.

### Hi‐C technology

Hi‐C technology spatially connects DNA sequences according to interactions between distantly located DNA fragments at physical locations. The interaction probability is higher within the chromosome than between chromosomes and decreases with increased distance on the same chromosome, permitting sorting and orienting contigs or scaffolds along a chromosome. The Hi‐C analysis mainly included the following three steps: (i) comparison with draft genome, (ii) clustering, (iii) sorting and orientation.

### Genome assembly

The process and software parameters for the celery genome assembly were mainly as follows. The Falcon software (https://github.com/PacificBiosciences/FALCON) was used for the genome assemble with the parameters, falcon_sense_option = ‐‐output_multi ‐‐min_idt 0.70 ‐‐min_cov 3 ‐‐max_n_read 300 ‐‐n_core 20 overlap_filtering_setting = ‐‐max_diff 500 ‐‐max_cov 500 ‐‐min_cov 2 ‐‐bestn 10 ‐‐n_core 36 (Chin *et al*., 2016). The genome error correction was conducted using second and third sequencing data by Pilon (https://github.com/broadinstitute/pilon/wiki) and Quiver software with the default parameters, respectively (Chin *et al*., 2013; Walker *et al*., 2014). The 10X technology was used for assisting genome assembly using fragScaff software (https://sourceforge.net/projects/fragscaff/files/) with the parameters, ‐fs1 '‐m 3000 ‐q 30 ‐E 30000 ‐o 60000' ‐fs2 '‐C 5' ‐fs3 '‐j 2 ‐u 3'(Adey *et al*., 2014). Hi‐C assisted genome assembly using the software LACHESIS (https://github.com/shendurelab/LACHESIS) with the parameters, CLUSTER_N = 11, CLUSTER_MIN_RE_SITES = 583, CLUSTER_MAX_LINK_DENSITY = 9, CLUSTER_NONINFORMATIVE_RATIO = 0 (Burton *et al*., 2013). The CEGMA and BUSCO pipelines were used to assess the assembled genome with default parameters (Parra *et al*., 2007; Simao *et al*., 2015).

### Gene prediction

Firstly, we used multiple gene prediction methods, including homologous prediction, *de novo* prediction and other evidence‐supported predictions. (i) Homologous prediction was conducted using Blast (http://blast.ncbi.nlm.nih.gov/Blast.cgi) and Genewise (http://www.ebi.ac.uk/~birney/wise2/) programs with default parameters (Birney *et al*., 2004; Camacho *et al*., 2009). (ii) *De novo* prediction mainly used Augustus (http://bioinf.uni‐greifswald.de/augustus/), GlimmerHMM (http://ccb.jhu.edu/software/glimmerhmm/) (Stanke and Morgenstern, 2005) and SNAP (http://homepage.mac.com/iankorf/) software packages with default parameters (Korf, 2004). (iii) Other evidence‐supported predictions used EST and cDNA data from homologous species by Blat program (http://genome.ucsc.edu/cgi‐bin/hgBlat) with default parameters (Kent, 2002). Secondly, we integrated the above results into one non‐redundant gene set using the IntegrationModeler (EVM, http://evidencemodeler.sourceforge.net/) with default parameters (Haas *et al*., 2008). Finally, we integrated the above results and our RNA‐seq data using PASA (http://pasapipeline.github.io/) with default parameters (Haas *et al*., 2003).

### Genome annotation

Genome annotation in this study mainly involved the following three parts: (i) *Repeated sequence annotation*. Two methods, homologous sequence alignment and *de novo* prediction were used. Homologous sequence alignment was mainly based on the repeat sequence database (RepBase, http://www.girinst.org/repbase), and using Repeatmasker and repeatproteinmask programs (http://www.repeatmasker.org/) to identify repeat sequences (Bao *et al*., 2015; Tarailo‐Graovac and Chen, 2009). *De novo* prediction firstly built the repeat sequence database using Piler (http://www.drive5.com/piler/) (Edgar and Myers, 2005), LTR_FINDER (http://t life.fudan.edu.cn/ltr_finder/) (Xu and Wang, 2007), RepeatModeler (http://www.repeatmasker.org/RepeatModeler.html) and RepeatScout software (http://bix.ucsd.edu/repeatscout/) (Price *et al*., 2005), then the Repeatmasker program was run to perform the prediction. Tandem repeats were predicted using the TRF software (http://tandem.bu.edu/trf/trf.html) (Benson, 1999). (ii) *Gene annotation*. It was mainly conducted by comparing with known protein databases, including TrEMBL, Swiss‐Prot, InterPro and KEGG. (iii) *non‐coding RNA annotation*. tRNAscan‐SE program (http://lowelab.ucsc.edu/tRNAscan‐SE/) was used to identify tRNAs (Chan and Lowe, 2019). The INFERNAL program (http://infernal.janelia.org/) was used to predict miRNAs and snRNAs (Nawrocki and Eddy, 2013), and the rRNAs were predicted by Blast. Centromeres were predicted using the distribution of repeated sequences on chromosomes according to a previous report (Melters *et al*., 2013). Telomeres were identified using SERF to find repeated sequences (bioserf.org) (Somanathan and Baysdorfer, 2018).

### Transcriptome sequencing

Samples of celery were collected from 3 different‐coloured petiole varieties, including green celery ‘Ventura’, white celery ‘Baiqin’ and red celery ‘Hongqin’. Three tissues (root, petiole and leaf) of green celery ‘Ventura’ were also used for RNA‐seq analyses. Each sample had three biological replicates. RNA was isolated from the samples using a kit (Tiangen, Beijing, China) based on the manufacturer’s instructions. The main steps of RNA‐seq contained the following four steps: (i) RNA sample quality check; (ii) Library construction; (iii) Library inspection; (iv) Sequencing and bioinformatics analysis.

Clean reads were aligned to the celery genome by HISAT software with default parameters (http://www.ccb.jhu.edu/software/hisat/index.shtml) (Kim *et al*., 2015). The novel transcripts were predicted by Cufflinks with default parameters, and FPKM (Fragments Per Kilobase of transcript sequence per Millions base pairs) was used for calculating gene expression values (Trapnell *et al*., 2010). The HTSeq program was used to analyse gene expression with default parameters (Anders *et al*., 2015), and the DESeq software was used to conduct DEGs analyses with *P*‐adj < 0.05 and |log2(fold change)| > 1 (Anders and Huber, 2010). In addition, the content anthocyanin was measured by spectrophotometry from the petiole of these three celery genotypes.

### Gene family’ identification, amplification and contraction

We conduct gene family identification using OrthoFinder according to the following steps (Emms and Kelly, 2019): (i) Filter gene set of each species. Only the longest transcript was retained when a gene had multiple alternative splicing transcripts, excluding genes that encode peptides of less than 50 amino acids. (ii) Obtain similarity relationships between protein sequences of all species by Blastp (e‐value < 1e‐5). (iii) Compare sequences and conduct cluster analysis using MCL graph clustering algorithm, obtaining single‐copy and multi‐copy gene families. Gene family amplification and contraction analysis was performed using CAFE software with default parameters (De Bie *et al*., 2006).

### Inference of gene colinearity, Ks calculation, distribution fitting and correction

Colinear genes were inferred using ColinearScan (Wang *et al*., 2006). Firstly, BlastP searches were performed to find putative homologous genes within a genome or between genomes. When running ColinearScan, maximal gap length between neighbouring genes in colinearity along a chromosome sequence was set to 50 genes according to previous reports (Wang *et al*., 2016a; Wang *et al*., 2005; Wang *et al*., 2017a; Wang *et al*., 2015). Since large gene families lead to difficulty to infer gene colinearity, families with >30 genes were removed before running ColinearScan.

Secondly, homologous gene dot plots were produced using MCScanX tool kit (Wang *et al*., 2012). Dot plots were used to facilitate identification of homologous blocks produced by different polyploidization events. Ks values were estimated between colinear homologous genes by using YN00 program in the PAML (v4.9h) package with the Nei‐Gojobori approach (Yang, 2007), and the median Ks of colinear homologs in each block was shown to help group blocks produced by different events.

Thirdly, the probability density distribution curve of Ks was estimated using MATLAB with the kernel smoothing density function. Multi‐peak fitting of the curve was performed using the Gaussian approximation function (cftool) in MATLAB, and the coefficient of determination (R‐squared) was set to be at least 0.95 (For details, see Note S6).

Fourthly, we performed a correction to have a common evolutionary rate to conduct reasonable dating (For details, see Note S6). Here, different from previous practice (Wang *et al*., 2017b; Wang *et al*., 2016b), we performed a two‐step rate correction according to the fact that celery, carrot and coriander shared two extra polyploidizations after the split with lettuce. In the first step, we managed to correct evolutionary rate by aligning the Ks distributions of celery, coriander, lettuce and carrot γ duplicates to that of grape γ duplicates, which have the smallest Ks values. Then, according to the result that celery being of the slower rate with the two extra polyploidizations, we re‐corrected the evolutionary rates of celery α produced duplicates with coriander as the reference.

Eventually, to construct the table with the grape genome as a reference, all grape genes were listed in the first column. Each grape gene may have two additional colinear genes in its genome due to WGT event, and two other columns in the table listed this information. For a grape gene, when there was a corresponding colinear gene in an expected location, a gene ID was filled in a cell of the corresponding column. When it was missing, often due to gene loss or translocation, the cell contained a dot. For the lettuce genome, with whole‐genome triplications, we assigned three columns. For the carrot, coriander or celery genome, each affected by two paleo‐polyploidization events, we assigned four columns. Therefore, the table had 48 columns, reflecting layers of tripled and then fourfold homology due to recursive polyploidies across the genomes (For details, see Note S6).

### Reconstructing ancestral karyotypes of Apiales plants

Gene colinearity between compared genomes could reflect karyotype changes and even uncover the trajectories of the formation of extant chromosomes. By checking homologous gene dot plots, we compared the Apiaceae genomes and the grape genome, deduced their ancestral chromosomes at key evolutionary nodes, for example before their divergence and before or after polyploidizations, and deduced the evolutionary changes from ancestral chromosomes to extant chromosomes. As to previously proposed genetic model (Wang *et al*., 2016b), implemented in grasses, Arabidopsis and legumes (Wang *et al*., 2016b; Zhuang *et al*., 2019), the extant or derivative chromosomes at a relatively recent node came from fusions or crossing‐overs of ancestral chromosomes, usually including exchanging arms of two chromosomes, ‘end to end joining’ of two different chromosomes, and ‘nested chromosome fusion’ with one chromosome inserted into another one. The latter two types of changes involved the production of satellite or B chromosomes, and the loss of which resulted in chromosome number reduction (For details, see Note S6).

## Conflicts of interest

The authors declare no competing interests.

## Authors contributions

X.W. and X.S. conceived the project and were responsible for the project initiation. X.S., P.S. and J.Y. supervised and managed the project and research. Experiments and analyses were designed by X.S., K.G., N.L., N.L., W.C., F.N., X.L., J.H., Q.Y., C.L. and S.F. Bioinformatic analyses were led by X.W., X. S., P. S., J.Y., T.L. and K.G. Data generation and analyses were performed by F.M., Z.Z., X.L., J.H., Q.Y., B.J., F.N., J.W., W.C., S.F., L.S., M.L., Z.Q., T.W. and R.K.V. The manuscript was organized, written and revised by X. S., X. W., R.K.V., X.L., A.H.P., H.L., P.S., J.Y., Q.P., T.Y., X.K., W.Z., C.C. and Y.Y. All authors read and revised the manuscript.

## Supporting information


**Fig S1‐S34.** Supplementary Figs. 1‐34.

**Table S1‐S52.** Supplementary Tables 1‐52.

**Note S1.** Supplementary Notes 1‐6.

## Data Availability

This whole‐genome shotgun project has been deposited at DDBJ/ENA/GenBank under the accession WRXP00000000. The version described in this paper is version WRXP01000000. The genome sequence and RNA‐seq datasets of celery reported in this paper have been deposited in the Genome Sequence Archive (Wang *et al*., 2017c) in BIG Data Center (Members, 2019), Beijing Institute of Genomics (BIG), Chinese Academy of Sciences, under accession numbers CRA001993, CRA001996, CRA001997 that are publicly accessible at http://bigd.big.ac.cn/gsa. The assembled celery genome and related dataset also can be downloaded from our celery genome database (CGD: http://celerydb.bio2db.com). All materials and related data in this study are available upon request.
