# Supplementary material for: The celery genome sequence reveals sequential paleo‐polyploidizations, karyotype evolution and resistance gene reduction in apiales
Source: Plant Biotechnol J. 2020 Nov 18;19(4):731–44. doi: 10.1111/pbi.13499 (PMC8051603; doi:10.1111/pbi.13499)
Supplement: Supplementary file 1 — Fig S1‐S34. Supplementary Figs. 1‐34. [file PBI-19-731-s003.pdf]

Supplementary Figures 1-34

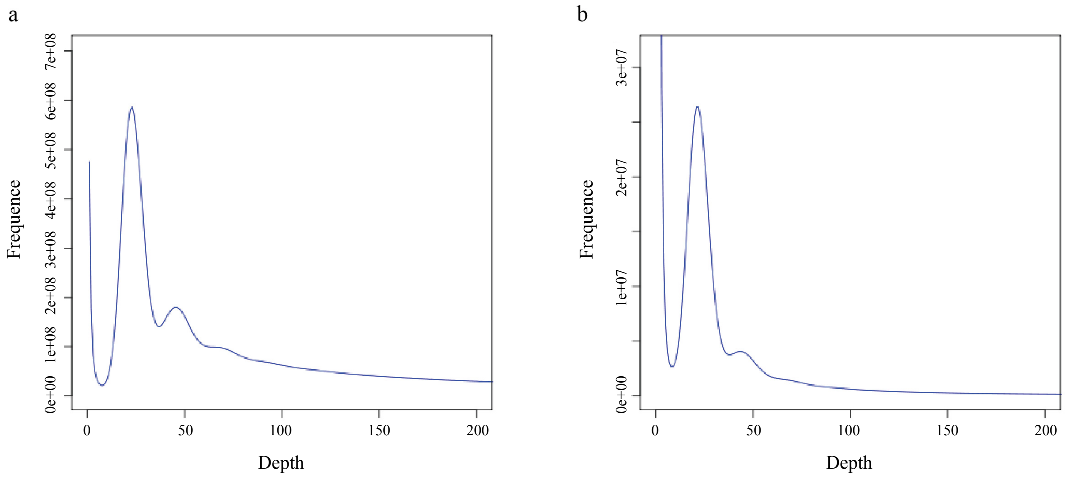

Supplementary Figure 1. K-mer distribution of the celery genome. (a) K-mer=17 Depth and K-mer number frequency distribution. (b) K-mer=17 Depth and K-mer type frequency distribution.

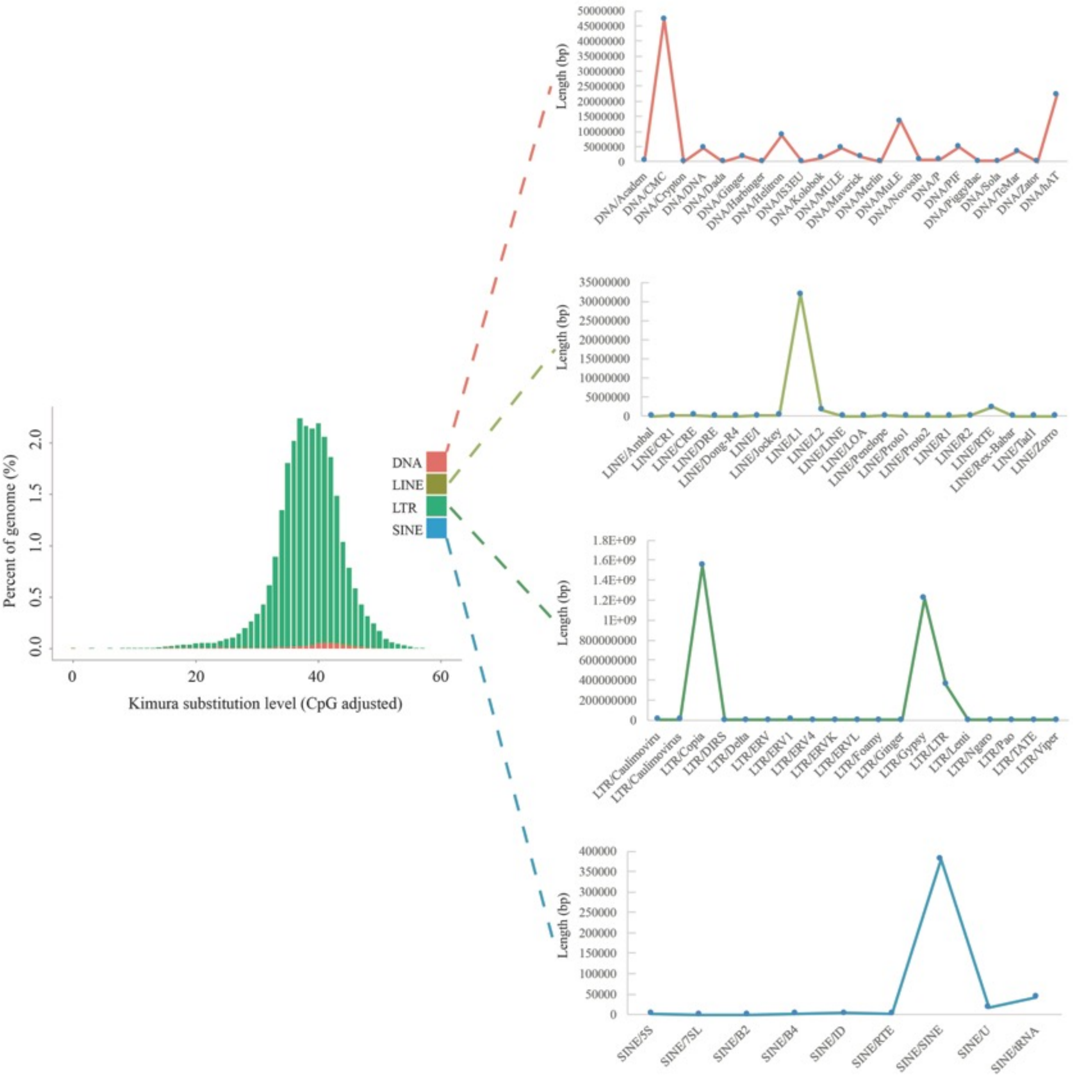

Supplementary Figure 2. The frequency and the length of the main different types of repetitive sequences in celery genome, including DNA, LINE, LTR, and SINE repeats.

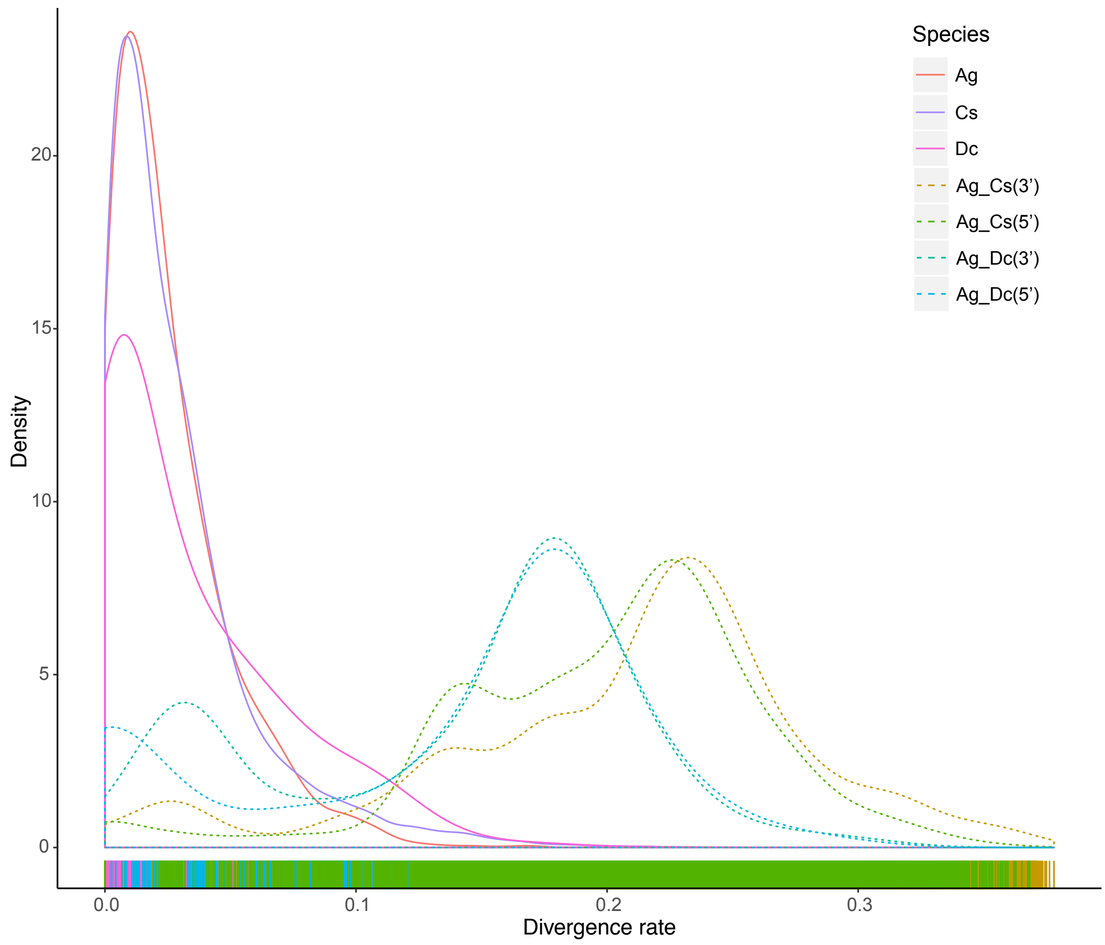

Supplementary Figure 3. The density of divergence rates within and among celery, coriander, and carrot LTR repeat sequences. The 3’ and 5’ represent the 3'- and 5'-ends of LTR, respectively.

a

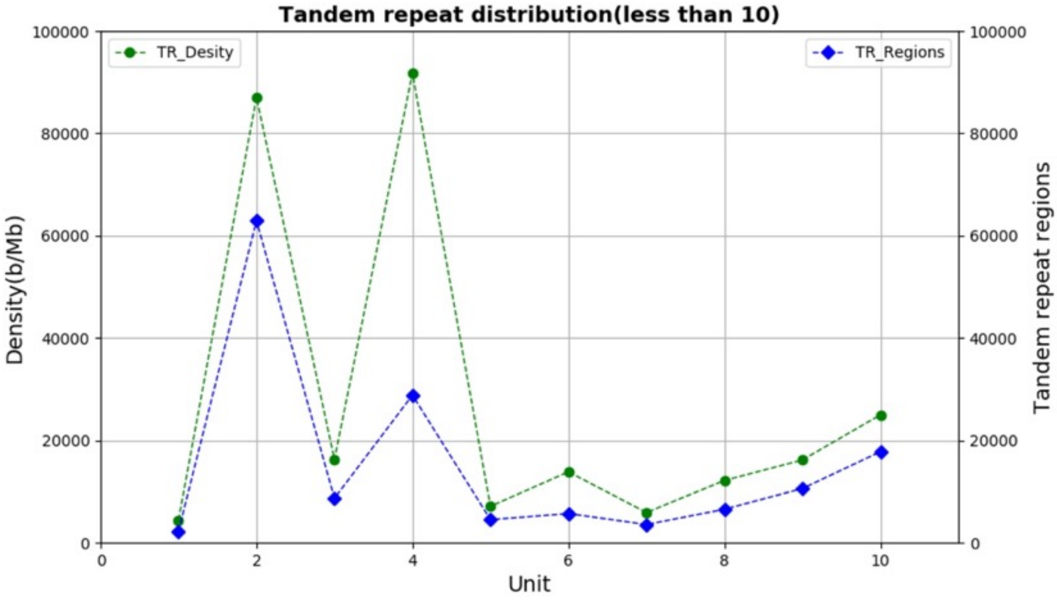

b

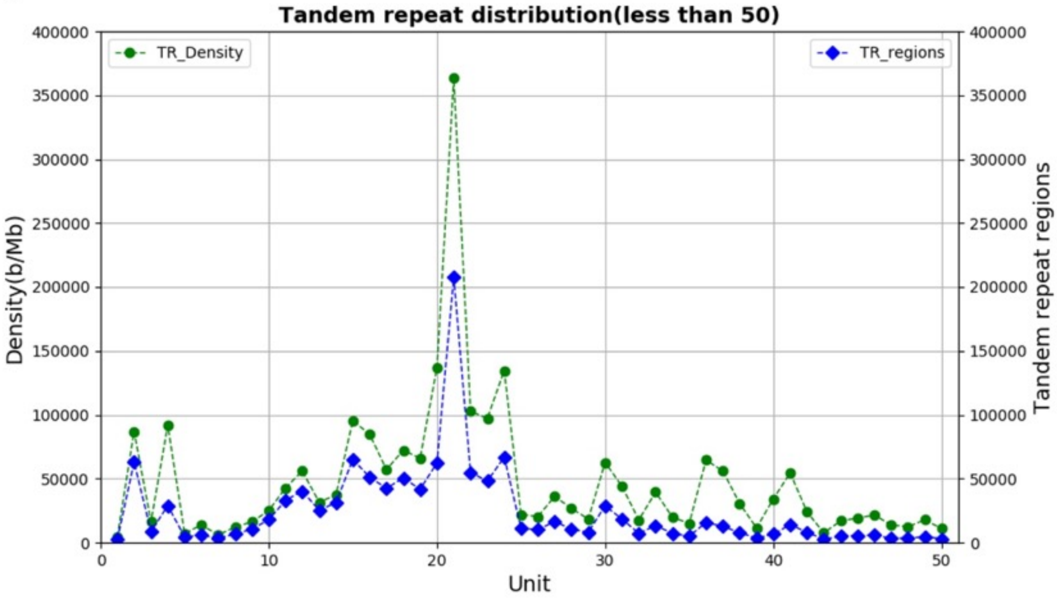

Supplementary Figure 4. Tandem repeat distribution in the celery genome. The green dot line indicates the tandems repeat density, and the blue dot line indicates the number of tandem repeat region. (a) the unit of the tandem repeat was =<10 bp. (b) the unit of the tandem repeat was =<50 bp.

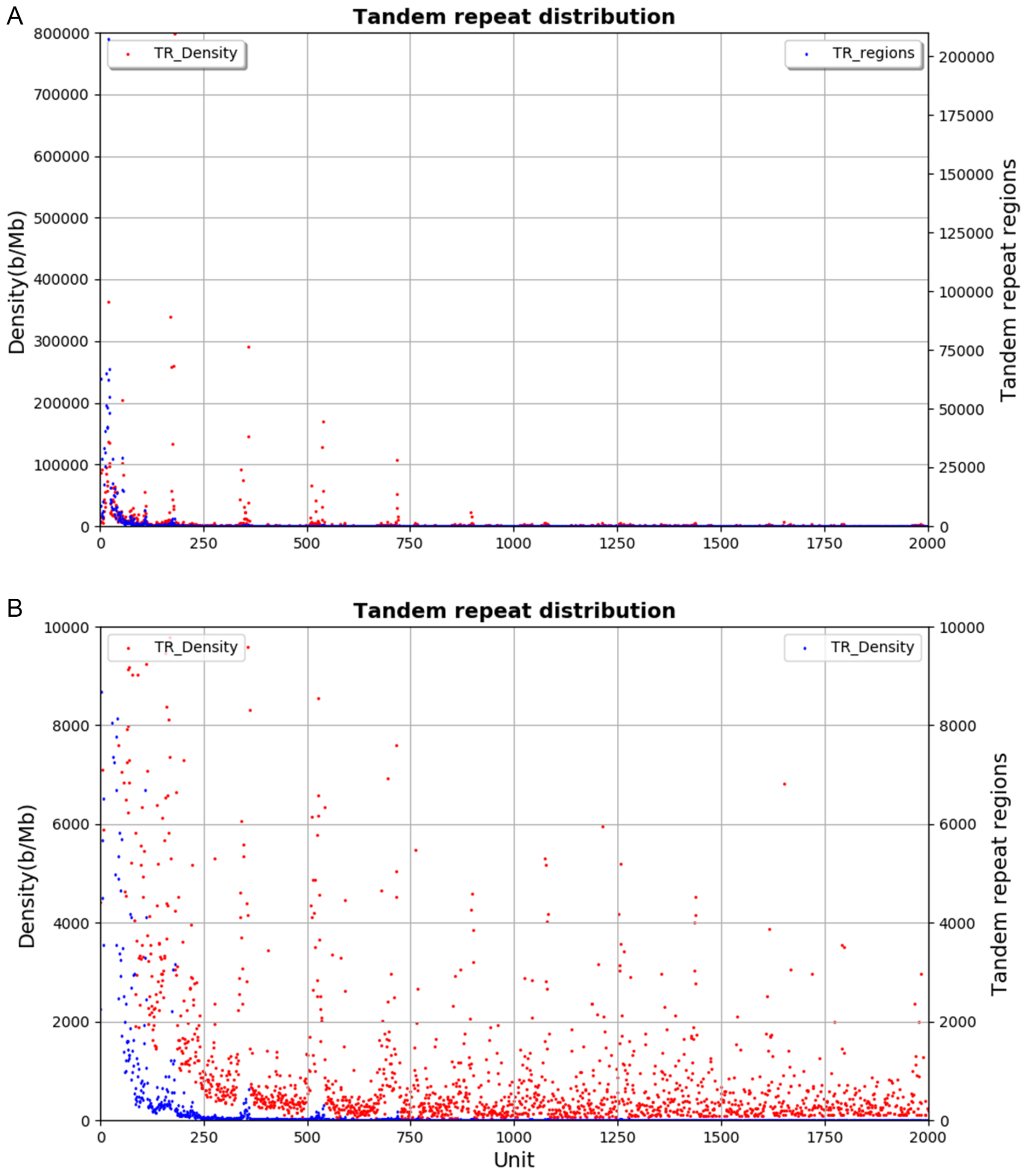

Supplementary Figure 5. Tandem repeat distribution in the celery genome. Red dots indicate the tandem repeat density, and blue dots indicate the number of tandem repeat region. (a) all the unit (0~800000) of the tandem repeat. (b) the enlargement of Y axis (0~10000) of the tandem repeat.

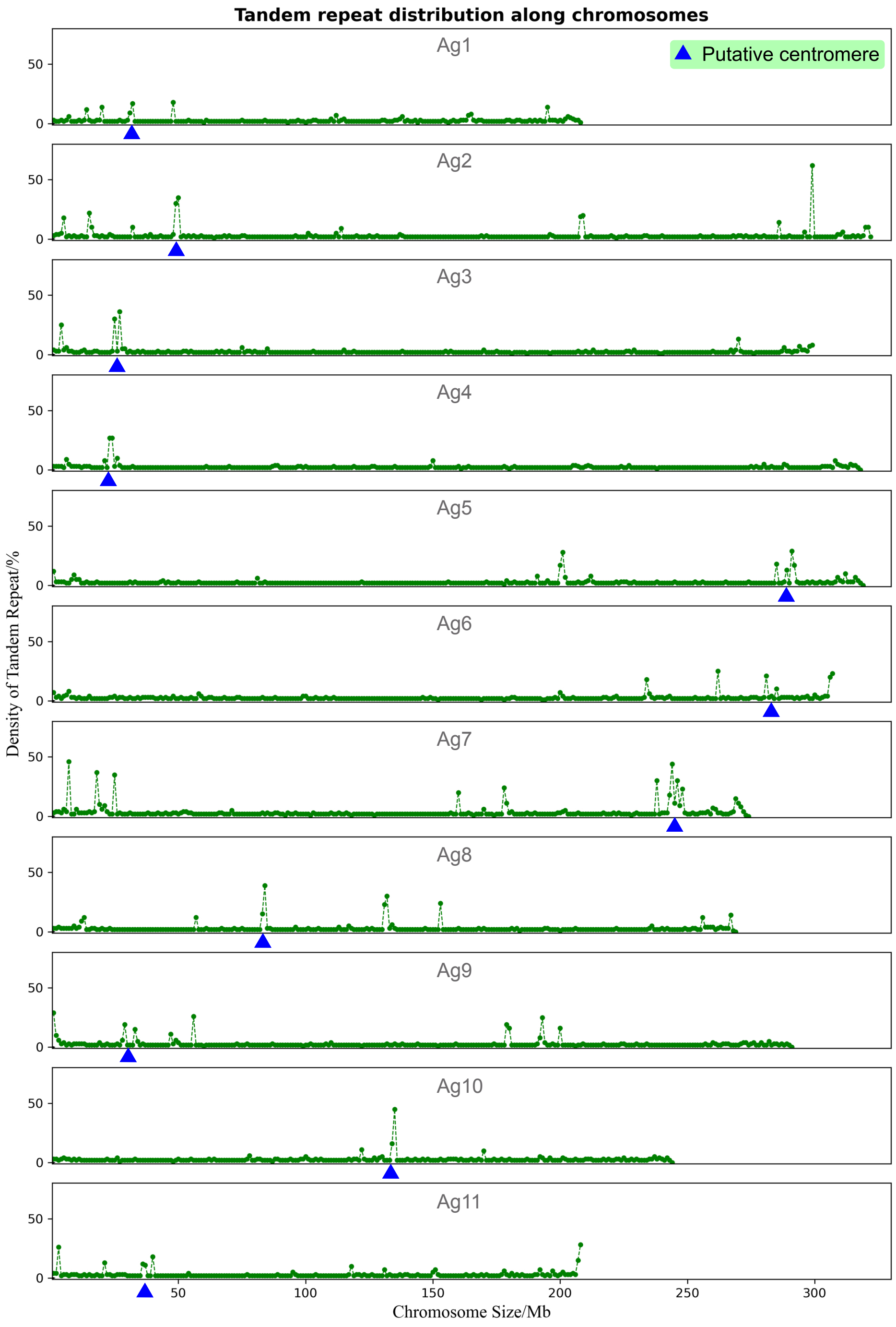

Supplementary Figure 6. The predication of putative centromere regions of each celery chromosome according the peak and length of tandem repeat density. The blue triangle indicates the putative centromere region of each chromosome. The x axis represented the length of chromosome with 1 Mb as unit, and the y axis showed the percentages of tandem repeats along 11 chromosomes, respectively.

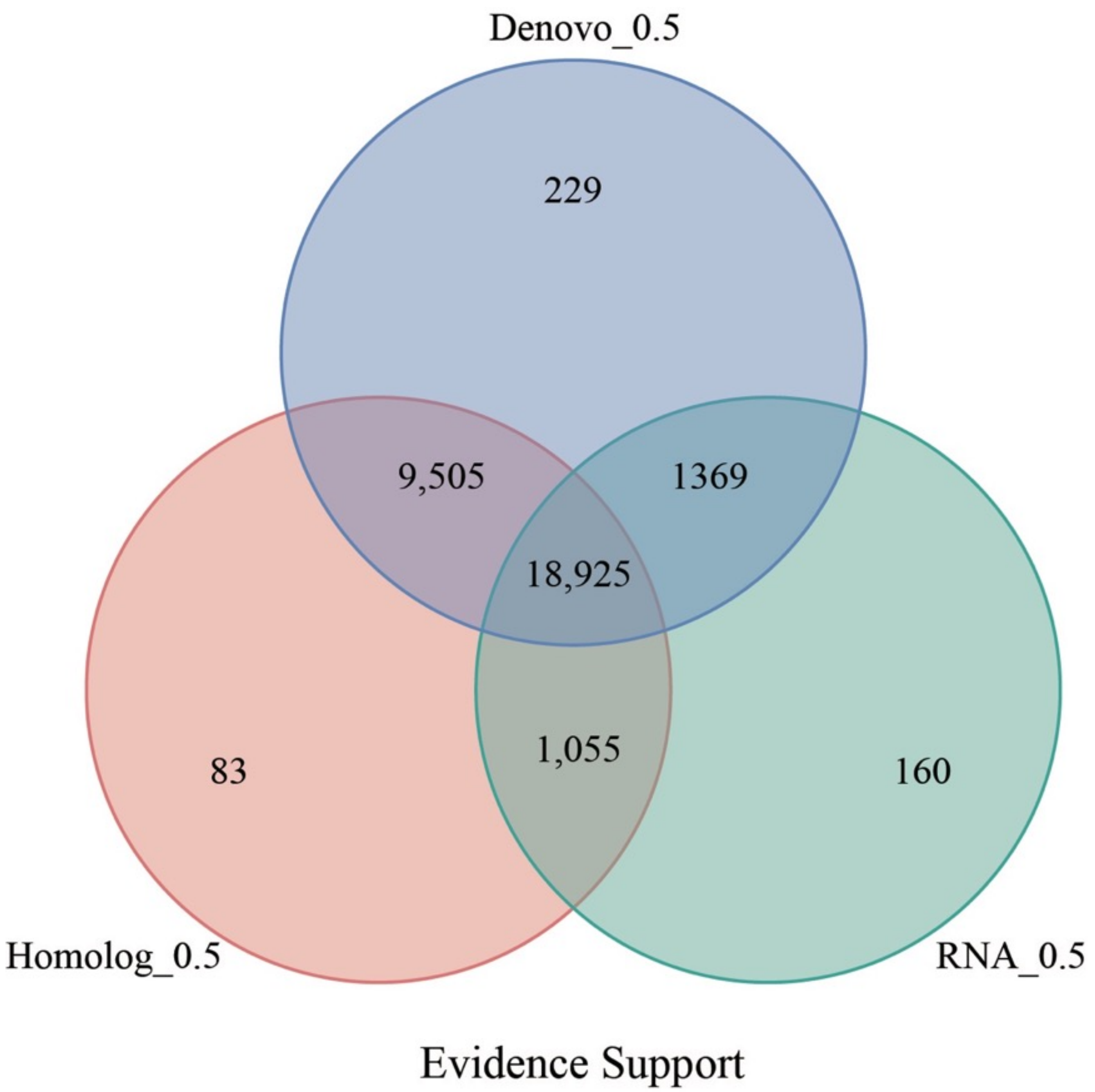

Supplementary Figure 7. The statistics of gene set evidence supports in celery genome. De novo_0.5, EVM integrates genes supported by *Denovo* prediction; Homolog_0.5, genes supported by homologous prediction when EVM integration; RNA_0.5, genes supported by RNA-seq during EVM integration. The gene overlap is greater than 50% as a standard, and the number indicates the number of genes.

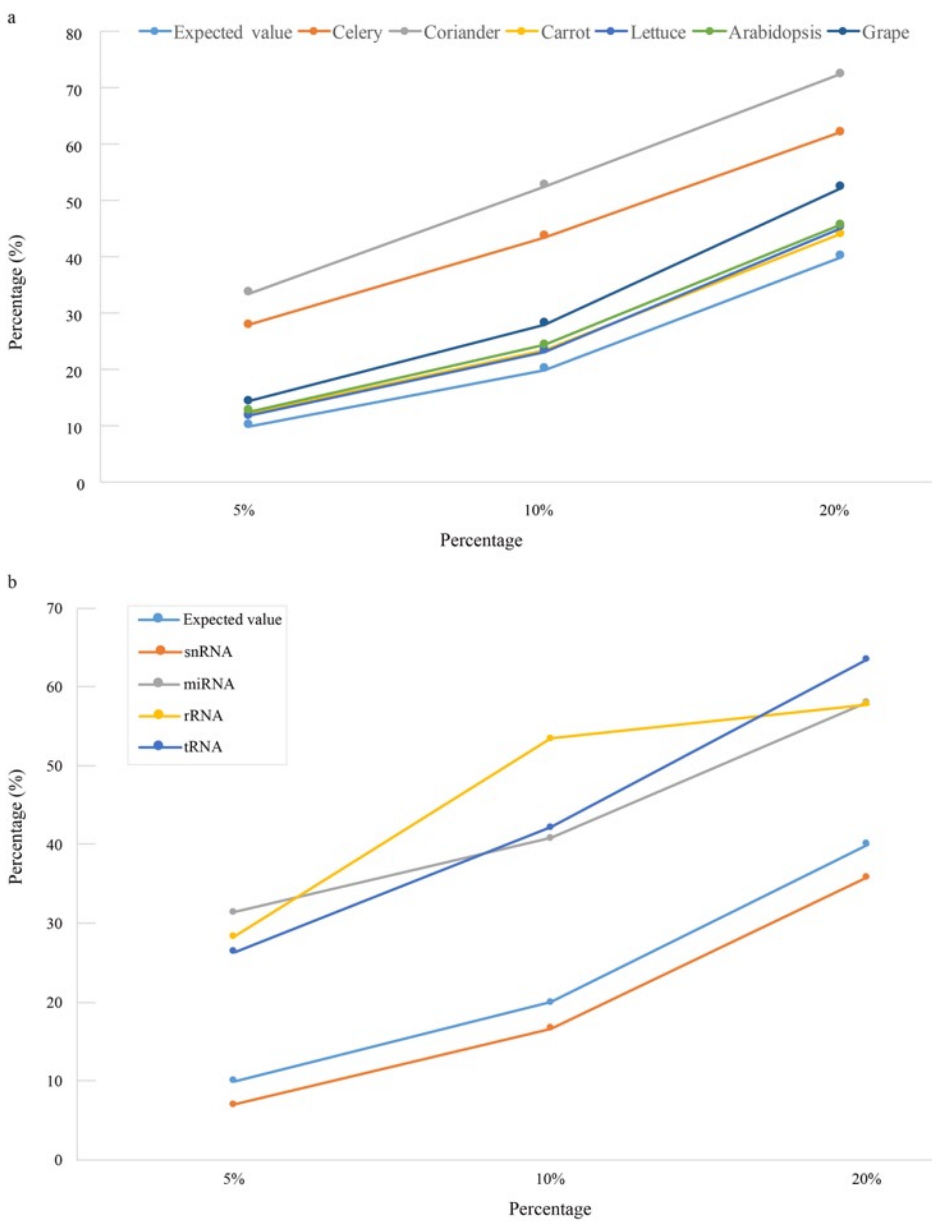

Supplementary Figure 8. (a) The statistics of gene distribution on each chromosome of celery, and other 5 species. *%5 means that we cut the 5% of the full length of the chromosome at two sides of the chromosome, and then we statistic the distribution density of gene in the two intervals. (b) The statistics of snRNA, miRNA, rRNA, and tRNA distribution on each celery chromosome. *%5 means that we cut the 5% of the full length of the chromosome at two sides of the chromosome, and then we statistic the distribution density of each type of ncRNA in the two intervals.

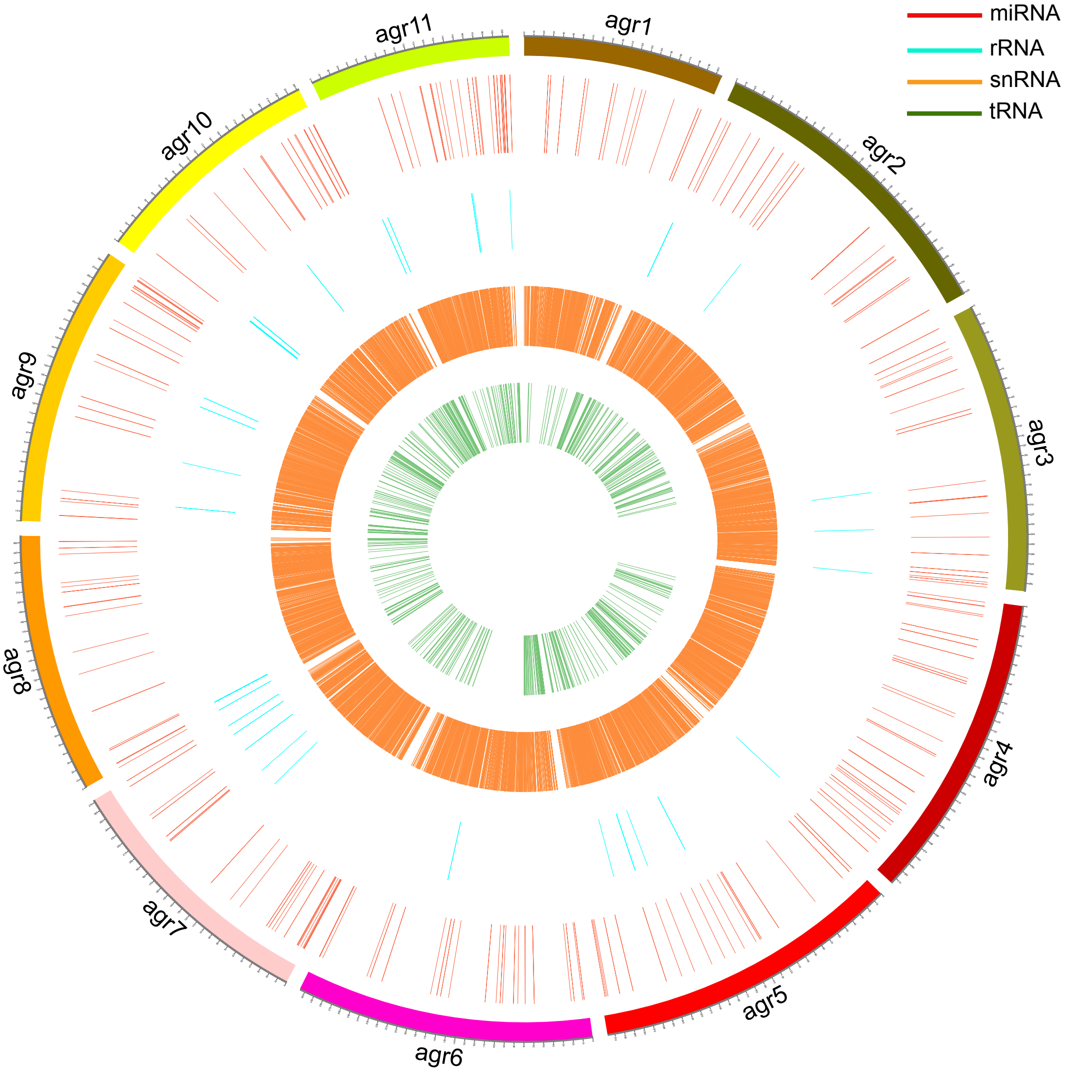

Supplementary Figure 9. The chromosomal distribution of ncRNA of celery. miRNA, rRNA, snRNA, and tRNA are displayed in circles from outsides to insides, respectively.

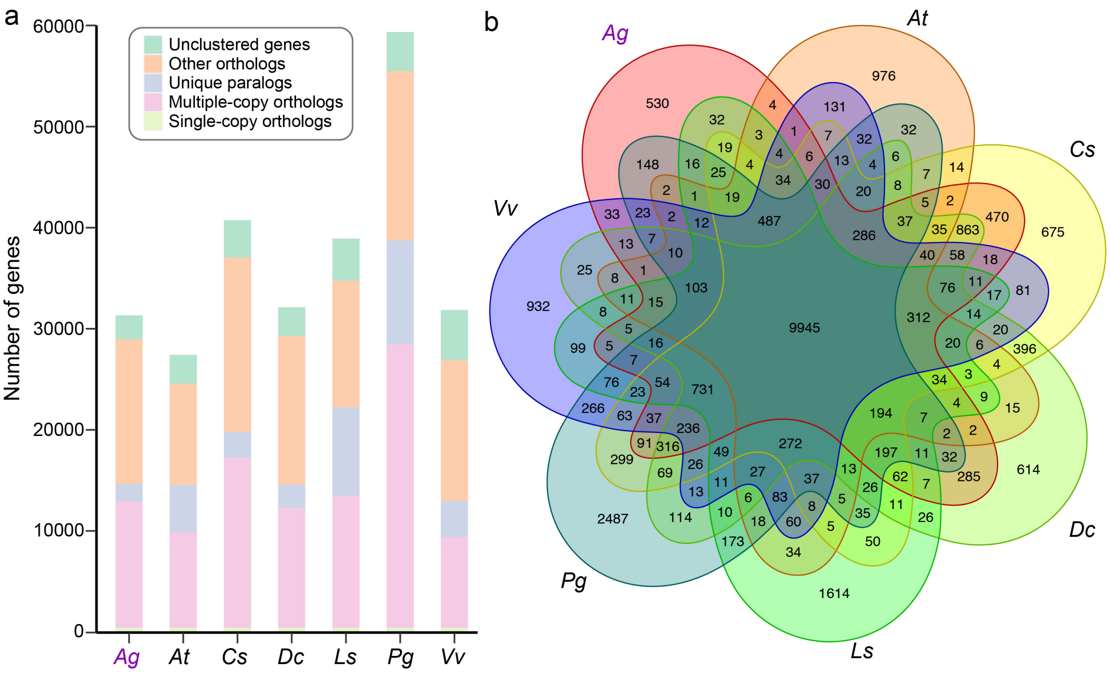

Supplementary Figure 10. Gene family analyses. (a) The distribution of gene numbers and family sizes in celery (Ag: *A. graveolens*) and 6 other representative species (At: *Arabidopsis thaliana*; Cs: *Coriandrum sativum*; Dc: *Daucus carota*; Ls: *Lactuca sativa*; Vv: *Vitis vinifera*, Pg: *Panax ginseng*). (b) Common and lineage-specific gene families in celery and 6 species.

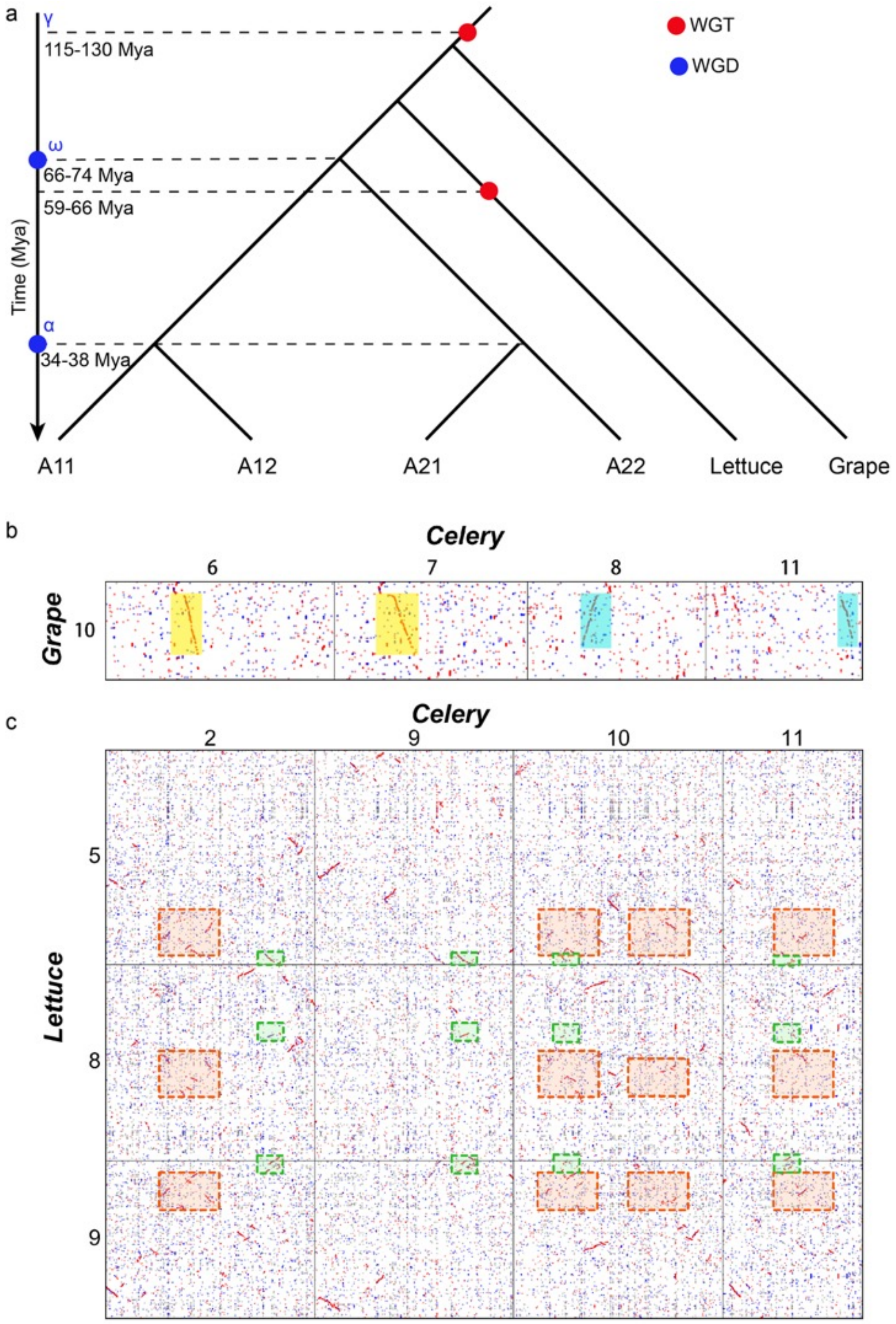

Supplementary Figure 11. The phylogenetic tree of grape and Apiaceae orthologous genes. (a) γ denoted by red circle, the α and ω events denoted by blue circle. A grape gene has four orthologs in the Apiaceae species genome, and named as A11, A12, A21, and A22. The species tree was produced based on our present analysis of homologous genes. (b) Homologous gene dotplot between grape and celery shows an orthologous gene ratio 1:4. (c) Homologous gene dotplot between lettuce and celery shows an orthologous gene ratio 3:4.

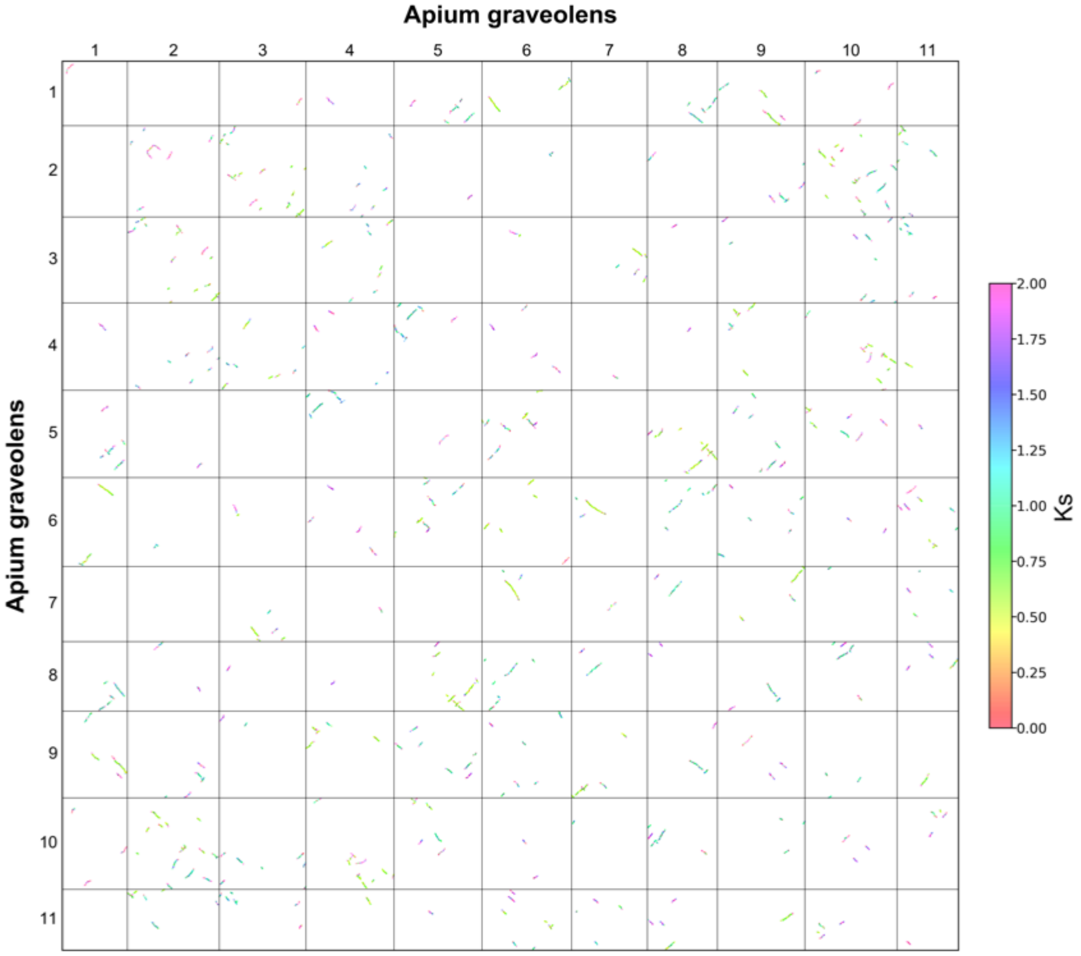

Supplementary Figure 12. (a) Homologous dotplot within celery genome. Median Ks value for duplicated genes on each inferred colinear block is shown, in color from red to purple as to the value of Ks

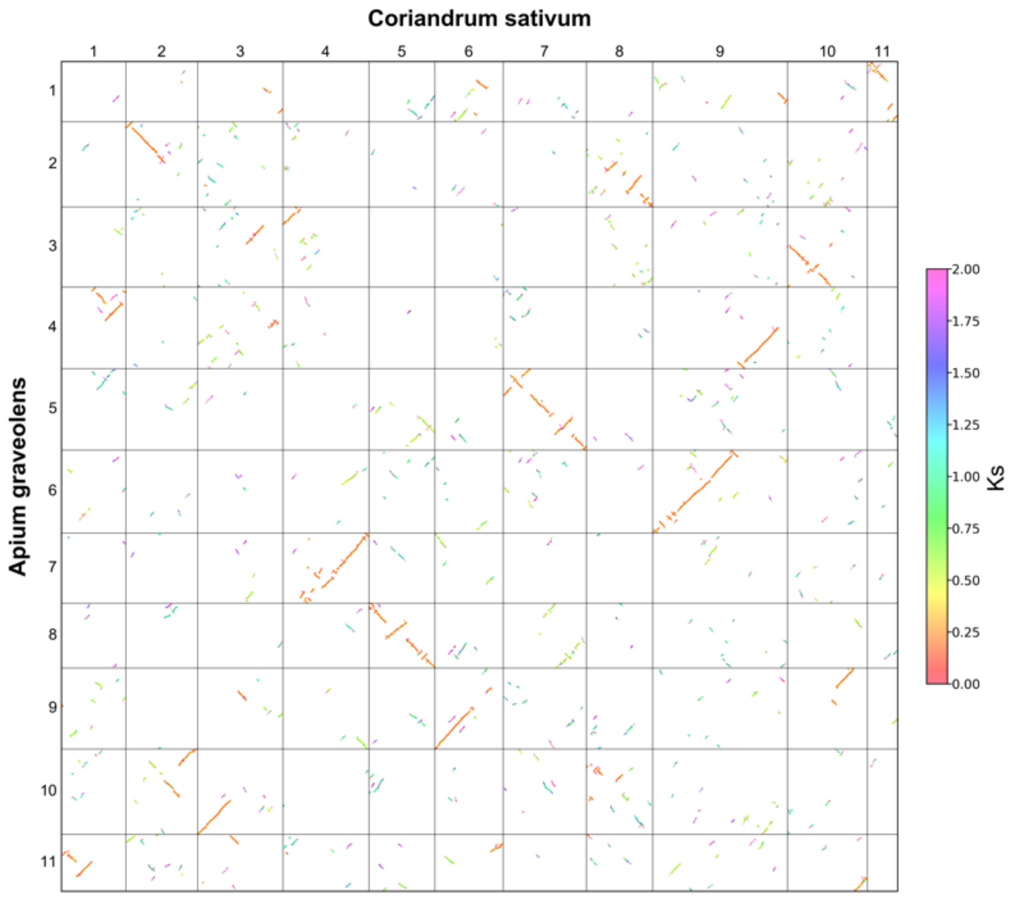

Supplementary Figure 12. (b) Homologous dotplot between celery and coriander genomes. Median Ks value for duplicated genes on each inferred colinear block is shown, in color from red to purple as to the value of Ks.

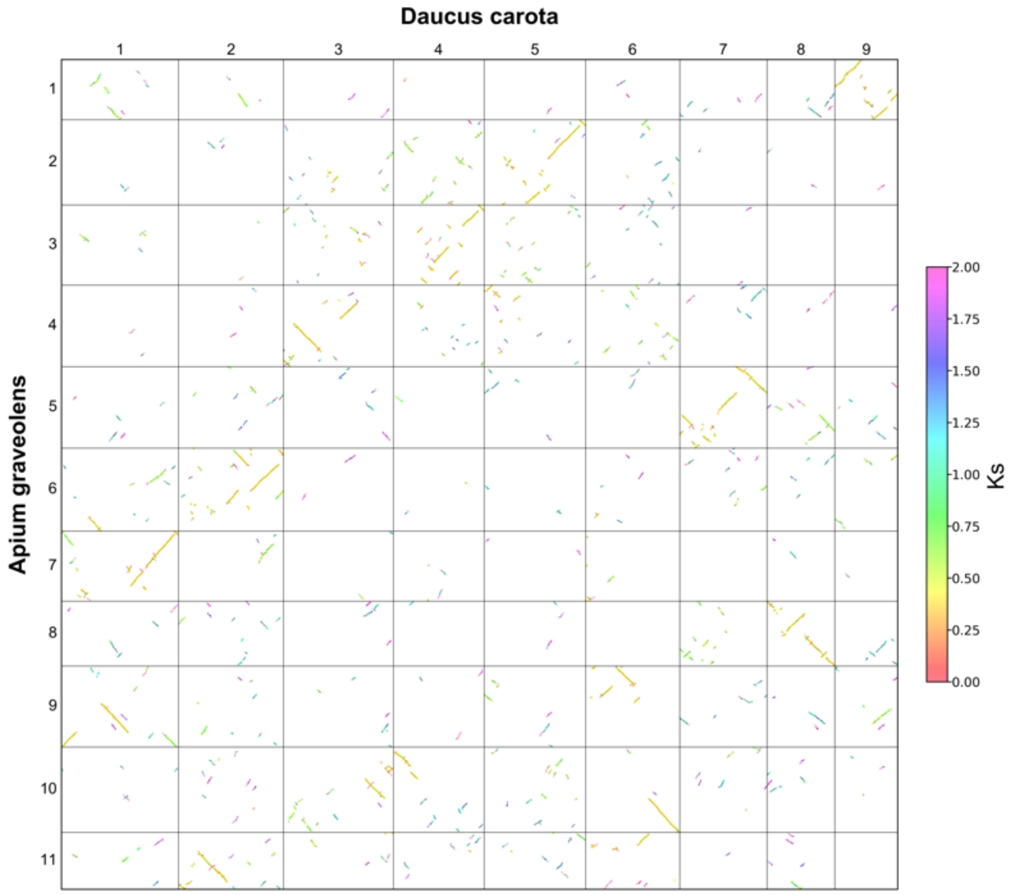

Supplementary Figure 12. (c) Homologous dotplot between celery and carrot genomes. Median Ks value for duplicated genes on each inferred colinear block is shown, in color from red to purple as to the value of Ks.

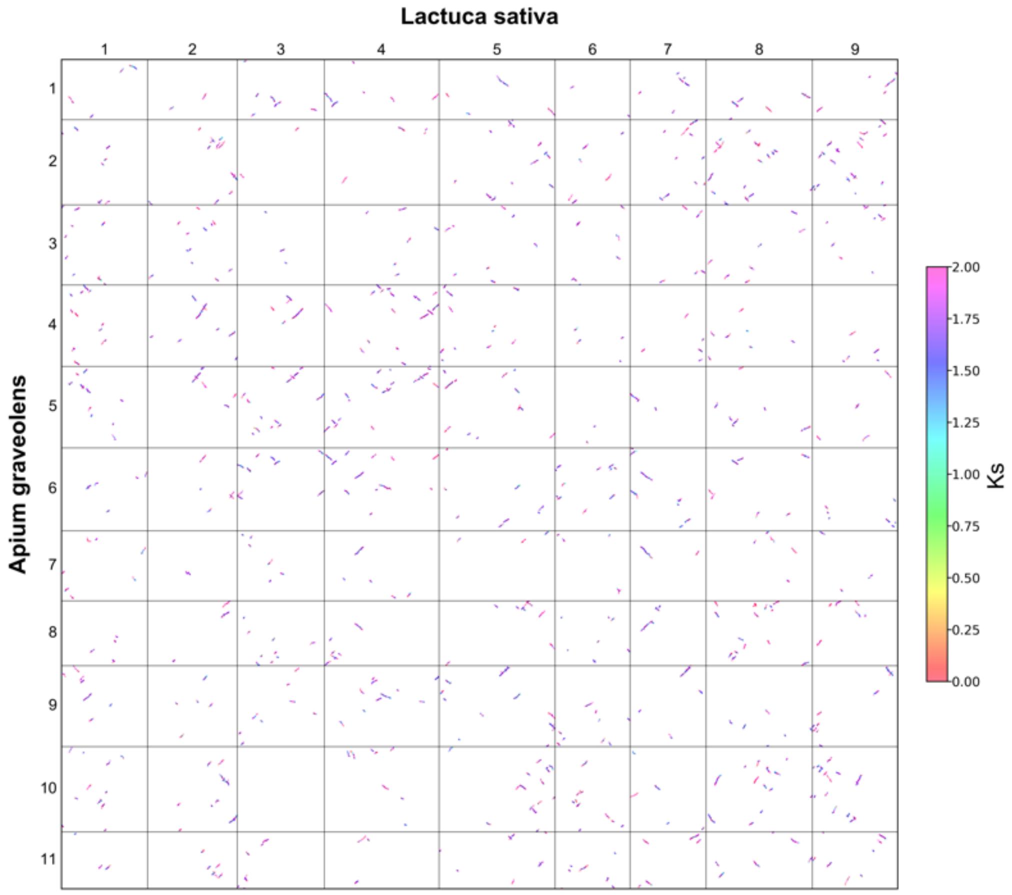

Supplementary Figure 12. (d) Homologous dotplot between celery and lettuce genomes. Median Ks value for duplicated genes on each inferred colinear block is shown, in color from red to purple as to the value of Ks.

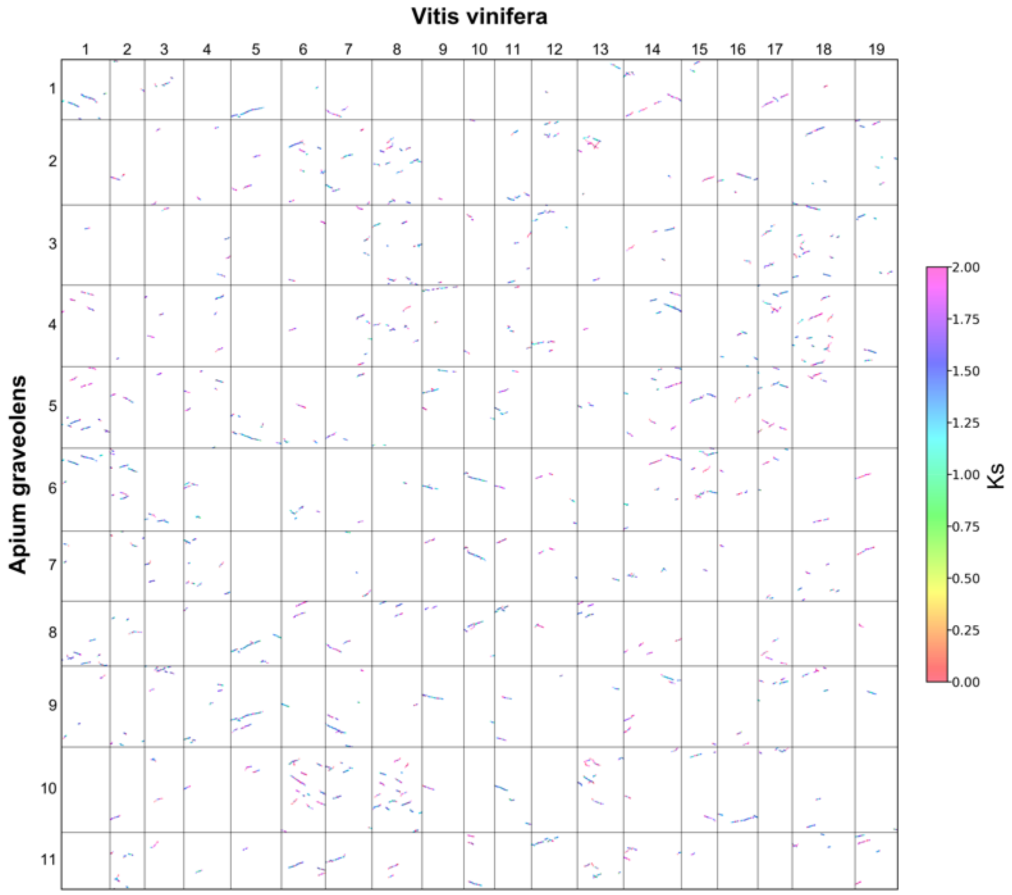

Supplementary Figure 12. (e) Homologous dotplot between celery and grape genomes. Median Ks value for duplicated genes on each inferred colinear block is shown, in color from red to purple as to the value of Ks.

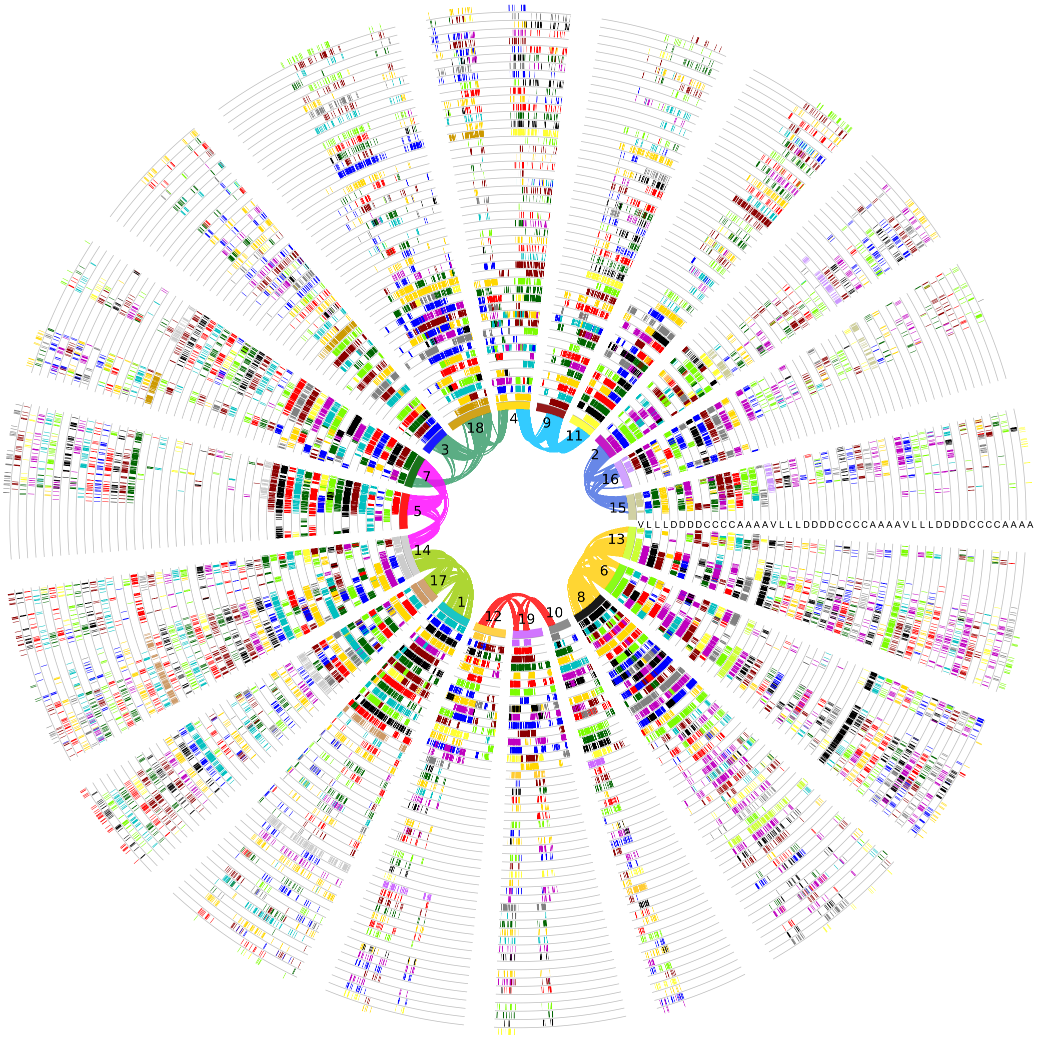

Supplementary Figure 13. Homologous alignment of Apiaceae and other relative genomes with grape as a reference. Here, we show global alignment of homologous regions in lettuce (L), carrot (D), coriander (C), celery (A) genomes and grape (V). Genomic paralogy and orthology information within and among genomes, is displayed with 48 circles, each corresponding to (sub)genomes produced by recursive polyploidizatioin. The curved lines within the innermost circle show paralogous genes among 19 grape chromosomes, in 7 colors corresponding to the 7 ancestral chromosomes before the WGT (γ). The short lines forming the innermost grape chromosome circles represent grape genes. Each of the three sets of grape paralogous regions has 3 orthologous regions in lettuce, and 4 in celery, coriander and carrot, respectively. This results in 48 circles marked according to species indicated by a capital letter.

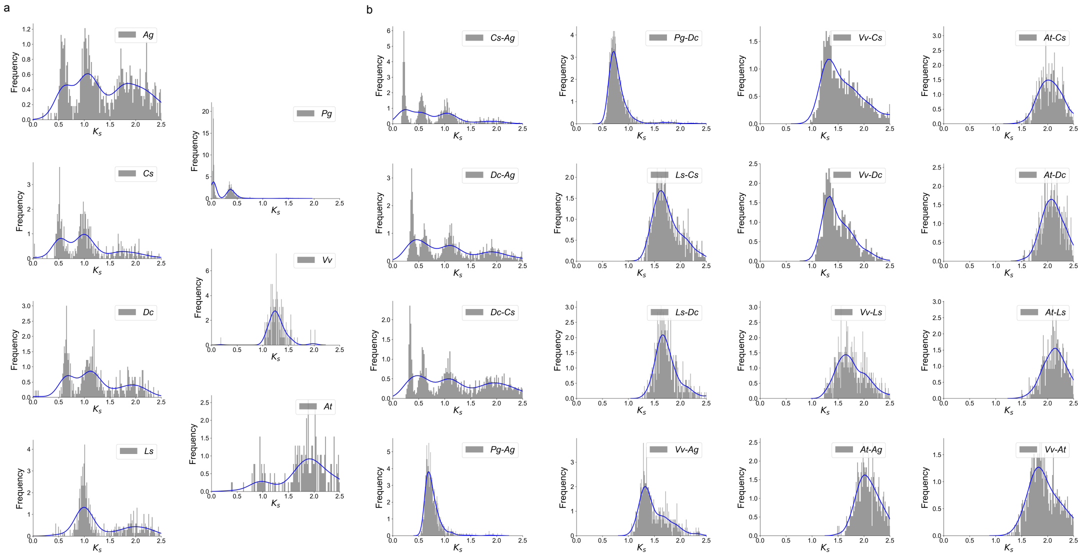

Supplementary Figure 14. Histograms and fitted curves of Ks between colinear homologous genes. The median Ks value of each homologous block is used. (a) intragenomic; (b) intergenomic.

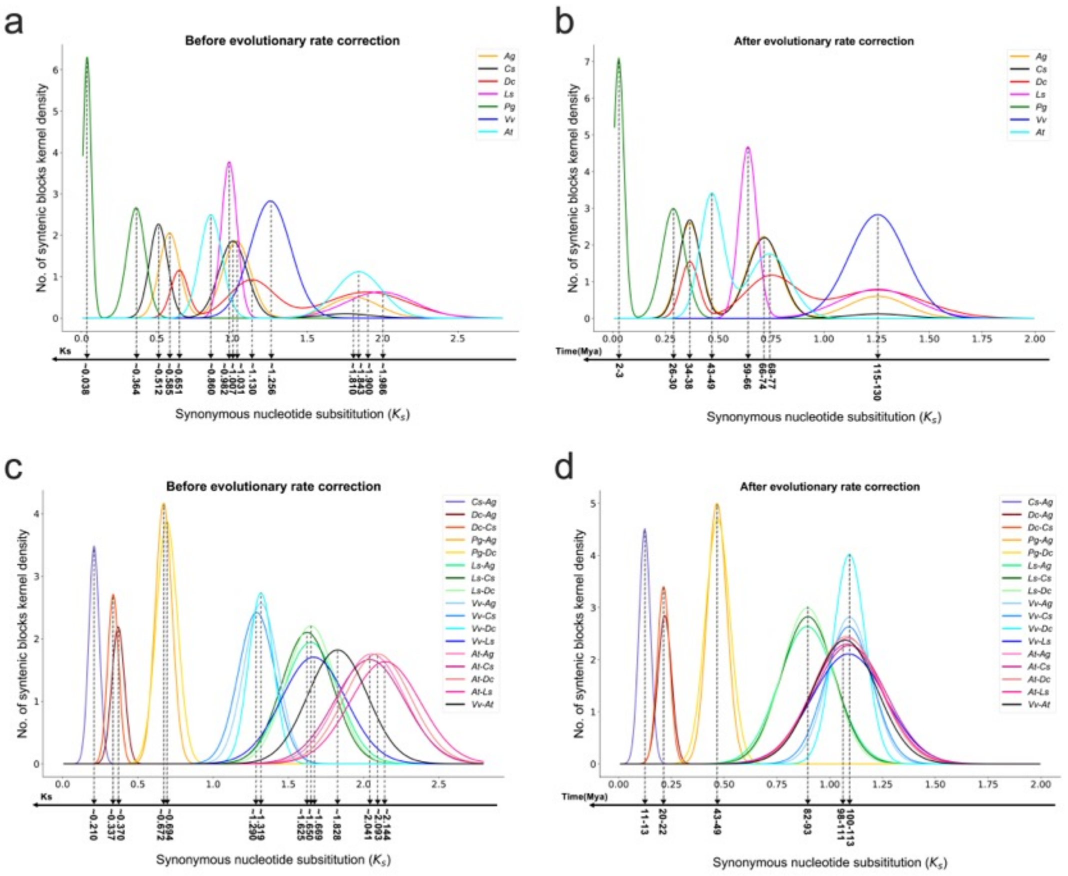

Supplementary Figure 15. The original and corrected synonymous nucleotide substitutions (Ks), and inferred evolutionary dates among colinear genes. (a) Ks distribution and inferred evolutionary dates within a species before evolutionary rate correction (representing the polyploidization events). (b) Ks distribution and inferred evolutionary dates within a species after evolutionary rate correction (representing the polyploidization events). (c) Ks distribution and inferred evolutionary dates between any two species before evolutionary rate correction (representing the speciation events). (d) Ks distribution and inferred evolutionary dates between any two species after evolutionary rate correction (representing the speciation events).

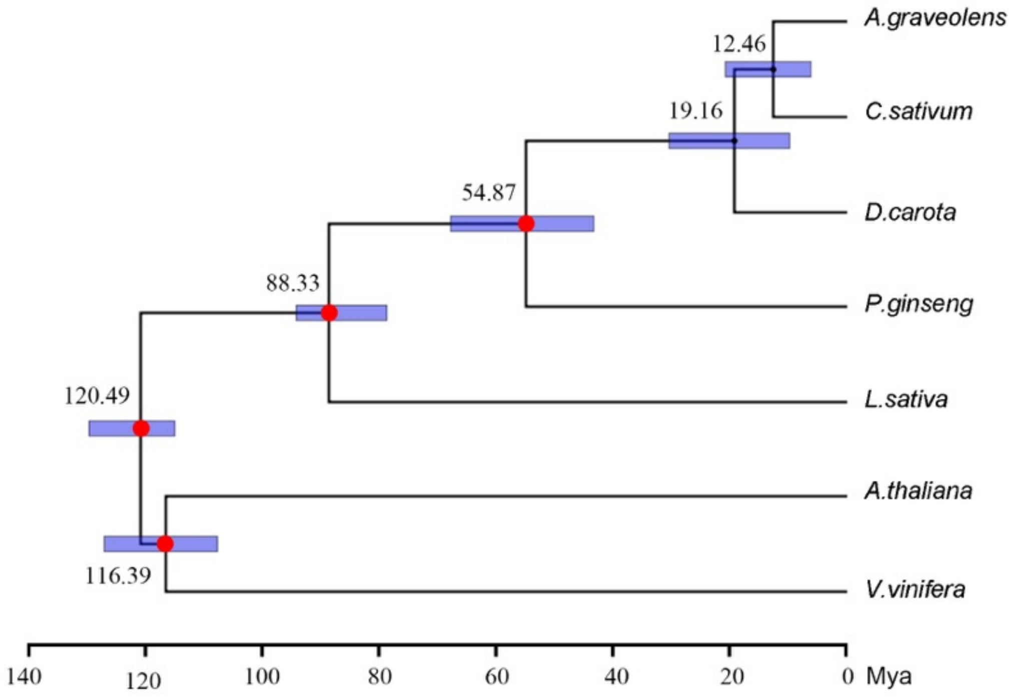

Supplementary Figure 16. Divergence time estimated among 7 species. The tree topology was generated by MCMCtree and the CDS sequences. The red dots correspond to calibration points as mentioned in the method. Divergence estimates (Mya, million years ago) are indicated above each node and the blue nodal bars show the 95% confidence intervals (n=20,000 independent samples).

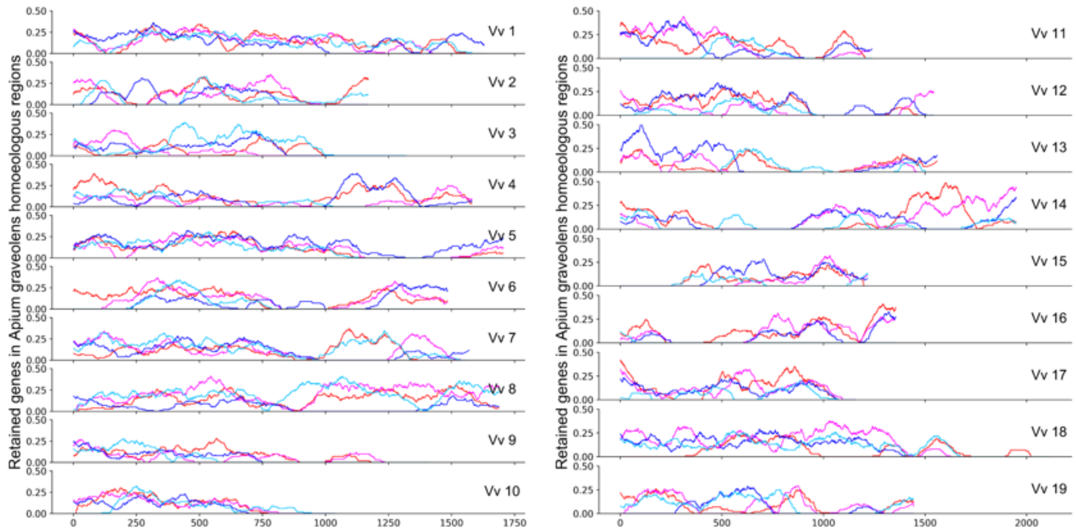

Supplementary Figure 17. (a) The retention of duplicated genes residing in 4 subgenomes of celery using the grape as reference.

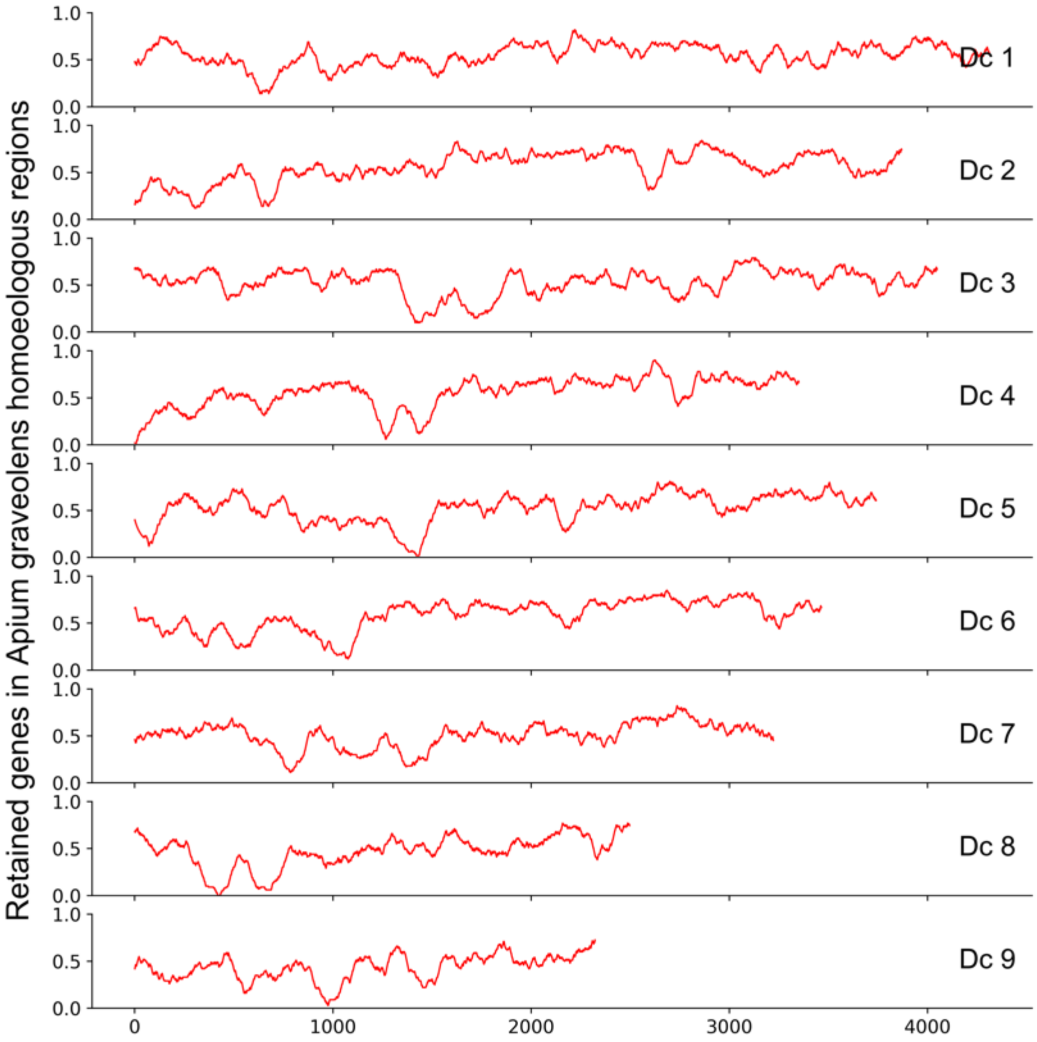

Supplementary Figure 17. (b) The retention of duplicated genes residing in celery subgenomes using the carrot as reference.

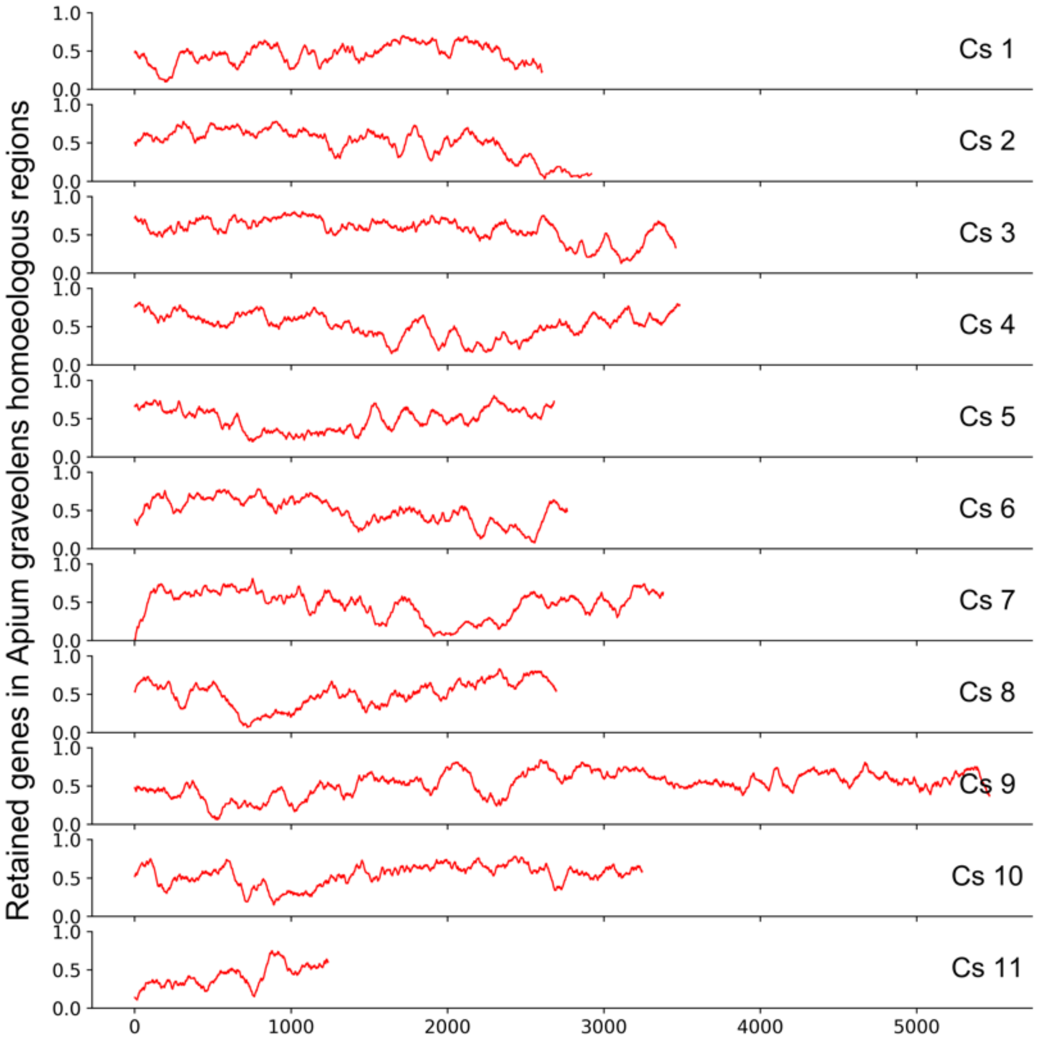

Supplementary Figure 17. (c) The retention of duplicated genes residing in celery genome using the coriander as reference.

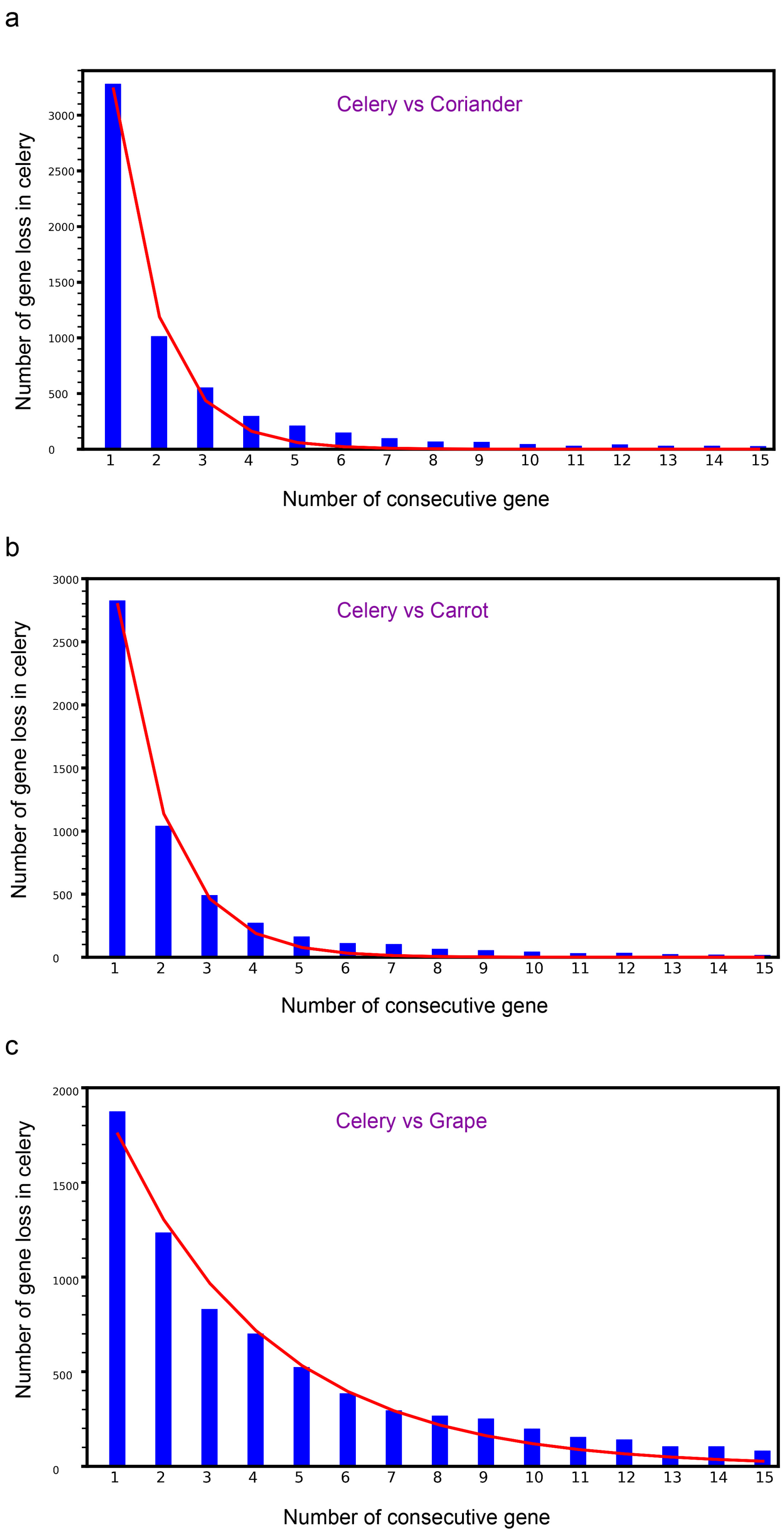

Supplementary Figure 18. Fitting a geometric distribution to gene loss frequencies in celery as to the coriander (a), carrot (b), and grape (c), respectively.

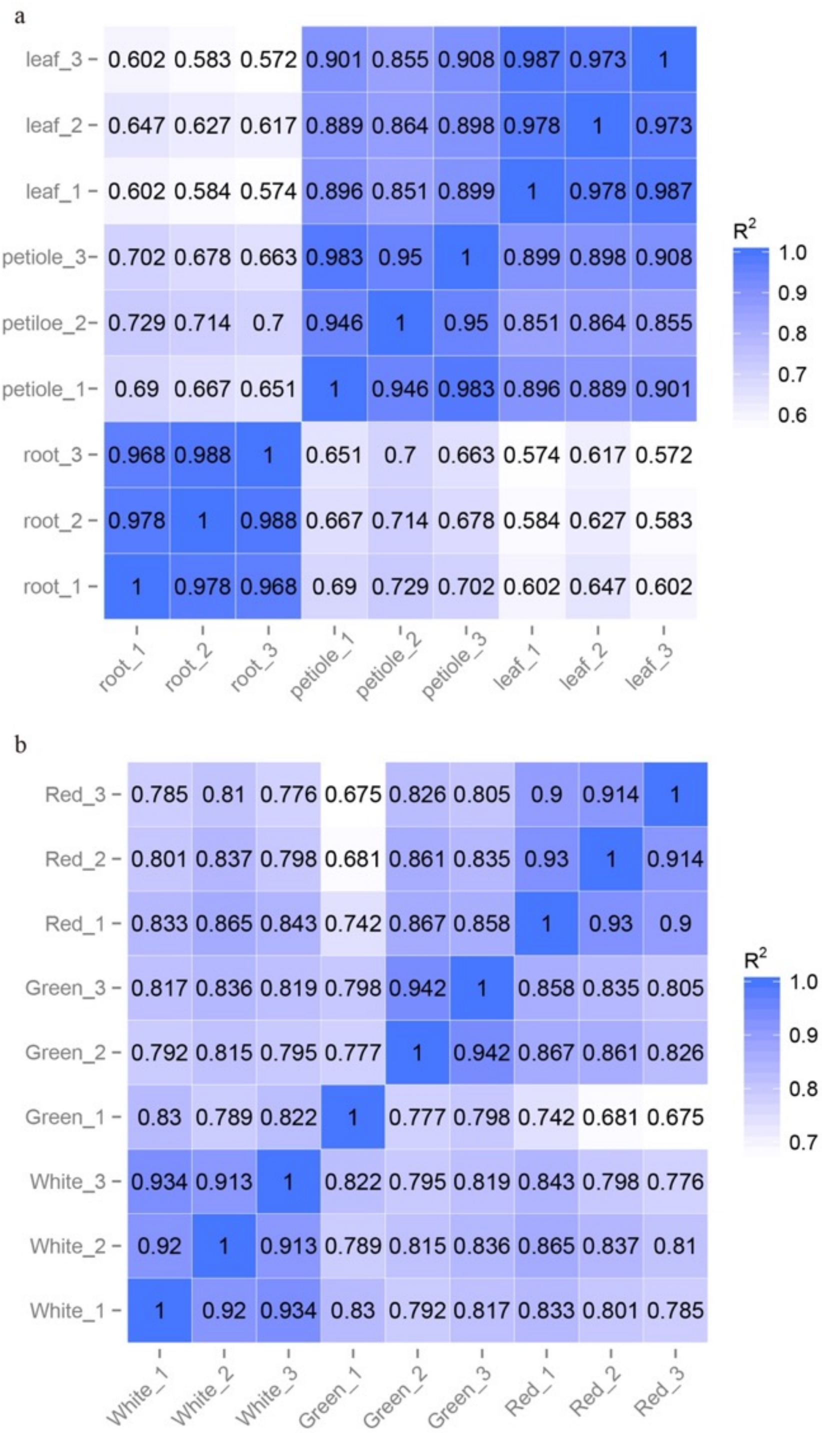

Supplementary Figure 19. The heat map of correlation coefficients was shown between any two of samples by RNA-Seq. (a) The RNA-Seq for three tissues (root, petiole, leaf) of celery. (b) The RNA-Seq for 3 different-colored sample (Green, Red, and White) of celery.

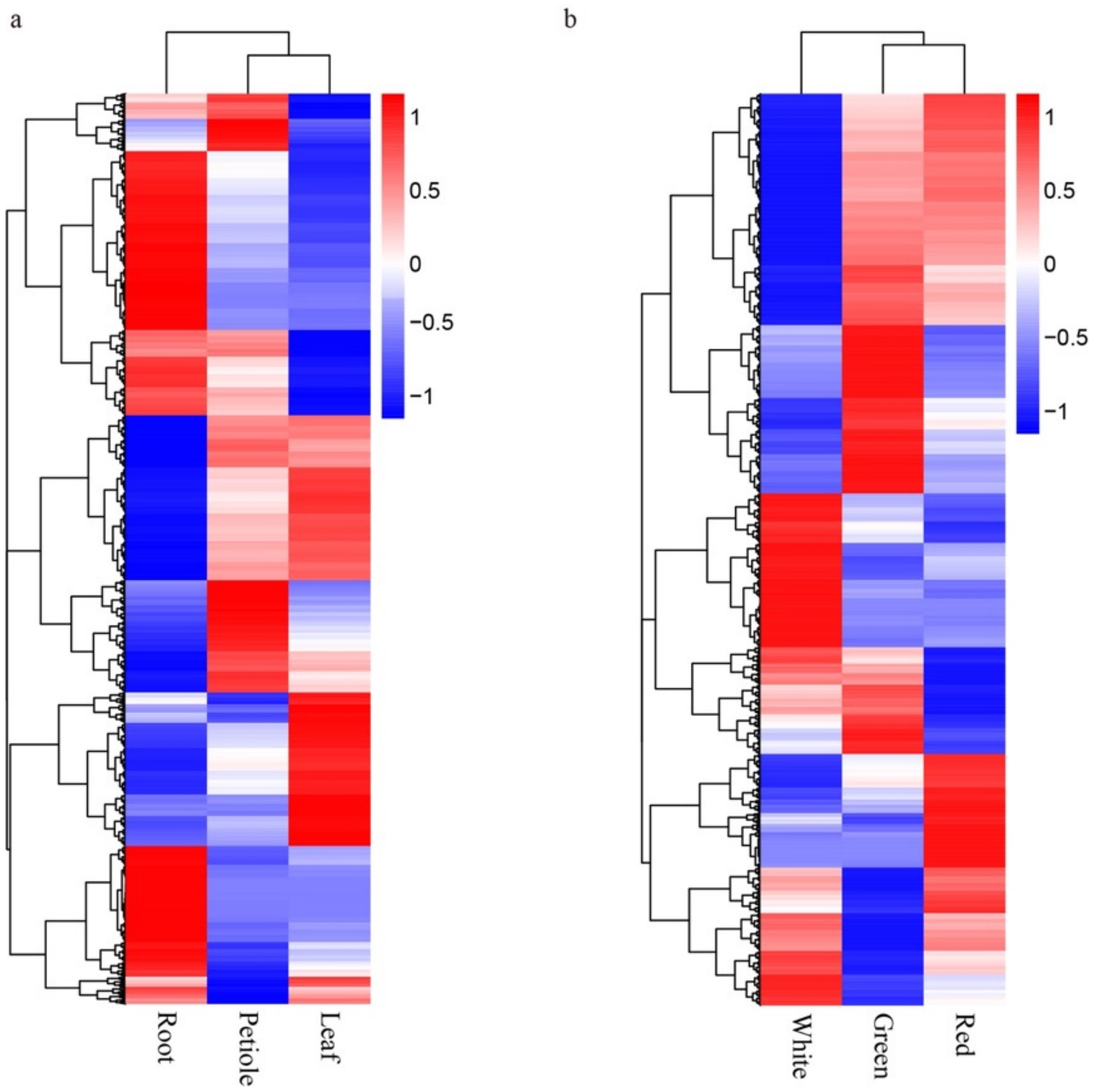

Supplementary Figure 20. Gene expression in different tissues and different celery varieties with different stem colors. The whole FPKM hierarchical clustering map, log10 (FPKM+1) values are normalized (scale number) and clustered, red indicates high expression gene, blue indicates low expression gene. The color ranges from red to blue, indicating log10 (FPKM+1) from large to small. (a) RNA-Seq analysis for three tissues (root, petiole, leaf) of celery. (b) RNA-Seq analysis for three different colors sample (green, red, and white) of celery.

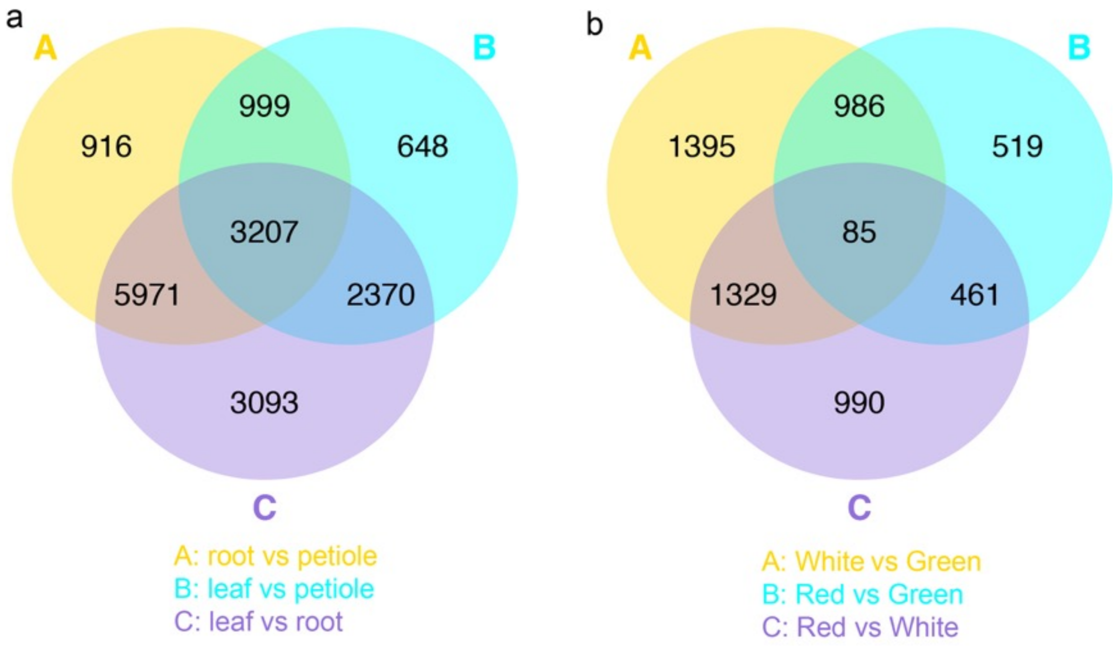

Supplementary Figure 21. Venn diagrams showed the common or specific DEGs among (a) different tissues or (b) different celery varieties with different stem colors.

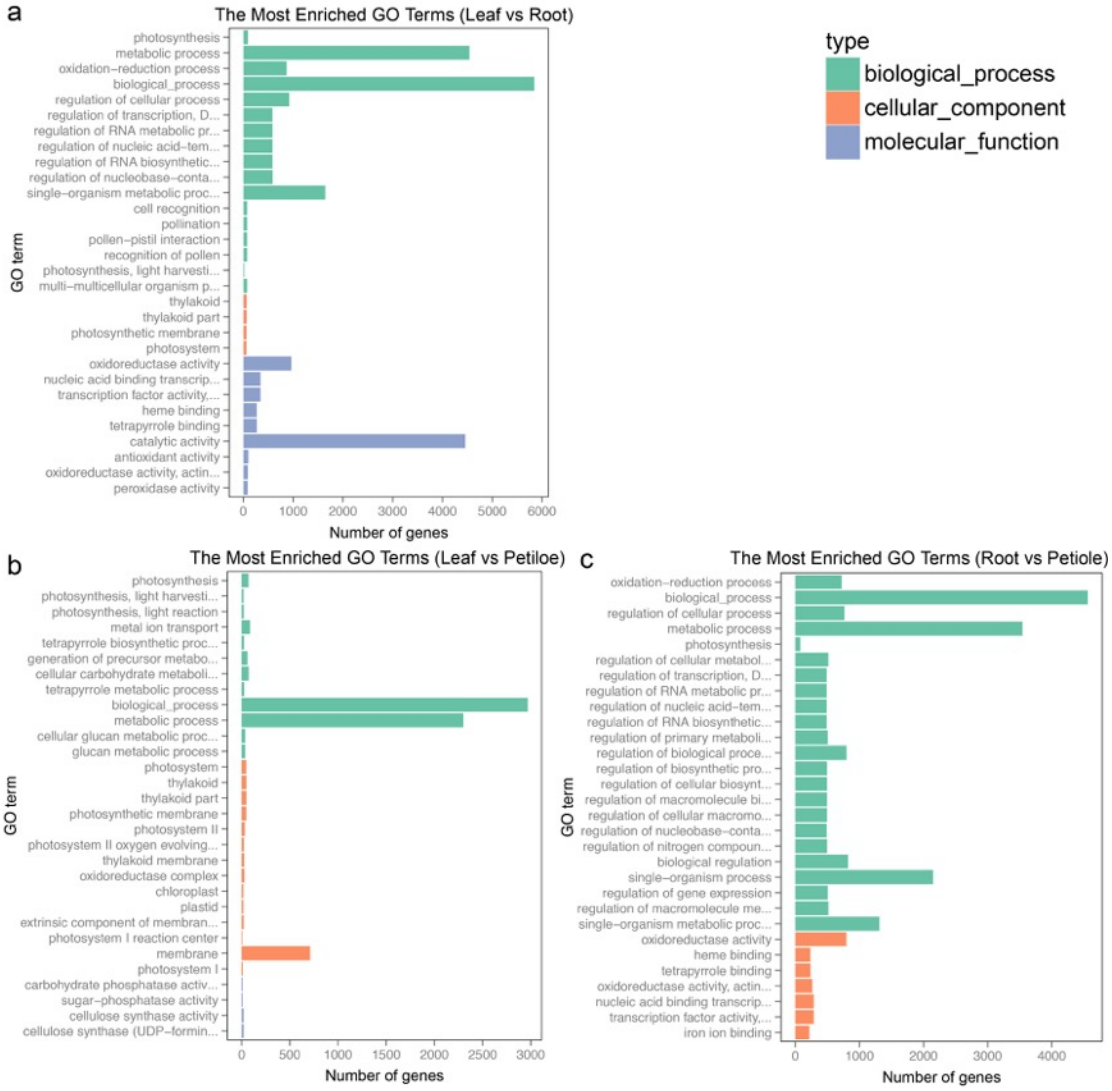

Supplementary Figure 22. The GO enrichment analyses of DEGs between any two tissues of celery with corrected P-value <0.05. (a) Leaf vs Root; (b) Leaf vs Petiole; (c) Root vs Petiole.

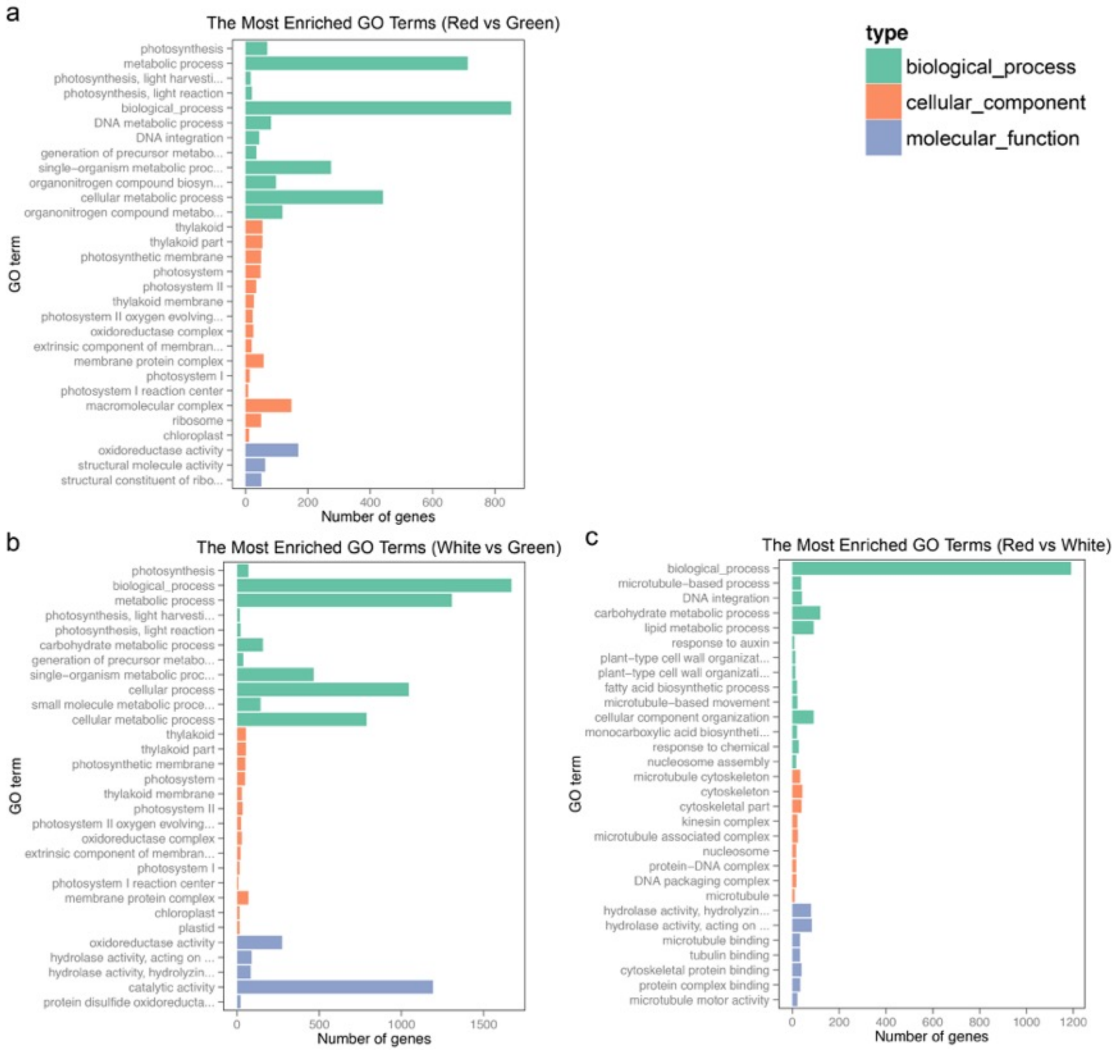

Supplementary Figure 23. The GO enrichment analyses of DEGs between any two varieties of celery with corrected *P*-value <0.05. (a) Red vs Green; (b) White vs Green; (c) Red vs White.

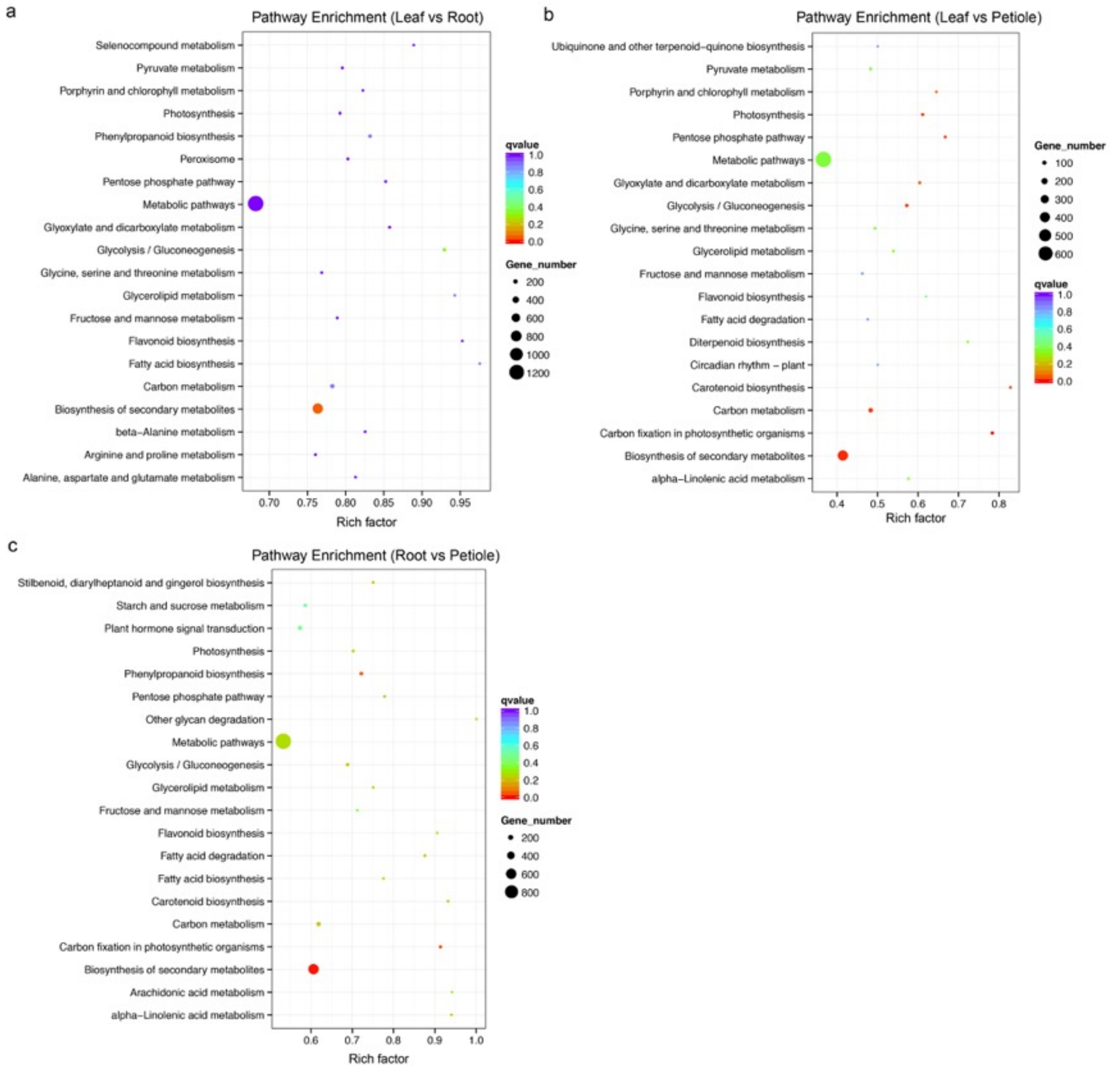

Supplementary Figure 24. The KEGG enrichment analyses of DEGs between any two tissues of celery. (a) Leaf vs Root; (b) Leaf vs Petiole; (c) Root vs Petiole.

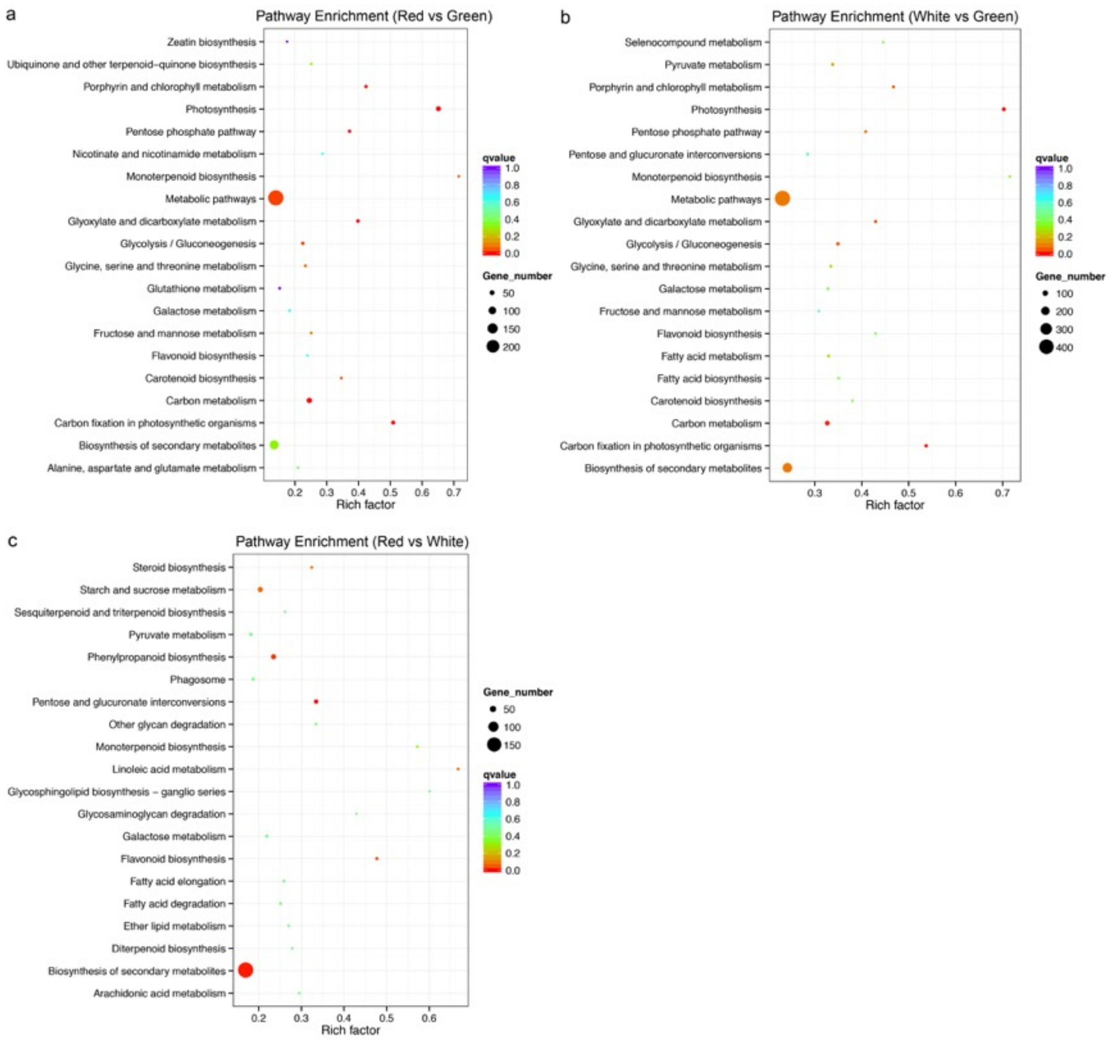

Supplementary Figure 25. The KEGG enrichment analyses of DEGs between any two varieties of celery. (a) Red vs Green; (b) White vs Green; (c) Red vs White.

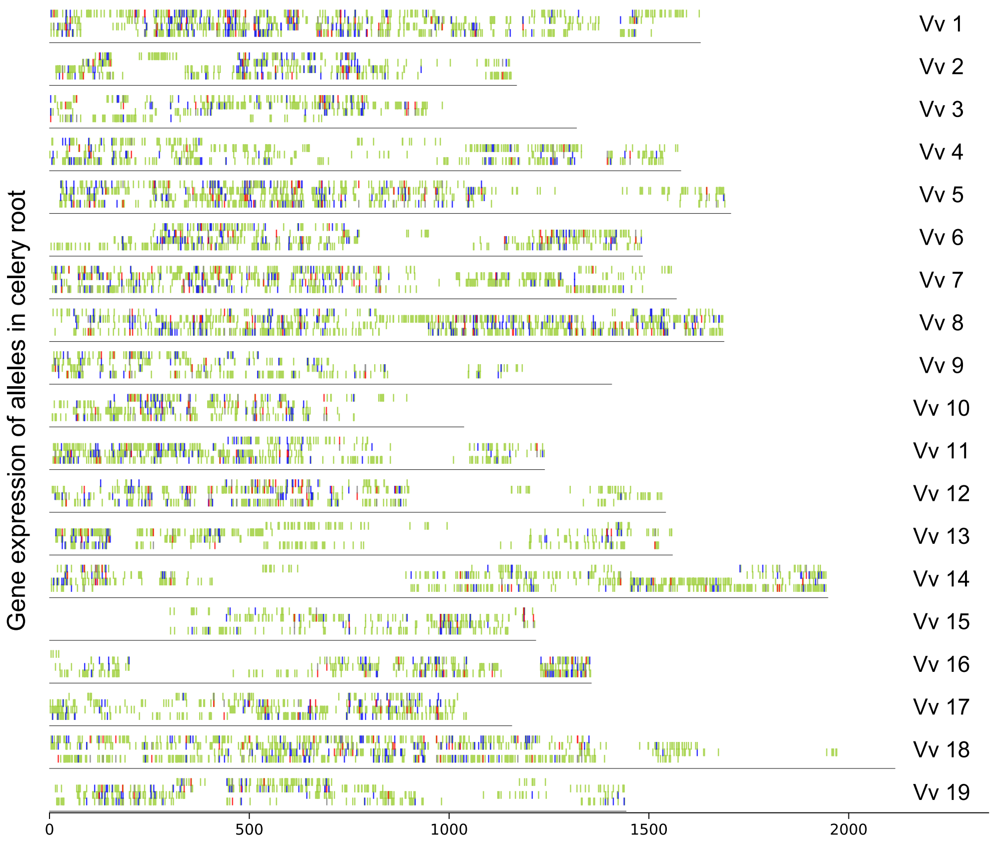

Supplementary Figure 26. (a) Expression of celery genes in root obtained using RNA-seq. Homoeologous regions produced by celery were grouped in subgenomes A1-A4 as to mapped grape chromosomes. Red (1.08%-1.71%) indicates a clear higher expression, blue (85.44%-87.62%) indicates no significant difference, and green (11.31%-13.35%) indicates a clear lower expression. No significant difference between A1-A4 subgenomes.

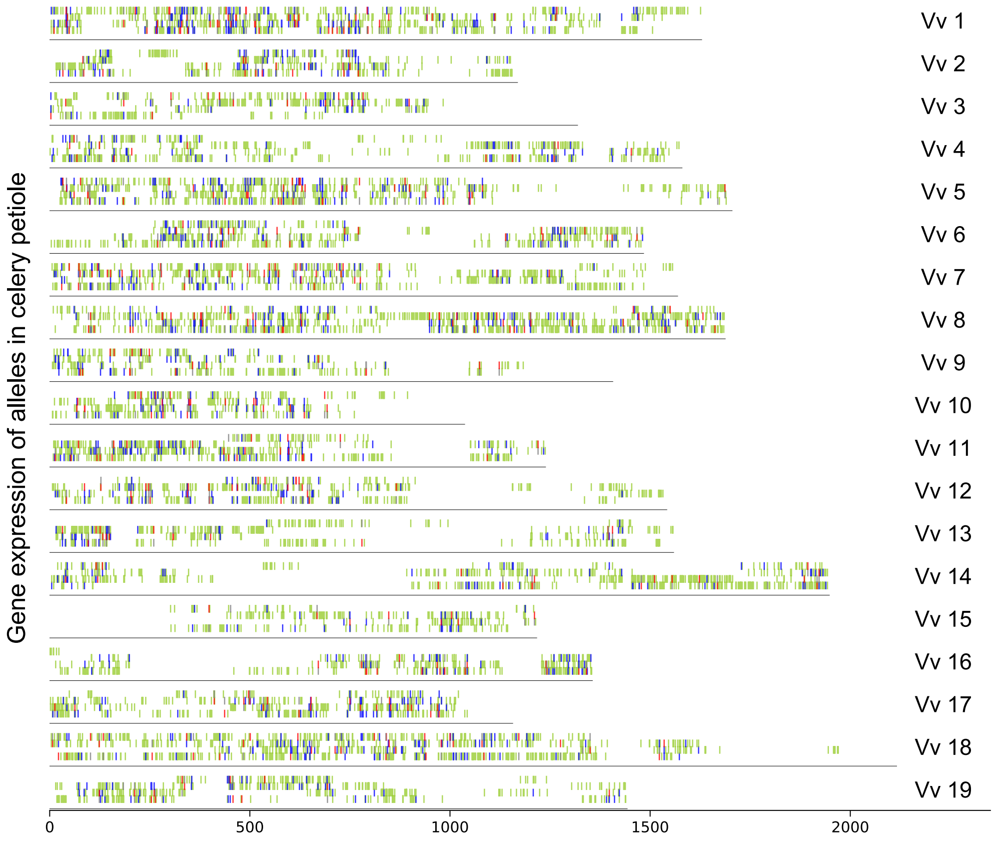

Supplementary Figure 26. (b) Expression of celery genes in petiole obtained using RNA-seq. Homoeologous regions produced by celery were grouped in subgenomes A1-A4 as to mapped grape chromosomes. Red (1.1%-1.63%) indicates a clear higher expression, green (85.86%-87.24%) indicates no significant difference, and blue (11.14%-13.0%) indicates a clear lower expression. No significant difference between A1-A4 subgenomes.

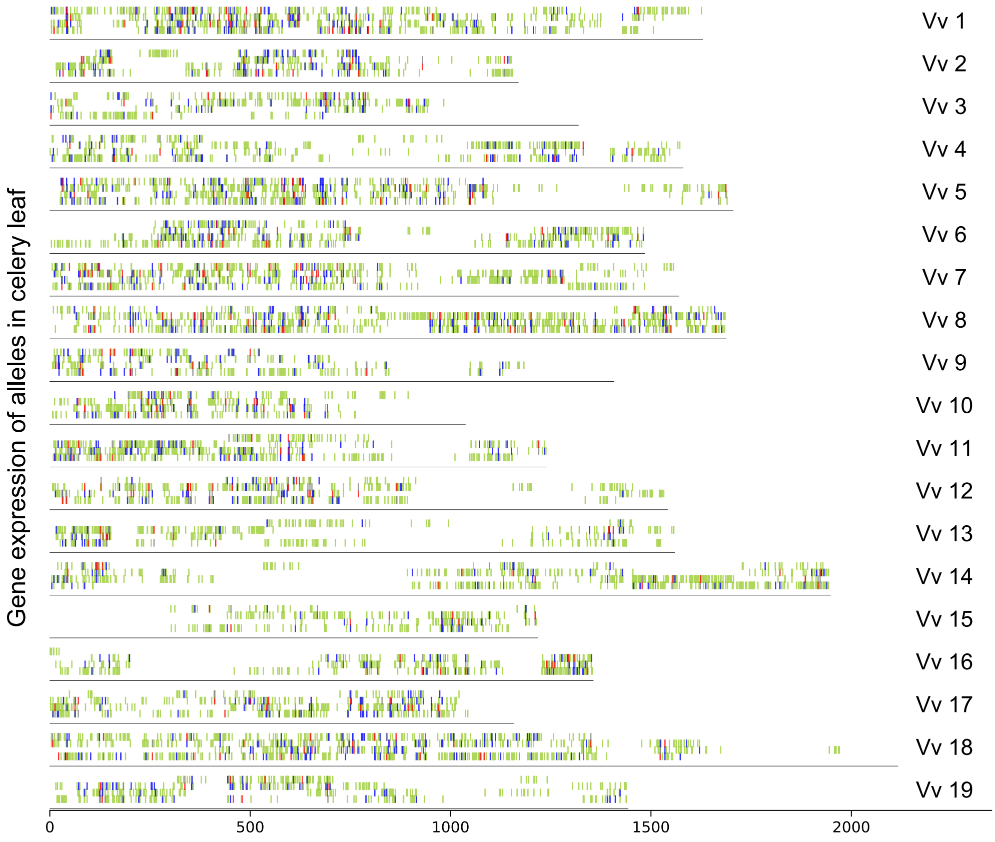

Supplementary Figure 26. (c) Expression of celery genes in leaf obtained using RNA-seq. Homoeologous regions produced by celery were grouped in subgenomes A1-A4 as to mapped grape chromosomes. Red (0.86%-1.44%) indicates a clear higher expression, green (86.0%-87.63%) indicates no significant difference, and blue (10.93%-12.81%) indicates a clear lower expression. No significant difference between A1-A4 subgenomes.

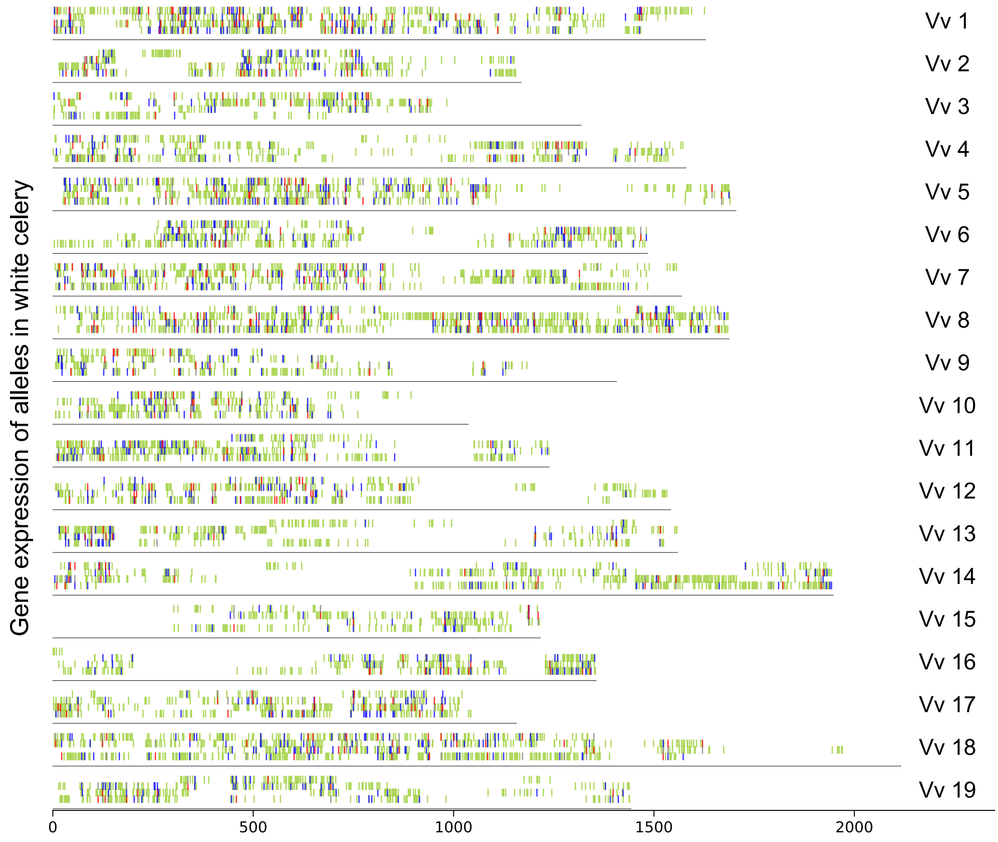

Supplementary Figure 26. (d) Expression of celery genes in white-stem variety obtained using RNA-seq. Homoeologous regions produced by celery were grouped in subgenomes A1-A4 as to mapped grape chromosomes. Red (1.21%-1.77%) indicates a clear higher expression, green (85.62%-87.21%) indicates no significant difference, and blue (10.99%-13.17%) indicates a clear lower expression. No significant difference between A1-A4 subgenomes.

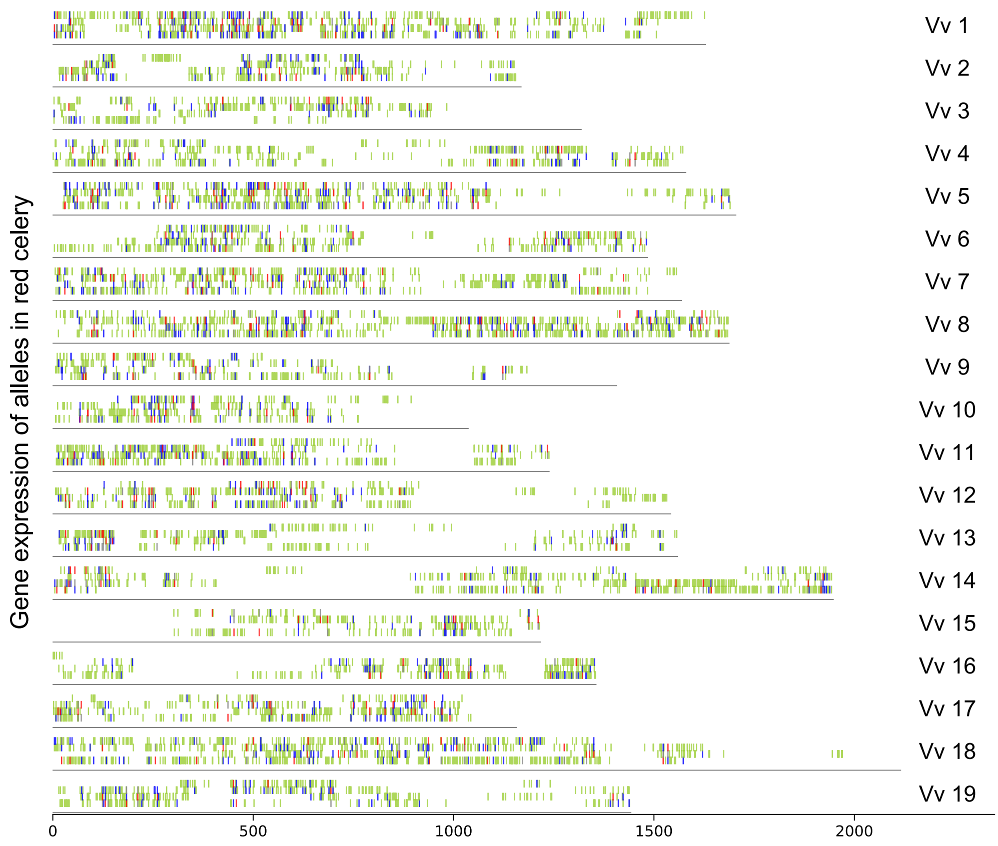

Supplementary Figure 26. (e) Expression of celery genes in red-stem variety obtained using RNA-seq. Homoeologous regions produced by celery were grouped in subgenomes A1-A4 as to mapped grape chromosomes. Red (0.97%-1.70%) indicates a clear higher expression, green (86.46%-87.62%) indicates no significant difference, and blue (10.69%-12.57%) indicates a clear lower expression. No significant difference between A1-A4 subgenomes.

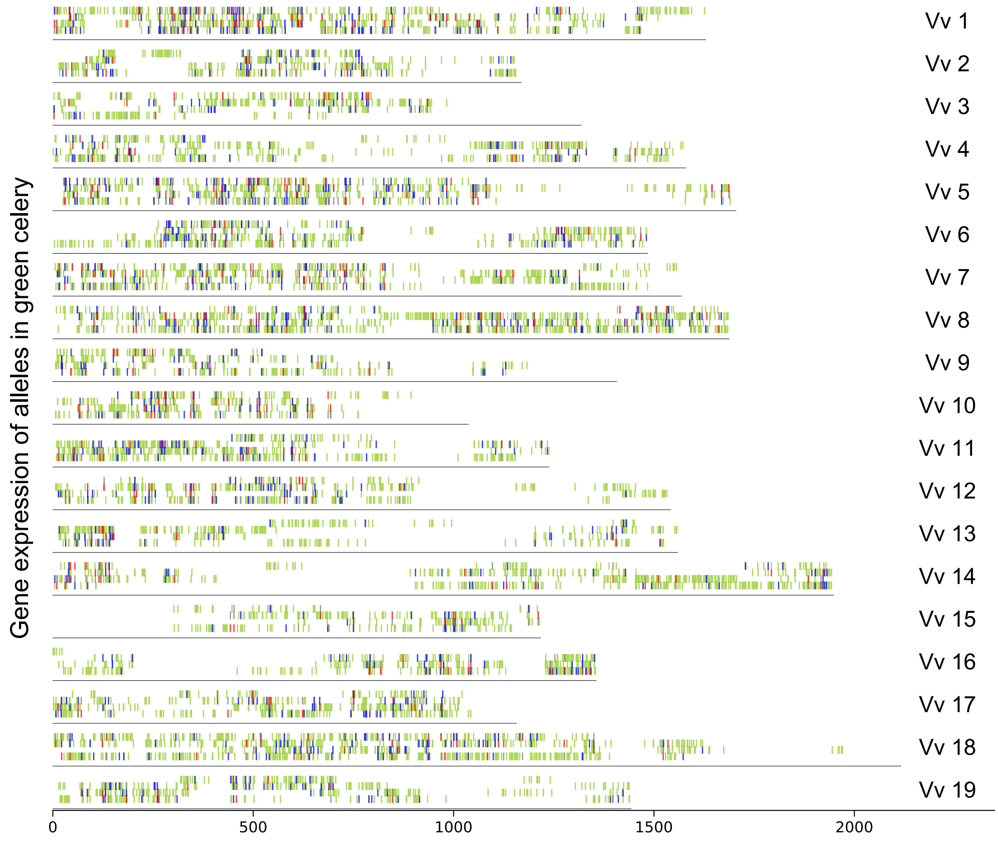

Supplementary Figure 26. (f) Expression of celery genes in green-stem variety obtained using RNA-seq. Homoeologous regions produced by celery were grouped in subgenomes A1-A4 as to mapped grape chromosomes. Red (0.93%-1.66%) indicates a clear higher expression, green (86.81%-88.06%) indicates no significant difference, and blue (10.28%-12.03%) indicates a clear lower expression. No significant difference between A1-A4 subgenomes.

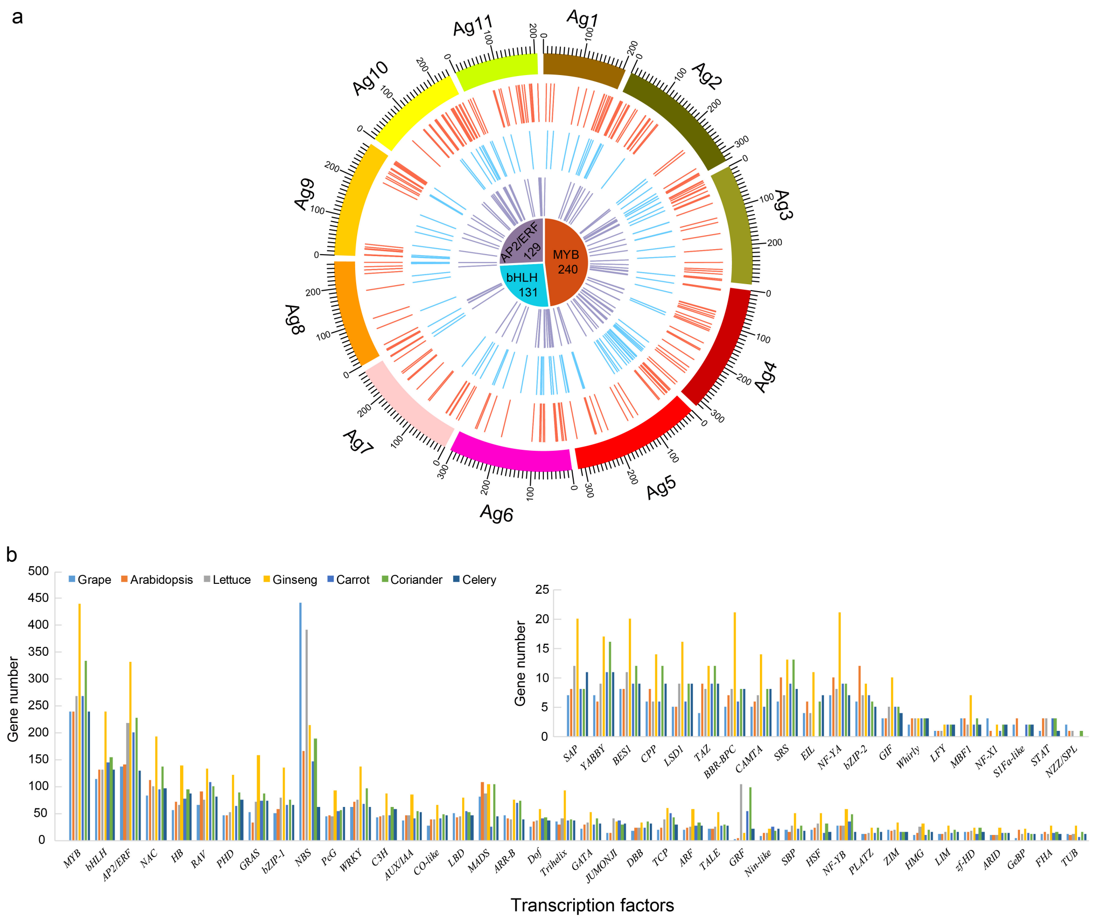

Supplementary Figure 27. Comparative analysis of the transcription factor families of celery and 6 representative plants. (a) The distribution of 3 largest transcription factor families on 11 chromosomes in celery. Red, blue, and purple colours indicate the *MYB*, *bHLH*, and *AP2/ERF* gene families, respectively. (b) The number of each transcription factor in celery, grape, Arabidopsis, lettuce, ginseng, carrot, and coriander genomes.

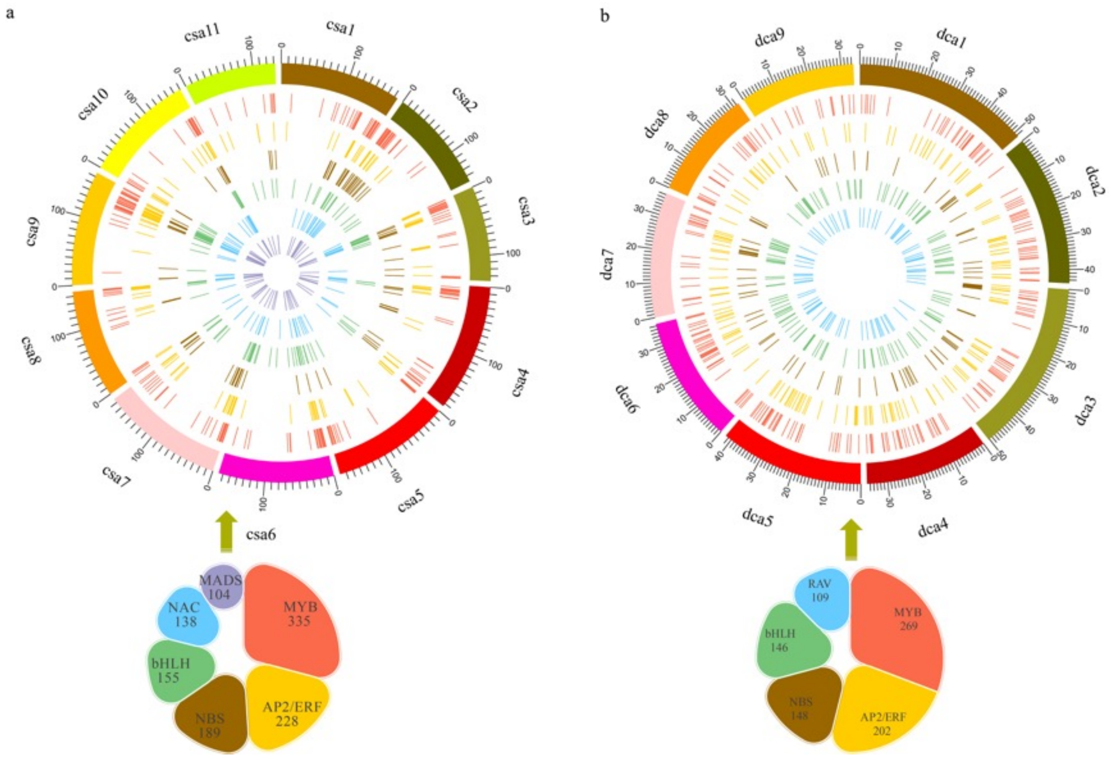

Supplementary Figure 28. Distribution of the transcription factor families with the number larger than 100. (a) The distribution of 6 largest transcription factor families on 11 chromosomes in coriander. (b) The distribution of 5 largest transcription factor families on 9 chromosomes in carrot.

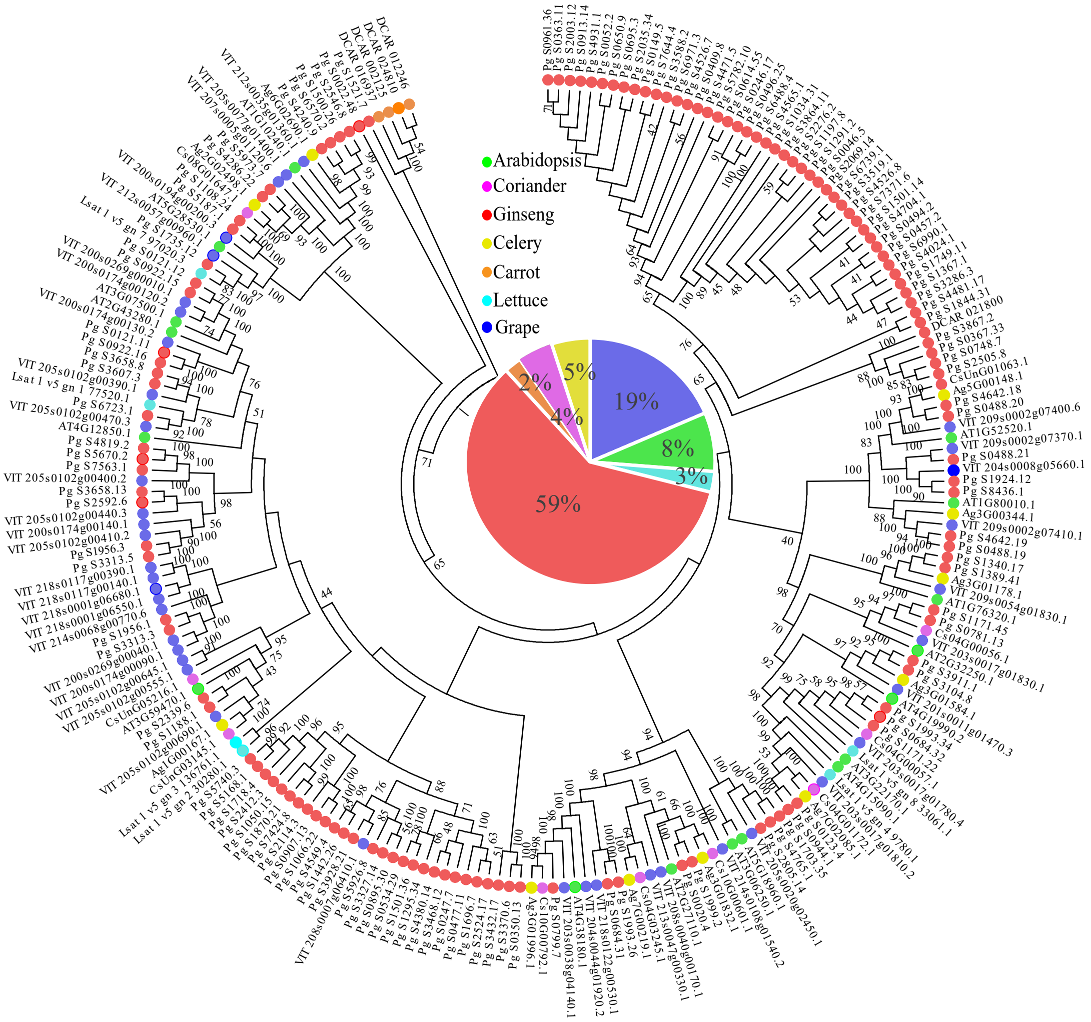

Supplementary Figure 29. (a) A phylogenetic tree of *FAR1* gene family constructed by the protein sequences of celery, coriander, carrot, lettuce, and grape. The phylogenetic tree topology was generated via MEGA 7.0. For the major nodes, neighbour-joining (NJ) bootstrap values above 40% are shown.

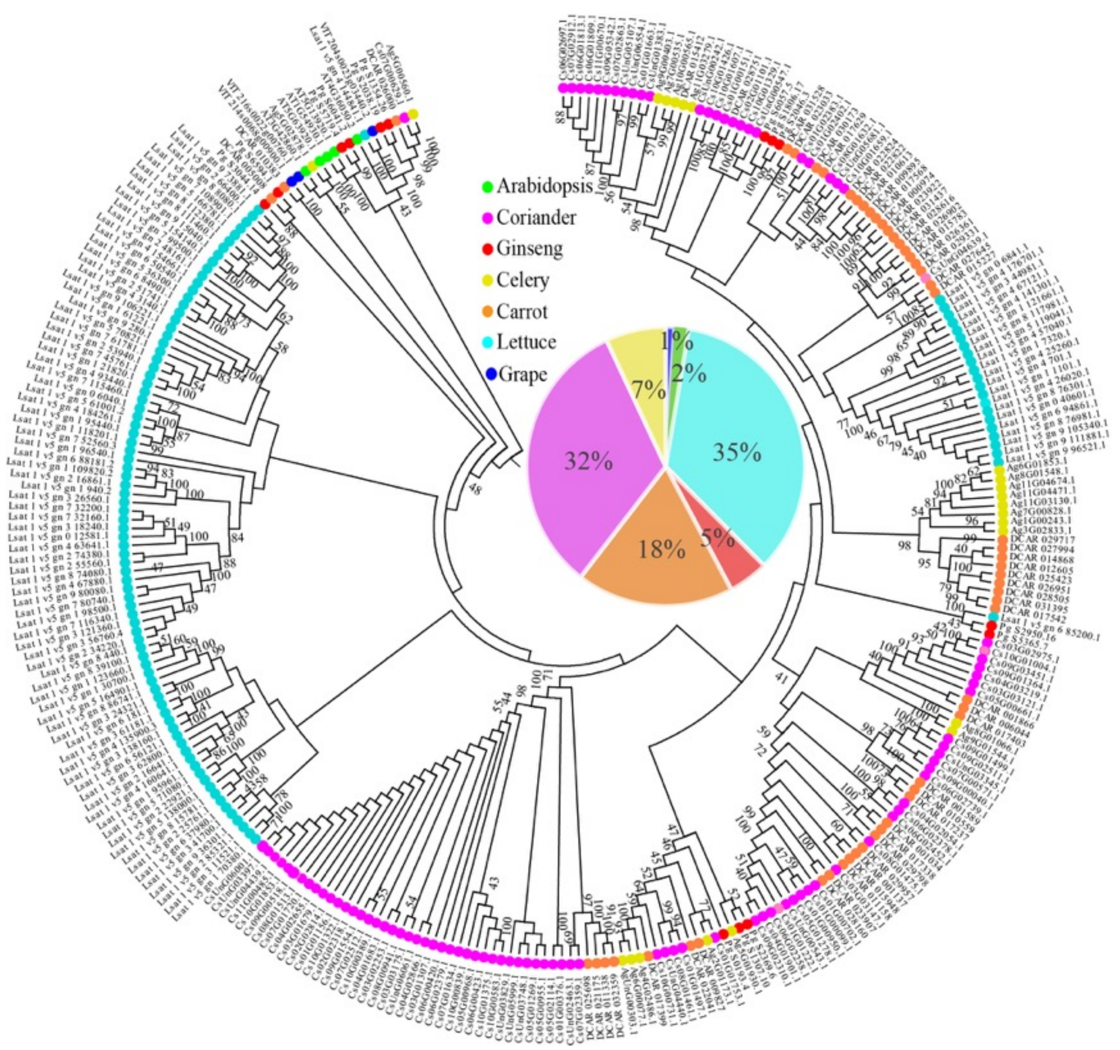

Supplementary Figure 29. (b) A phylogenetic tree of *GRF* gene family constructed by the protein sequences of celery, coriander, carrot, lettuce, and grape. The phylogenetic tree topology was generated via MEGA 7.0. For the major nodes, neighbour-joining (NJ) bootstrap values above 40% are shown.

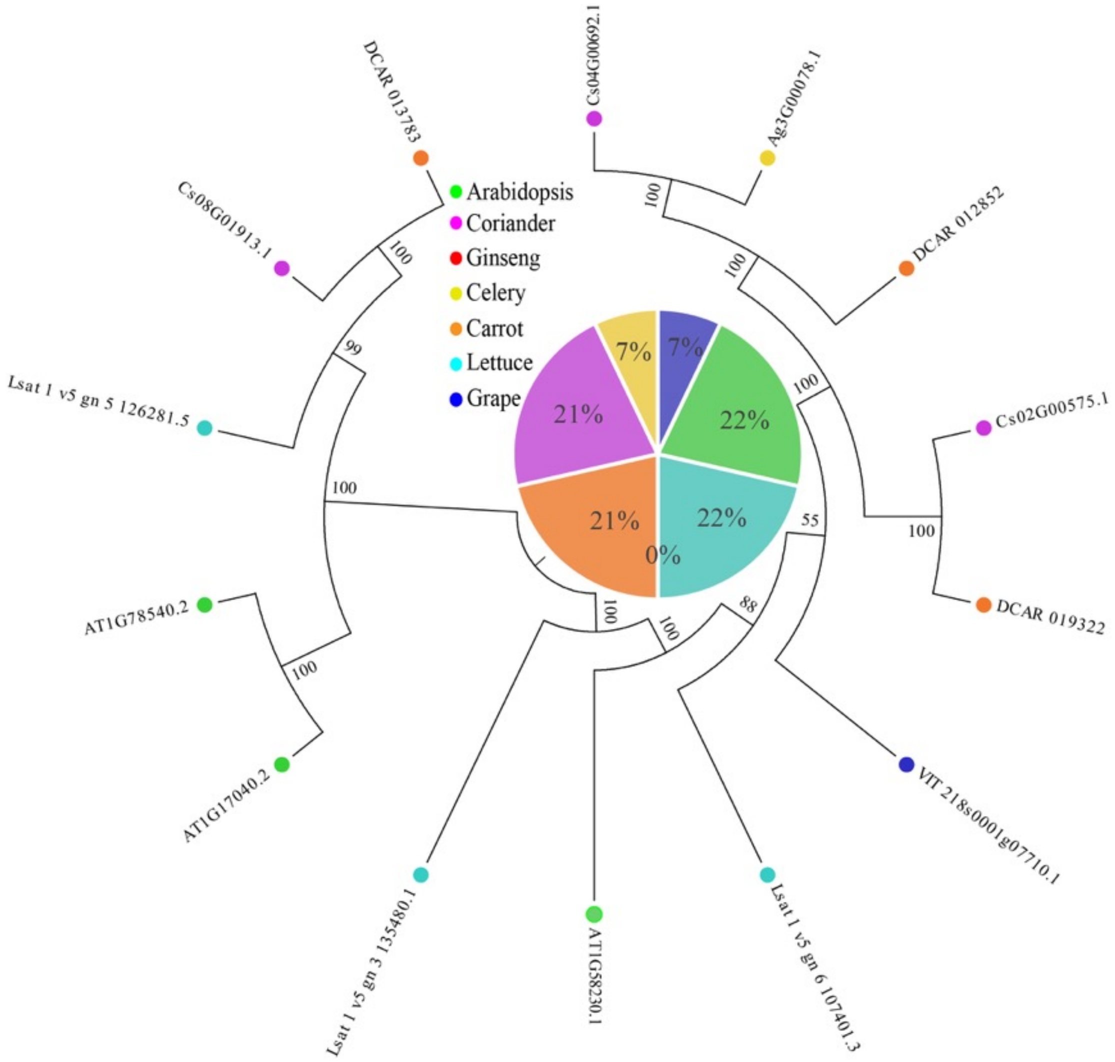

Supplementary Figure 29. (c) A phylogenetic tree of *STAT* gene family constructed by the protein sequences of celery, coriander, carrot, lettuce, and grape. The phylogenetic tree topology was generated via MEGA 7.0. For the major nodes, neighbour-joining (NJ) bootstrap values above 40% are shown.

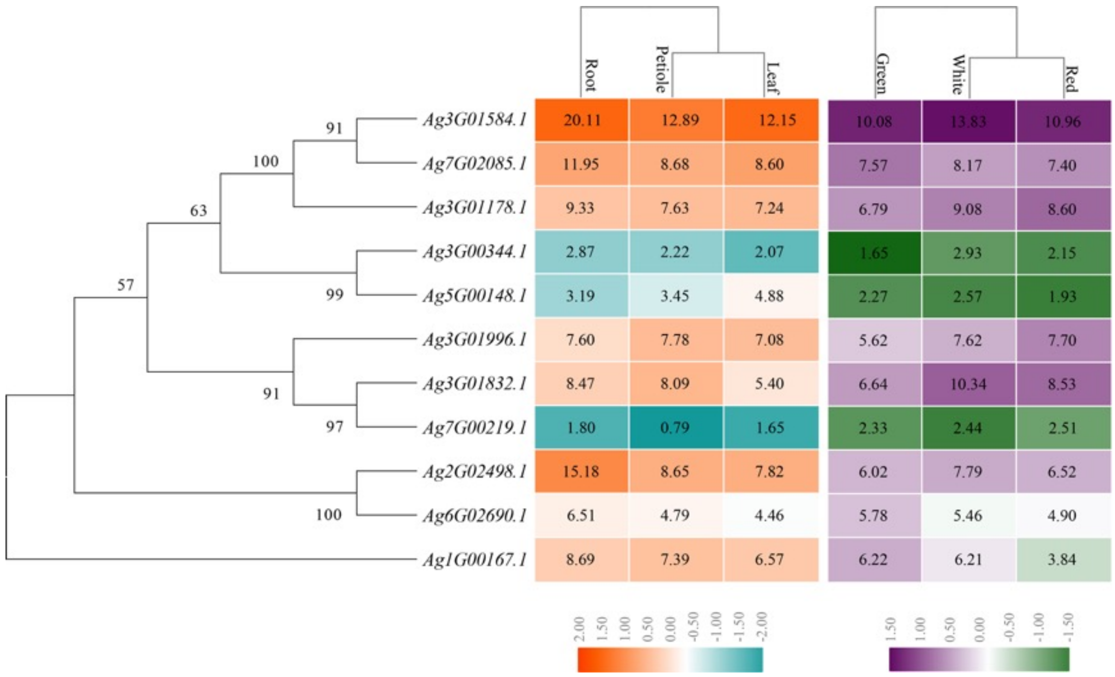

Supplementary Figure 30. (a) A FPKM hierarchical clustering map of the *FAR1* gene family. The left heatmap represents the expression values of three tissues (root, petiole, leaf) of celery. The right heatmap represents the expression values of three different colors' varieties (green, red, and white) of celery.

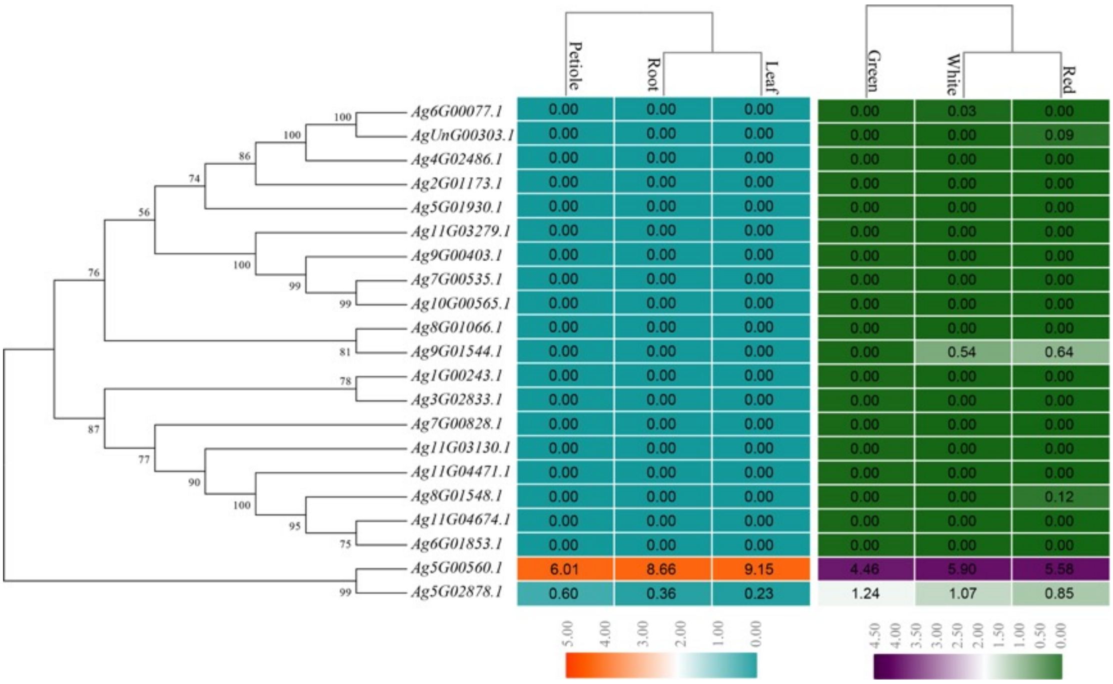

Supplementary Figure 30. (b) A FPKM hierarchical clustering map of the *GRF* gene family. The left heatmap represents the expression values of three tissues (root, petiole, leaf) of celery. The right heatmap represents the expression values of three different colors' varieties (green, red, and white) of celery.

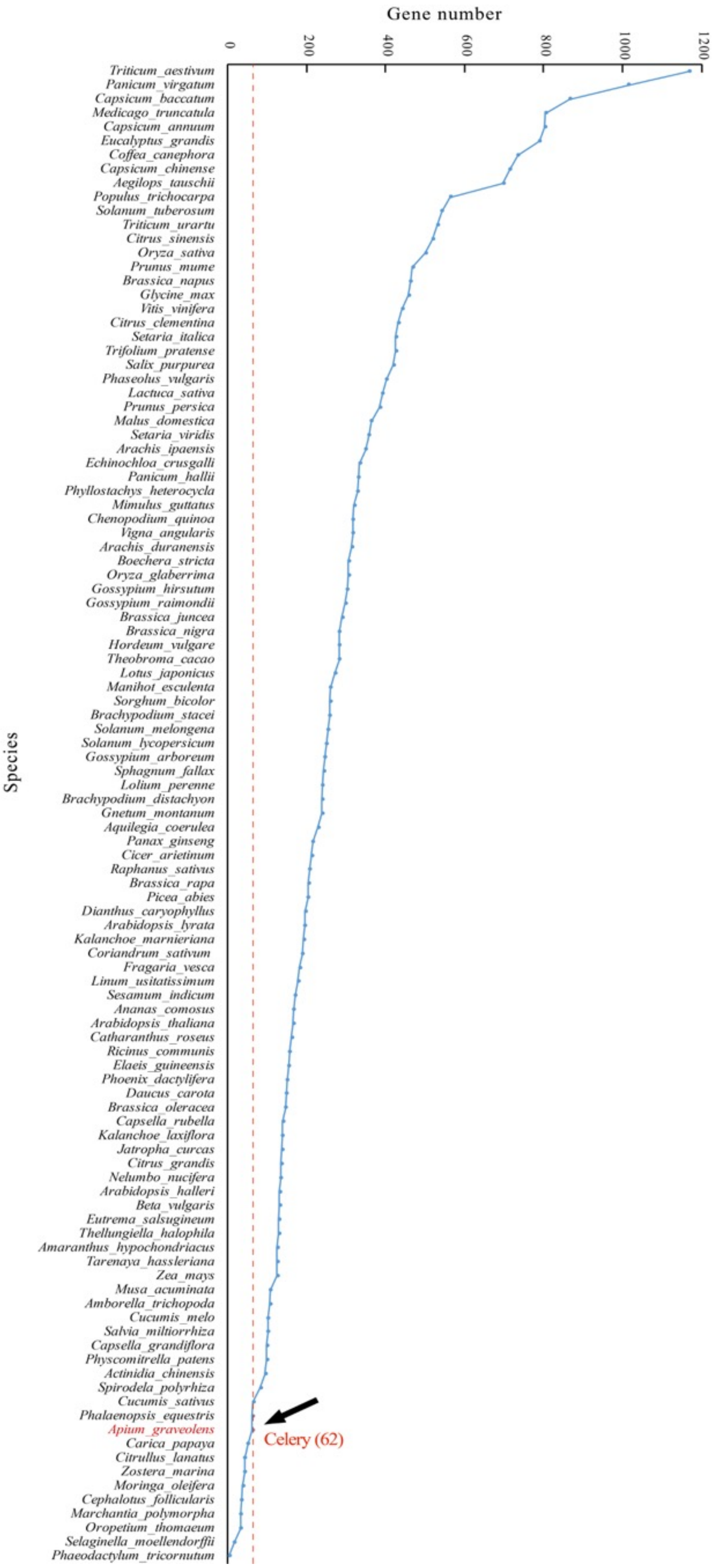

Supplementary Figure 31. Numbers of *NBS* family genes in celery and 106 plants.

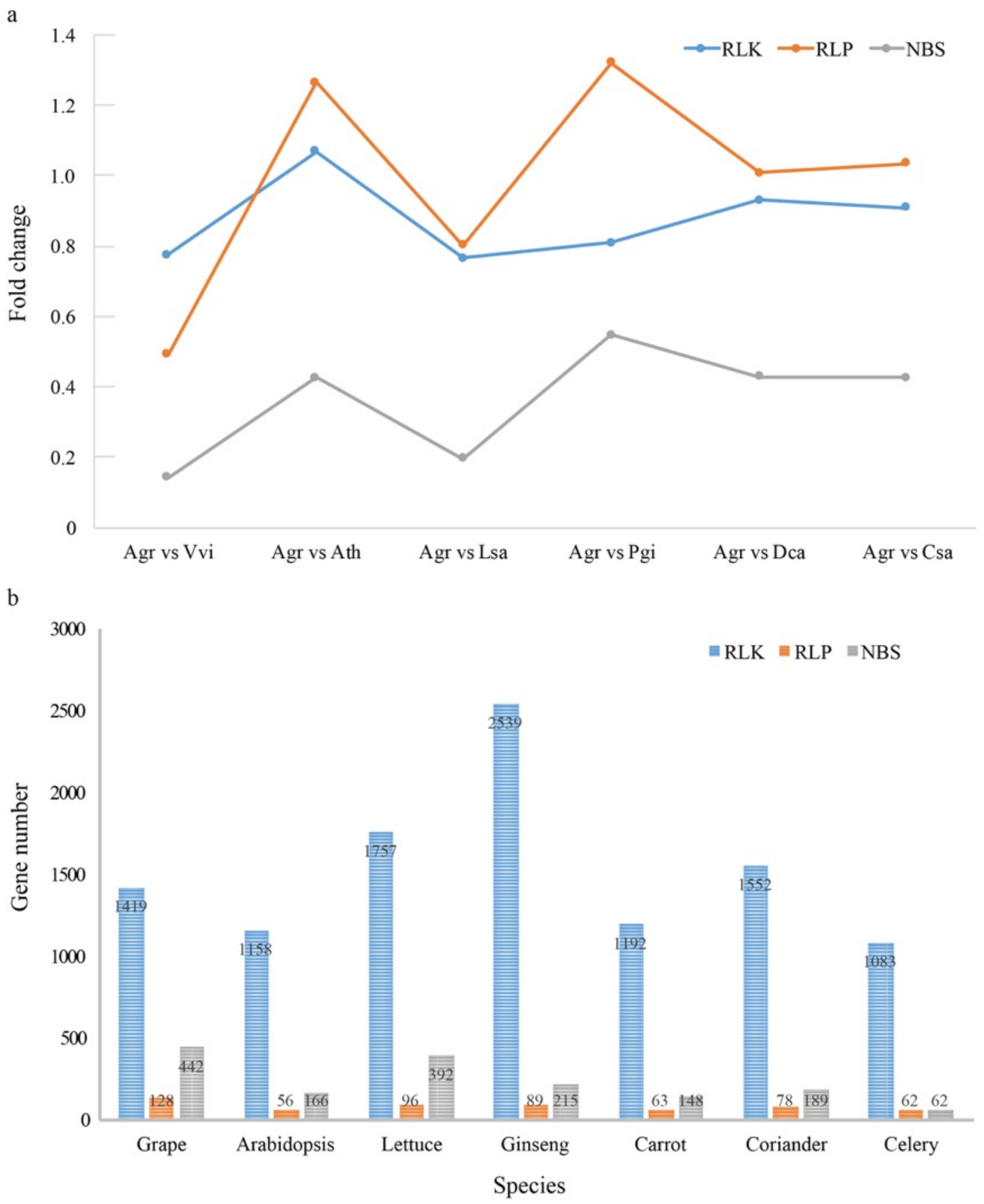

Supplementary Figure 32. Analyses of *R*-genes, including *RLK*, *RLP*, and *NBS* genes. (a) the fold change of *RLK*, *RLP*, and *NBS* genes number between celery (Agr) and grape (Vvi), Arabidopsis (Ath), lettuce (Lsa), ginseng (Pgi), carrot (Dca), and coriander (Csa). (b) The number of *RLK*, *RLP*, and *NBS* family genes in grape, Arabidopsis, lettuce, ginseng, carrot, and coriander, and celery.

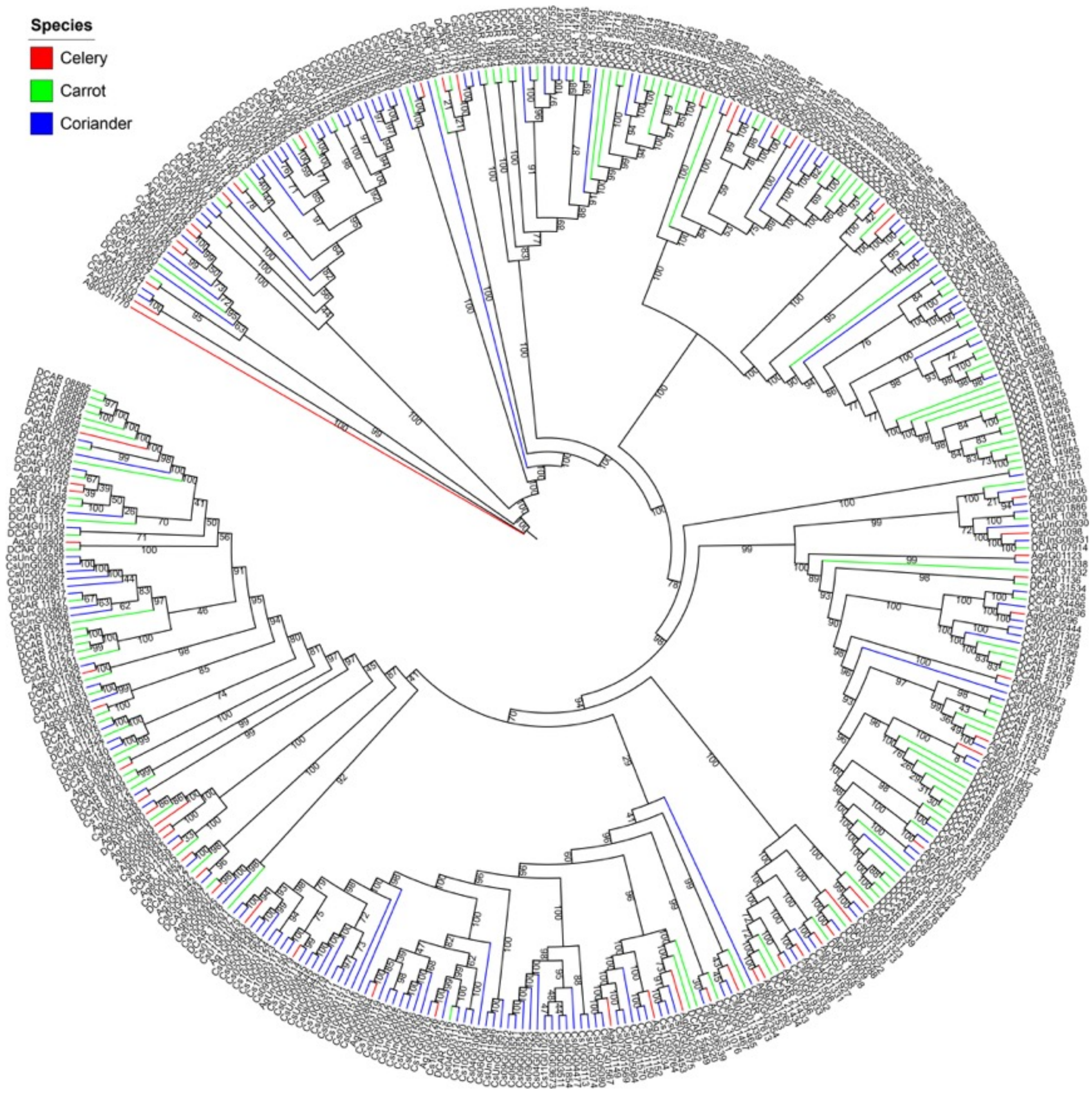

Supplementary Figure 33. A phylogenetic tree of NBS gene family constructed by the protein sequences of celery, coriander, and carrot. The phylogenetic tree topology was generated via MEGA 7.0. For the major nodes, neighbour-joining (NJ) bootstrap values above 40% are shown.

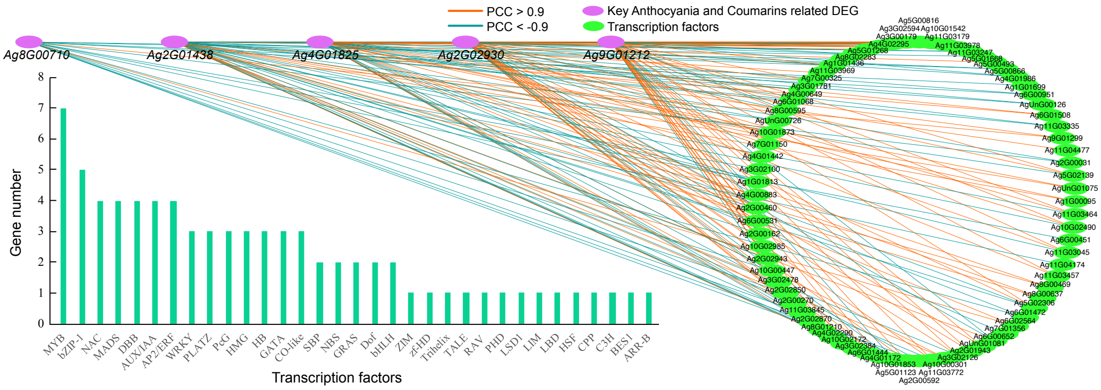

Supplementary Figure 34. The interaction network between key anthocyanins and coumarins related DEGs (purple oval) and differently expressed TFs (DETs, green oval) between red variety and both green or white varieties. The orange and blue lines represent the Pearson correlation coefficient (PCC) values larger than 0.9 (positively regulated relationship) or lower than -0.9 (negatively regulated relationship), respectively. The histogram shows the number of each transcription factor category in the regulatory network.
