# Supplementary material for: The celery genome sequence reveals sequential paleo‐polyploidizations, karyotype evolution and resistance gene reduction in apiales
Source: Plant Biotechnol J. 2020 Nov 18;19(4):731–44. doi: 10.1111/pbi.13499 (PMC8051603; doi:10.1111/pbi.13499)
Supplement: Supplementary file 3 — Note S1. Supplementary Notes 1‐6. [file PBI-19-731-s001.pdf]

The Celery Genome Sequence Reveals Sequential Paleo-Polyploidizations, Karyotype Evolution, and Resistance Gene Reduction in Apiales

Xiaoming Song[1,2,3*], Pengchuan Sun[1*], Jiaqing Yuan[1*], Ke Gong[1], Nan Li[1], Fanbo Meng[1], Zhikang Zhang[1], Xinyu Li[1], Jingjing Hu[1], Jinpeng Wang[1,2], Qihang Yang[1], Beibei Jiao[1], Fulei Nie[1], Tao Liu[1], Wei Chen[1], Shuyan Feng[1], Qiaoying Pei[1], Tong Yu[1], Xi Kang[1], Wei Zhao[1], Chunlin Cui[1], Ying Yu[1], Tong Wu[1], Lanxing Shan[1], Man Liu[1], Zhiji Qin[1], Hao Lin[3], Rajeev K. Varshney[4], Xiu-Qing Li[5], Andrew H. Paterson[1,6], Xiyin Wang[1,2#]

[1] School of Life Sciences/Center for Genomics and Bio-computing, North China University of Science and Technology, Tangshan, Hebei 063210, China;

[2] National Key Laboratory for North China Crop Improvement and Regulation, Hebei Agriculture University, Baoding, Hebei 071001, China;

[3] School of Life Science and Technology and Center for Informational Biology, University of Electronic Science and Technology of China, Chengdu 610054, China;

[4] Center of Excellence in Genomics & Systems Biology, International Crops Research Institute for the Semi-Arid Tropics (ICRISAT), Patancheru-502 324, India;

[5] Fredericton Research and Development Centre, Agriculture and Agri-Food Canada, Fredericton, New Brunswick, E3B 4Z7, Canada;

[6] Plant Genome Mapping Laboratory, University of Georgia, Athens, GA, 30605, USA.

[*]These authors contributed equally to the work.

[#]Correspondence should be addressed to Xiyin Wang: wangxiyin@vip.sina.com

Directory

1. Survey of celery genome 5

1.1 Introduction 5

1.2 Experimental methods 5

1.3 Output of sequencing data and quality control 5

1.3.1 Data output 5

1.3.2 Data filtering methods 5

1.3.3 Quality control 5

1.4 K-mer analysis 6

2. Preliminary celery genome assembly 6

2.1 Data error correction 6

2.2 10X genomics assisted third generation data assembly 6

2.3 Assembly results 8

2.3.1 Sequencing data statistics 8

2.3.2 Assembly result statistics 8

2.3.3 Genomic base composition 8

2.4 Assembly results evaluation 8

2.4.1 Sequence consistency assessment 8

2.4.2 Sequence integrity assessment 9

3. Hi-C technology assisted genome assembly 9

3.1 Introduction 9

3.2 Experimental procedure 9

3.2.1 Hi-C biotin labeling 10

3.2.2 Library construction 10

3.2.3 Library Check 10

3.2.4 Sequencing 10

3.3 Bioinformatics analysis 10

3.4 Sequencing data quality control 11

3.4.1 Original sequencing data 11

3.4.2 Sequencing data statistics 11

3.4.3 Sequencing data quality assessment 11

3.5 Hi-C technology assisted genome assembly 11

3.5.1 Comparison with draft genome 11
3.5.2 Clustering 12
3.5.3 Sorting and Orientation 12
3.5.4 Assembly result statistics 12

4. Genome prediction and annotation 12

4.1 Analysis process and method 12
4.1.1 Genome prediction 12
4.1.2 Genome annotation 13

4.2 Analysis results 14
4.2.1 Repeat sequence annotation 14
4.2.2 Tandem repeat analyses 14
4.2.3 Centromeres and telomeres prediction 15
4.2.4 Gene structure annotation 16
4.2.5 Gene annotation 17
4.2.6 rRNA, tRNA, snRNA, miRNA annotation 17

5. RNA-seq 17

5.1 Introduction 17

5.2 Library construction and sequencing 17
5.2.1 RNA detection 17
5.2.2 Library construction 17
5.2.3 Library inspection 18
5.2.4 Sequencing 18

5.3 Bioinformatics analysis 18
5.3.1 Original sequences data 18
5.3.2 Data quality assessment 18

6. Comparative genomic analyses 18

6.1 Materials and Methods 18
6.1.1 Gene family analysis 19
6.1.2 Phylogenetic tree construction and divergence time estimation 19
6.1.3 Inference of gene colinearity, Ks calculation, distribution fitting, and correction 19
6.1.4 Reconstruction of ancestral karyotypes of Apiales plants 23

6.2 Results 23
6.2.1 Gene colinearity within and among genomes 23

6.2.2 Two paleo-polyploidization events ... 24
6.2.3 Multiple alignment ... 25
6.2.4 Genomic fractionation ... 25
6.2.5 RNA-seq analyses ... 26
6.2.5.2 Alignment analysis ... 26
6.2.5.3 Gene expression analysis ... 26
6.2.5.4 RNA-seq quality assessment ... 27
6.2.5.5 Differentially expressed genes (DEGs) ... 27
6.2.5.6 Differential expressed genes analysis ... 27
6.2.6 Gene expression balance analyses ... 27
6.2.7 Celery chromosomes representing the Apiaceae proto-chromosomes ... 28
6.2.8 Comparative analyses of transcription factor families ... 33
7. References ... 37

1. Survey of celery genome

1.1 Introduction

The survey was conducted for the celery (*Apium graveolens*) genome size, heterozygosity rate, and repeat sequence ratio estimation. Here, we estimated the celery genome size using Kmer method, which is a popular way used in almost every genome sequencing project (Marcais and Kingsford, 2011). In this study, we constructed three small fragment of the libraries, and then carried out Illumina HiSeq PE sequencing.

1.2 Experimental methods

Firstly, general standards and methods were used for DNA extraction by Phenol-Chloroform (Sambrook and Russell, 2006). Qualified DNA sample was randomly interrupted into a length of 350 bp fragment using Covaris ultrasonic crusher. Secondly, the fragment was repaired by the end, added A Tail, plus sequencing joints, purification, PCR amplification to complete the entire library preparation. Finally, the constructed libraries were sequenced using Illumina HiSeq 4000.

1.3 Output of sequencing data and quality control

1.3.1 Data output

The production of sequencing data is through the DNA extracting, building, and sequencing steps. The original image data obtained by sequencing base calling into sequence data, which we called raw data with the FASTQ format. The original sequencing data contained the adapter, low-quality bases, and an undefined base (N). These can cause significant disruption to subsequent bioinformatics analyses. So we used the filtering methods to remove the interference information to obtain the clean data.

1.3.2 Data filtering methods

The Filter methods were mainly from the following three aspects:

1) We removed the reads containing the adapter sequences;

2) The content of N contained in single-ended read exceeds that 10% length of read need to remove.

3) The single-end sequencing read contains low quality (<5) base exceeds 20% of the read length need to remove.

1.3.3 Quality control

1.3.3.1 Data statistics

We obtained the high quality clean data after a series of strict filtering. Then we summarized the sequencing output data features, including read quantity, data yield, error rate, Q20, Q30, and GC content (Supplementary Table 1).

1.3.3.2 Data evaluation and conclusion

Original sequencing data of celery is 181.27 Gb in total. The sequencing data was of high quality (Q20 ≥90%, Q30 ≥85%), and sequencing error rate was rather low (<0.05%). Nucleotide library comparison revealed there was no contamination in the sample.

1.4 K-mer analysis

We adopted K-mer to estimate the celery genome size and hybridization rate, that is, from a continuous sequence to iteratively select the length of K base sequence. If the length of each sequence is L, the k-mer length is K, we can get the L-K+1 k-mer. Here we took k =17 to perform the analysis.

According to the survey analysis, the main peak is near depth =22 (Supplementary Fig. 1). The genome size estimated (Kmer-number/depth) is about 3,475.41 Mb, and the corrected genome size is 3,453.78 Mb. The genomic heterozygosity rate was 0.20%, and the repeat sequence ratio was 87.10% (Supplementary Table 2).

2. Preliminary celery genome assembly

2.1 Data error correction

The process of error correction firstly established a K-mer frequency table with sequencing data. After setting cutoff, the K-mers can be divided into high frequency and low frequency ones. For reads with low-frequency K-mers, we made the K-mers of the entire reads high by changing some bases. Then we corrected potential errors possibly caused by sequencing. The large segments do not need to be used in this error correction process, therefore data correction is usually performed on small segment library data. The genome error correction was conducted using second and third sequencing data by Pilon (https://github.com/broadinstitute/pilon/wiki) and Quiver software with the default parameters, respectively (Chin et al., 2013; Walker et al., 2014).

2.2 10X genomics assisted third generation data assembly

(1) Extraction of genomic DNA (>50Kb)

(2) Third-generation database construction. The library of single molecule real-time (SMRT) PacBio genome sequencing was constructed according to the standard protocols of Pacific Bioscience company. Briefly, high molecule genomic DNA was sheared to ~20 Kb targeted size, followed by damage repair and end repair, blunt-end adaptor ligation, and size selection. Finally, the library was sequenced using the PacBio Sequel platforms.

Details can be described as follows: 1) DNA adaptor with hairpin structure were attached to both ends of double-stranded DNA. 2) The Pacbio sequencing data was self-corrected. 3) Genome assembling using the third generation data were conducted after error correction. The assembly was performed by using the Overlap-Layout-Consensus (OLC) algorithm. 4) All third generation data were sequenced for mapping. The assembly was further corrected to improve the accuracy, and finally obtained the contig sequences.

The Falcon software (https://github.com/PacificBiosciences/FALCON) was used for the genome assemble with the parameters, falcon_sense_option = --output_multi --min_idt 0.70 --min_cov 3 --max_n_read 300 --n_core 20 overlap_filtering_setting = --max_diff 500 --max_cov 500 --min_cov 2 --bestn 10 --n_core 36(Chin et al., 2016).

(3) 10X Genomics library construction. For the 10X library construction, read 1 sequence and the 10X™ barcode were added to the molecules during the GEM incubation. P5 and P7 primers, read 2, and Sample Index were added during library construction via end repair, A-tailing, adaptor ligation, and amplification. The final libraries contain the P5 and P7 primers used in Illumina® bridge amplification. Details as follows. The gel beads were connected with: 1) illuminaP5 connector. 2) 16 base Barcode. 3) Illumina read 1 sequencing primers. 4) 10-bp random sequence primers. The Barcode primer were combined DNA and enzyme mixtures through two intersections, then placed on a special 96-plate for 10X Genomics library preparation. After PCR amplification, further processing includes breaking the oil droplets, mixing different Barcode sequences, breaking into fragments, and adding P7 linker for sequencing were done.

(4) Comparison of the linked-reads to the contigs of third-generation sequencing.

(5) For contig/scaffold, there were many linked-reads that supported their connection when the actual distance was relatively close. However, the linked-reads support was missing and could not be connected when being far away from actual distance.

The 10X technology was used for assisting genome assembly using fragScaff software (https://sourceforge.net/projects/fragscaff/files/) with the parameters, -fs1 '-m 3000 -q 30 -E 30000 -o 60000' -fs2 '-C 5' -fs3 '-j 2 -u 3'(Adey et al., 2014).

2.3 Assembly results

2.3.1 Sequencing data statistics

The celery genome was sequenced using the third-generation sequencing technology Pacbio sequel platform with a total of 269.85 Gb, and a coverage depth of 78.13X (Table 1; Supplementary Table 3). In addition, 10X Genomics library and second generation small fragments were constructed and sequenced using the Illumina HiSeq 4000 platform (Table 1).

2.3.2 Assembly result statistics

Assembly results were summarized from scaffolds above 100 bp. The contig N50 of the celery genome reached 845.61 Kb, and the scaffold N50 reached 2.53 Mb (Supplementary Table 4).

2.3.3 Genomic base composition

The ratio of GC is 35.68%, and the ratio of N is 0.81%, which was an acceptable range (<10%) (Supplementary Table 5).

2.4 Assembly results evaluation

2.4.1 Sequence consistency assessment

To evaluate the accuracy of the genome assembly, the small fragment library reads were mapped to the assembled celery genome using BWA software (http://bio-bwa.sourceforge.net/) (Jo and Koh, 2015). The mapping rate of all small fragments reads was about 99.71%, and the coverage rate was about 98.75%, indicating that the genomes of reads and assembly were well (Supplementary Table 6).

We used Samtools (http://samtools.s ourceforge.net/) to sort the BWA alignment results by chromosome coordinates. Then, we removed duplicate reads, performed single nucleotide polymorphisms (SNP) calling, and filtered the original results to obtain SNP (Etherington et al., 2015; Li et al., 2009). The ratio of SNP in the celery genome was 0.022%, and the ratio of homozygous SNP is 0.0002% (Supplementary Table 7). The homozygous SNP ratio can reflect the correct rate of genome assembly, indicating that the assembly had a high base correct rate.

The assembled genomic sequence was plotted with 10 Kb for windows. The sample was not contaminated according to the distribution of GC content and average depth. The GC content was concentrated around 35%, and there was no obvious separation of the scatter plots, indicating that there was no external pollution in the genome.

2.4.2 Sequence integrity assessment

2.4.2.1 CEGMA assessment

The integrity of celery genome assembly was evaluated by Core Eukaryotic Genes Mapping Approach (CEGMA) (Parra et al., 2007). The evaluation selected 248 core eukaryotic genes present in the six eukaryotic model organisms to form a core gene library. Then, we combined software, such as tBlastn, Genewise, and Geneid to evaluate the genome integrity (Birney et al., 2004). Eventually, we assembled 237 Core Eukaryotic Genes with a ratio of 95.56% (Supplementary Table 8).

2.4.2.2 BUSCO assessment

We used the Benchmarking Universal Single-Copy Orthologs (BUSCO, http://busco.ezlab.org/) to evaluate the genome integrity (Seppey et al., 2019; Waterhouse et al., 2019). The evaluation using a single-copy orthologous gene pool in conjunction with tBlastn, Augustus, and Hmmer programs. According to the BUSCO assessment results, the orthologous single-copy genes assembled 91.7% of complete single-copy genes (Supplementary Table 9).

3. Hi-C technology assisted genome assembly

3.1 Introduction

The Hi-C technology was further used to assist celery genome assembly. The libraries were sequenced using Illumina HiSeq 4000. The analyses mainly contained the data quality control, mapping the genomes, clustering, sorting, orientation, accuracy assessment for the genome.

3.2 Experimental procedure

3.2.1 Hi-C biotin labeling

Chromatin was digested for 16 h with 400 U HindIII restriction enzyme (NEB) at 37 °C. DNA ends were labeled with biotin and incubated at 37 °C for 45 min, and the enzyme was inactivated with 20% SDS solution. The specific steps as follows.

(1) Using cell cross-linking agent paraformaldehyde to make DNA and cell combined;

(2) Using the restriction enzyme to deal with the cross-linked DNA;

(3) Adding biotin label at the end of oligonucleotide;

(4) Using nucleic acid ligase to make the adjacent DNA fragments linked;

(5) The protease digests the protein at the junction to de-crosslink protein and DNA. DNA was extracted and randomly broken into fragments of 350 bp by Covaris crusher.

3.2.2 Library construction

Capture DNA with biotin under the adsorption of avidin magnetic beads. The mainly steps contained the end-repair, addition of A, linker ligation, PCR amplification and purify to complete the entire library preparation. Specifically, DNA ligation was performed by the addition of T4 DNA ligase (NEB) and incubation at 16°C for 4~6 h. After ligation, proteinase K was added to reverse cross-linking during incubation at 65 °C overnight. DNA fragments were purified and dissolved in 86μL of water. Unligated ends were then removed. Purified DNA was fragmented to a size of 300–500 bp, and DNA ends were then repaired. DNA fragments labelled by biotin were finally separated on Dynabeads® M-280 Streptavidin (Life Technologies).

3.2.3 Library Check

Using Qubit 2.0, we performed preliminary quantification, and the library was diluted to 1 ng/μl. Then we tested the insert size of library followed by Agilent 2100. If the insert size was as expected, starting accurate quantification to the effective concentration of the library by Q-PCR (the library effective concentration >2 nM).

3.2.4 Sequencing

Different libraries were pooled according to the effective concentration and the target data volume, and then using Illumina HiSeq X Ten to sequence.

3.3 Bioinformatics analysis

The steps of Hi-C are mainly as follows:

(1) Quality control of raw data to obtain clean data;

(2) Mapping the clean data to the celery genome;

(3) Clustering, sorting, orienting, and assisting genome to anchor the chromosome.

3.4 Sequencing data quality control

3.4.1 Original sequencing data

Please refer to the section 1.3.1.

3.4.2 Sequencing data statistics

Please refer to the section 1.3.2.

3.4.3 Sequencing data quality assessment

The total of sequencing data for Hi-C is 378.06 Gb is with the high sequencing quality (Q20 ≥90%, Q30 ≥85%). The GC distribution is normal, and the sample is not contaminated (Supplementary Table 10). The Hi-C construction library has a relative high quality. The finally valid read pairs is 3,000,276, and the average data effect rate is 34.89% (Supplementary Table 11).

3.5 Hi-C technology assisted genome assembly

Hi-C analysis produced spatially connected DNA fragments, showing interactions between distantly located DNA fragments. According to whether the interaction probability inside the chromosome is higher than that of between two chromosomes, and the contig or scaffold were divided into different chromosomes. According to the interaction probability decreases with the increase of the interaction distance on the same chromosome, sorting and orienting the contig or scaffold of the same chromosome was performed (Fig. 1).

Hi-C assisted genome assembly using the software LACHESIS (https://github.com/shendurelab/LACHESIS) with the parameters, CLUSTER_N = 11, CLUSTER_MIN_RE_SITES = 583, CLUSTER_MAX_LINK_DENSITY = 9, CLUSTER_NONINFORMATIVE_RATIO = 0 (Burton et al., 2013).

3.5.1 Comparison with draft genome

The high-quality sequencing data was mapped to the draft celery genome by BWA software. The repeat data and no paired data were removed by SAMTOOLS (parameter: rmdup), and the high quality data was obtained (Etherington et al., 2015; Li et al., 2009). Meanwhile, we extracted the reads near cleavage site for assisted genome assembly. The

sample alignment rate reflected the similarity between sequencing data and reference genome.

3.5.2 Clustering

Short reads were compared to the draft genome, and the reads were compared to contigs or scaffolds. If reads pairs were captured by Hi-C on two contigs, an interaction between two contigs was inferred. The more reads that two contigs share, the stronger the interaction is, and the more likely they were grouped together. Contigs were clustered according to the interactions number, and chromosomes were then divided and inferred.

3.5.3 Sorting and Orientation

The positions of the strengths of each pair of two contig interactions and the interaction reads were sorted and oriented.

3.5.4 Assembly result statistics

Finally, a total of 3.047 Gb, accounting for 91.44% of the assembled celery genome, was anchored onto 11 chromosomes by Hi-C (Supplementary Table 12). A total of 3.047 Gb sequences, accounted for 91.44% of the genome, was anchored to the 11 celery chromosomes. The finally assembly genome is 3,332.58 Mb, and the scaffold N50 reached 289.78 Mb (Table 2). Grossly, we obtained a high-quality assembled celery genome. The N50 value is the largest among 32 representative plant species recently sequenced (Supplementary Table 13).

4. Genome prediction and annotation

4.1 Analysis process and method

4.1.1 Genome prediction

We conducted the gene structural prediction mainly based on homologous prediction, De novo prediction and other evidence-supported predictions. The homologous prediction is to compare protein sequence to a known homologous species with the genome sequence of a new species by Blast (*http://blast.ncbi. nlm.nih.gov/Blast.cgi*), Genewise (*http://www.ebi.ac.uk/~birney/wise2/*) and other software predicts gene structure(Birney et al., 2004; Camacho et al., 2009). Several software tools were used for prediction, such as Augustus (*http://bioinf.uni-greifswald.de/augustus/*), GlimmerHMM (*http://ccb.jhu.edu/software/glimmerhmm/*) (Stanke and Morgenstern, 2005), SNAP

(*http://homepage.mac.com/iankorf/*) (Korf, 2004). Other evidence supports predictions that use EST or cDNA data from homologous species by blat (*http://genome.ucsc.edu/cgi-bin/hgBlat*) (Kent, 2002). Combining the above prediction results, and integrated into one non-redundant and more complete gene set using IntegrationModeler (*EVM*, *http://evidencemodeler.sourceforge.net/*) integration software (Haas et al., 2008). Finally, combined the results of transcriptome, the EVM annotation were corrected by PASA (*http://pasa.sourceforge.net/*), and the usage method can be viewed at the website (*http://pasapipeline.github.io/*) (Haas et al., 2003).

4.1.2 Genome annotation

We conducted the genome annotation from the three aspects, including repetitive sequence annotation, gene annotation, and miRNA, et al annotation.

The method of repetitive sequence annotation can be divided into two types, homologous sequence alignment and de novo prediction. The homologous sequence alignment is based on a repeat sequence database (RepBase, *http://www.girinst.org/repbase*), using the Repeatmasker and Repeatproteinmask (*http://www.repeatmasker.org/*) (Bao et al., 2015; Tarailo-Graovac and Chen, 2009). The de novo prediction firstly constructed repeat sequence database using LTR_FINDER (*http://tlife.fudan.edu.cn/ltr_finder/*) (Xu and Wang, 2007), Piler (*http://www.drive5.com/piler/*) (Edgar and Myers, 2005), RepeatModeler (*http://www.repeatmasker.org/RepeatModeler.html*), RepeatScout (*http://www.repeatmasker.org/*) (Price et al., 2005), then predicted by Repeatmasker. For the other method to do de novo prediction, the TRF (*http://tandem.bu.edu/trf/trf.html*) program was used to detect tandem repeat in celery genome (Benson, 1999).

We conducted gene function annotation by using the known protein databases, such as SwissProt (*http://www.uniprot.org/*) (Bairoch, 2005), InterPro (*https://www.ebi.ac.uk/interpro/*) (Mulder and Apweiler, 2008), KEGG (*http://www.genome.jp/kegg/*) (Ogata et al., 1999), and TrEMBL (*http://www.uniprot.org/*) (Bairoch and Apweiler, 2000). RNA annotations mainly include tRNA, rRNA, miRNA, and snRNA. According to the structural characteristics of tRNA, tRNAscan-SE (*http://lowelab.ucsc.edu/tRNAscan-SE/*) was used to search tRNA (Chan and Lowe, 2019). Based ib the Rfam family's covariance model, INFERNAL

(*http://infernal.janelia.org/*) program was used to predict miRNAs and snRNAs (Nawrocki and Eddy, 2013). We select rRNA sequence of closely related species as a reference sequence to search rRNA by Blast.

4.2 Analysis results

4.2.1 Repeat sequence annotation

Repeat sequences mainly contain two categories, tandem repeat and interspersed repeat. The tandem repeat sequence includes a microsatellite sequence, and a small satellite sequence. The retrotransposon classes are LTR, LINE and SINE. Based on the *Denovo* repeat sequence prediction and the Repbase, the genome was subjected to repeat annotation, and showed that the genome contained 92.91% of the repeat sequence (Supplementary Table 14). Furthermore, we classified the TEs, and most of them belonged to LTR (85.75%) (Supplementary Table 15). Based on the alignment of genome with Repbase, we plotted the frequency of different types of repeats (Supplementary Figs. 2-3).

4.2.2 Tandem repeat analyses

Usually, repeat sequences were divided by that whether the repeat unit were clustered or not in a chromosome region, we defined that the clustered ones as the tandem repeats (TR), while the scattered ones dislocated in one whole chromosome were so-called transposons. The former ones can be divided into microsatelites, minisatelites, macrosatelites based on their repeat times. The latter ones can be grouped into more specific ones, like SINE, LINE and others (Gemayel et al., 2012; Mayer et al., 2010). In the celery genome, we detected 158.15Mb tandem repeat sequences using TRF, which accounted up to 4.75% of the whole genome (Supplementary Table 14).

According to the calculation of repeat type from single (mono-) to triple (tri-) repeat bases, we regarded the appearance of the repeat unit “A” or “C” as the Mononucleotide. Considering the complementary strand of “A” and “C” are “T” and “G”, separately, we totally unified the “A” or “T” as “A”, and took the “C” or “G” as “C”. Likely in the two repeat unit, Dinucleotide represent “AT” (including AT and TA), “GC” (including “GC” and “CG”), “AC” (including “AC”, “CA”, “TG” and “GT”) and “AG” (including “AG”, “GA”, “TC” and “CT”). With more repeat types, the repeat unit became more

complicated and we here only calculated the former three types from the "Mononucleotide" to "Trinucleotide".

With the calculation of tandem repeat sequences, we found the range of repeat unit from one single nucleotide to 2000 nucleotides, and drew the distributions of the repeat regions of tandem repeat and the density of different scale tandem repeats (Supplementary Table 16). We showed the distribution of the smaller tandem repeats with repeat units less than 10. We found the distribution of tandem repeat times were accompanied by the distribution of tandem density, and the peak of tandem density appeared at the repeat unit 4 with about 91 Kb/Mb, while the peak of tandem regions appeared at the repeat unit 2 with 62,905 regions (Supplementary Table 16; Supplementary Fig. 4a). We also studied the distribution of tandem repeat units less than 50bp and found both the peak of tandem repeat density and that of tandem repeat regions showed at the unit 21 with about 363Kb/Mb and 207,376 regions (Supplementary Table 16; Supplementary Fig. 4b). With all kinds of tandem repeats, the distribution of tandem repeat density and the tandem repeat regions are diverse when the repeat units are fewer than about 180 and 112 (Supplementary Table 16; Supplementary Fig. 5).

We specifically calculated the three types of tandem repeats, which mainly included the information about their repeat units, repeat regions, repeat copies, repeat bases, and also the bases within the limited regions (Supplementary Table 17). From the type of mononucleotide, the "A" ("A" or "T", with 2225 regions, 0.15Mb bases and the maximum region(s) including 217 units) apparently was dominant compared with the "C" ("C" or "G", with 20 regions, 0.75Kb bases and the maximum region containing 91 bases). Considering the dinucleotide, "AT" ("AT" and "TA") took the most in all calculation levels compared to other three sub-types "AC", "AG" and "GC", and "GC" ("GC" or "CG", with only one repeat region) was barely appeared. Repeat type with three nucleotides named trinucleotide, contained ten kinds of sub-types, within which "AAT" took the most percent in the trinucleotide type including about 5,980 repeat regions and 0.32 Mb repeat bases.

4.2.3 Centromeres and telomeres prediction

In this study, we predicted the centromeres and telomeres of celery based on previous research methods and the distribution of repeated sequences on chromosomes in

celery genome (Melters et al., 2013; Peska and Garcia, 2020; Somanathan and Baysdorfer, 2018).

Considering of the abundant tandem repeats within centromere region in most species, we delicately depicted their distributions along 11 celery chromosomes (Melters et al., 2013). Since the long repeat unit with limited repeat times probably covered the distribution of tandem repeats, we finally selected the tandem repeat unit less than 200bp as the subjects. Then, we calculated the percentage of tandem repeats within 1 Mb along the 11 chromosomes (Supplementary Fig. 6). Based on the distributions of celery tandem repeats, we deduced the putative centromeres marked with blue triangle, and calculated their potential physical position ranges and sizes (Supplementary Table S18). Most centromeres represented by the cluster of tandem repeats tend to be close to one end of the chromosome except Ag10. Based on the distribution of tandem repeats, only one notably peak was detected in chromosome 4 and 10, which clearly showed the centromere region. However, most of the putative centromere in the chromosomes, like chromosome 1, 2, 6, 8, and 11, confused by its multiple separated peaks of tandem repeat distributions. So it was difficult to clearly identify the centromere region, while we still selected most possible one as the putative centromere based on the higher percentages or more broad of the ranges (Supplementary Fig. 6, Supplementary Table S18).

The telomere sequences for each chromosome were identified using the sequence repeat finder (SERF) analysis platform (bioserf.org) (Somanathan and Baysdorfer, 2018). Both of two telomeres were predicted for 9 chromosomes, while only one telomere was detected in chromosomes Agr3 and Agr10 (Supplementary Table S18).

4.2.4 Gene structure annotation

We conducted *de novo* prediction of gene structure using Augustus, Genscan, GlimmerHMM, Geneid, and SNAP. The homologous species include *C. sativus*, *D. Carota*, *L. sativa*, and *A. thaliana*. A total of 31,326 genes were predicted in celery genome, and the support of each evidence for gene set were also shown (Supplementary Fig. 7; Supplementary Table 19). We further conducted the analyses of genes in celery and above mentioned species. Celery has fewer genes than Arabidopsis, coriander, carrot, and lettuce (Supplementary Table 19).

4.2.5 Gene annotation

The gene annotation was obtained by alignment of the known protein libraries, including KEGG, NR, InterPro, and Swiss-Prot databases (Fig. 1). Finally, a total of 29,050 (92.7%) genes in celery genome can be predicted to function. Among of them, 19,277 genes were annotated by four databases (Supplementary Tables 20-21).

4.2.6 rRNA, tRNA, snRNA, miRNA annotation

The rRNA, tRNA, snRNA, miRNA annotation of the celery genome obtained by comparison with known libraries or structural prediction (Supplementary Tables 22-23; Supplementary Figs. 8-9).

5. RNA-seq

5.1 Introduction

The samples of celery collected from 3 different tissues, including root, leaf, and petiole. Three celery varieties with 3 different colors' petiole, including green, white, and red were also used for RNA-Seq. Each sample was set as three replications. The RNA was isolated using RNA kit according to manufacturer's instructions.

5.2 Library construction and sequencing

5.2.1 RNA detection

(1) Agarose Gel Electrophoresis analyses RNA degradation and detect whether existing contamination.

(2) Nanodrop test the purity of RNA(OD260/280).

(3) Qubit accurately quantified RNA concentration.

(4) Agilent 2100 accurately detects RNA integrity.

5.2.2 Library construction

Using magnetic beads with Oligo (dT) to enrich the mRNA by base A-T pairing and the combination of mRNA ploy A tail, then, breaking mRNA into short fragments by adding fragmentation buffer, a single-strand cDNA was synthesized by random hexamers using mRNA as a template. The double-stranded cDNA was synthesized by adding buffer, DNA polymerase I, and dNTPs. We purified double-stranded cDNA using AMPure XP beads. Choosing the size of fragments using AM Pure XP beads after adding

tail A and connecting the sequencing linker, finally, PCR enrichment was performed to obtain the cDNA library.

5.2.3 Library inspection

We performed preliminary quantification by using Qubit 2.0, and the library was diluted until 1 ng/ul. Then, we detected the insert size of the library using Agilent 2100. Finally, we did accurate quantification for the effective concentration of the library (effective concentration >2 nM) using Q-PCR to ensure the quality.

5.2.4 Sequencing

We used HiSeq sequencing for the different libraries according to the effective concentration and target data volume.

5.3 Bioinformatics analysis

5.3.1 Original sequences data

The original image data files were obtained by Illumina HiSeq^{TM} transformed the original sequencing sequences by CASAVA Base Calling. We called it Raw Data or Raw Reads, and the results were stored in FASTQ format.

5.3.2 Data quality assessment

5.3.2.1 Check the distribution of sequence error rate

Error rate of each base sequencing was obtained by Phred score (Qphred= -10log10(e)). Phred value was obtained by a rate model during base calling process.

5.3.2.2 Check A/T/G/C content

The GC content distribution was used to detect the phenomenon whether there exists the separation between AT and GC.

5.3.2.3 Sequencing data filtering

The original sequencing sequence from sequencing contained low-quality reads with connectors. In order to ensure the quality of information analysis, we filtered the raw reads to gain clean reads.

6. Comparative genomic analyses

6.1 Materials and Methods

6.1.1 Gene family analysis

OrthoFinder (http://orthomcl.org/orthomcl/) was used for the single-copy gene and multi-copy gene family identification in the celery and other 6 species (Supplementary Fig. 10; Supplementary Table 24) (Emms and Kelly, 2019). The Pfam database (http://pfam.sanger.ac.uk) was used to identify all the transcription factors (TFs) with the e-value <$1e^{-4}$. Then, a home-made Perl script was used to extract the specific TFs gene family from the result of Pfam program. For example, we extracted the NBS family genes with Pfam number PF00931.

6.1.2 Phylogenetic tree construction and divergence time estimation

Firstly, we performed multiple sequence alignments on all single-copy genes using MAFFT software (Katoh and Standley, 2013). Then, we combined all the alignment results to construct a phylogenetic tree called super alignment matrix. Here, we performed the construction of 7 species phylogenetic trees by maximum likelihood method (ML tree) using RAxML software (Stamatakis, 2014). We used 422 single-copy gene families to estimate divergence time using Mcmctree in PAML software (Yang, 2007). The time correction points were obtained from TimeTree website (http://www.timetree.org) (Kumar et al., 2017). The followed time points were used for the time estimate correction, including Arabidopsis and grape (107-135 Mya), Arabidopsis and lettuce (111-131 Mya), lettuce and ginseng (77.3-91.7 Mya), ginseng and carrot (45-70 Mya), carrot and coriander (22-37 Mya). The operating parameters of Mcmctree were set as burn-in = 5,000,000, sample-number = 1,000,000, and sample-frequency = 50.

6.1.3 Inference of gene colinearity, Ks calculation, distribution fitting, and correction

Colinear genes were inferred using ColinearScan (Supplementary Fig. 11) (Wang et al., 2006). Firstly, BlastP searches were performed to find putative homologous genes within a genome or between genomes. When running ColinearScan, maximal gap length between neighboring genes in colinearity along a chromosome sequence was set to 50 genes according to previous reports (Wang et al., 2017a; Wang et al., 2016a; Wang et al., 2005; Wang et al., 2015). Since large gene families lead to difficulty to infer gene colinearity, families with > 30 genes were removed before running ColinearScan.

Secondly, to see directly the homology within and between genomes, homologous gene dotplots were produced using MCScanX toolket (Wang et al., 2012). Dotplots were used to facilitate identification of homologous blocks produced by different polyploidization events (Supplementary Fig. 12). Ks values were estimated between colinear homologous genes, by using the YN00 program in the PAML (v4.9h) package with the Nei-Gojobori approach (Yang, 2007), and the median Ks of colinear homologs in each block was shown in the constructed dotplots to help group blocks produced by different events. This would found paralogous blocks and genes produced by each WGT or WGDs in each Apiaceae plants, and orthologous genes between different plants. With each grape chromosome, its 4X duplicated celery regions were inferred, and pinched into four sets of pseudo-chromosomes by checking whether two blocks were neighboring to one another as to the reference chromosome (Supplementary Fig. 13). Each set of reconstructed pseudo-chromosomes is assumed to form the corresponding subgenome produced by the recursive polyploidizations. Similar is with each of the other Apiaceae plants. Taken celery as an example, the (colinear) paralogs produced by each WGT or WGDs were used to infer the evolutionary dates of the related events; and the celery-coriander (colinear) orthologs were used to date their divergence.

Thirdly, the probability density distribution curve for Ks was estimated by MATLAB with the kernel smoothing density function (ksdensity, bandwidth was set to 0.025, typical value). Then, multi-peak fitting of the curve was performed using the Gaussian approximation function in the curve fitting toolbox cftool within MATLAB. The coefficient of determination (R-squared) was required to be at least 0.95 (Supplementary Fig. 14).

Fourthly, in that we have diverged evolutionary rates among Apiaceae plants and others, to have a common evolutionary rate to perform a reasonable dating, we performed a correction of evolutionary rates (Supplementary Figs. 14,15). Here, different from previous practice (Wang et al., 2017b; Wang et al., 2016c), we performed a two-step rate correction. Based on the fact that celery, carrot, and coriander shared two extra polyploidizations after the split with lettuce, and the different evolutionary rates of these two polyploidizations, we conducted two rounds of rate correction. In the first step, we managed to correct evolutionary rate by aligning the Ks distributions of celery, coriander,

lettuce and carrot γ duplicates to that of grape γ duplicates, which have the smallest Ks values. Then, according to the result that celery with the slower rate during both the two extra polyploidizations, we re-corrected the evolutionary rates of celery α produced duplicates with coriander as the reference. The follows as details.

We estimated the evolutionary rates of γ-produced duplicated genes, corrected according to our report (Wang et al., 2019). The maximum likelihood estimated μ from inferred Ks median of γ-produced duplicated genes were aligned to have the same value of those of grape. Supposing a grape duplicated gene pair to have a Ks value that is a random variable, and for a duplicated gene pair in another genome the Ks to be $X_i \sim \left(\mu_i,\ \sigma_i^2\right)$.

We also performed the Ks correction analysis to distinguish the order of each polyploidization events with the method applied in previous study(Wang et al., 2015). Supposing that Ks values in the other two genomes i, j to be $X_{i-j} : N(\mu_{i-j}, \sigma^2_{i-j})$, and that the ratio of the evolutionary rate of species i to common evolutionary rate of angiosperms genus is r_i, the correction coefficient λ_i that corrects it to the rate of co-evolutionary rate is equal to $\lambda_i = \frac{1}{r_i}$, and the correction coefficient factor is $\lambda_{ij} = \lambda_i \cdot \lambda_j$.

To get the corrected $X_{i-j-correction}$, Then

$$\mu_{i-j-correction} = \mu_{i-j} \cdot \lambda_i \cdot \lambda_j$$

Due to

$$E[tX] = tE[X],\ D[X] = t^2 D[X]$$

then,

$$X_{i-j-correction} : N(\mu_{i-j-correction}, \sigma^2_{i-j-correction}) = N(\lambda_i \lambda_j \mu_{i-j}, \lambda_i^2 \lambda_j^2 \sigma^2_{i-j})$$

Other genomes among involved plants diverge from grape is close to the same time. For the genome i, then

$$X_G \sim \left(\mu_G,\ \sigma_G^2\right) \quad \mu_{Vv-Ls-correction} = \mu_{Vv-Ag-correction} = \mu_{Vv-Cs-correction} = \mu_{Vv-Dc-correction}$$

$$\frac{\mu_{Vv-i-correction}}{\mu_{Vv-Cs-correction}} = \frac{\mu_{Vv-i}\cdot\lambda_{Vv}\cdot\lambda_{i}}{\mu_{Vv-Cs}\cdot\lambda_{Vv}\cdot\lambda_{Cs}} = \frac{\mu_{Vv-i}\cdot\lambda_{i}}{\mu_{Vv-Cs}\cdot\lambda_{Cs}}$$

$$\mu_{Ls-Ag-correction} = \mu_{Ls-Cs-correction} = \mu_{Ls-Dc-correction}$$

$$\frac{\mu_{Ls-i-correction}}{\mu_{Ls-Cs-correction}} = \frac{\mu_{Ls-i}\cdot\lambda_{Ls}\cdot\lambda_{i}}{\mu_{Ls-Cs}\cdot\lambda_{Ls}\cdot\lambda_{Cs}} = \frac{\mu_{Ls-i}\cdot\lambda_{i}}{\mu_{Ls-Cs}\cdot\lambda_{Cs}}$$

After its divergence from the other studied plants, grape has not been affected by polyploidization any more, we assumed that the evolutionary rate of grape genes is relatively stable and, therefore, set $\lambda_{Vv} = 1$.

$$\frac{\lambda_i}{\lambda_{Cs}} = a_i = mean\left\{\frac{\mu_{Vv-Cs}}{\mu_{Vv-i}}, \frac{\mu_{Ls-Cs}}{\mu_{Ls-i}}\right\}$$

Finally, for each species i, the correction coefficient ratio should be calculated by $\lambda_i = \lambda_{Vv}\cdot a_i$, and all the Ks distributions were corrected by the correction coefficient ratio of each species.

Specially, due to the rapid evolution rate of goldfish and rice, it requires multiple corrections, and the recent doubling event has not been corrected again.

After correction, the Ks peak for ω is basically similar, however, the ks peak for α has significant deviations. It shows that the rate of evolution of carrots, coriander, and celery is significantly different after the most recent divergence. Based on this, we have re-corrected the time for α. Because coriander slower evolutionary rate, let $\lambda_{Cs-\text{Apiaceae}} = 1$.

Then,

$$\frac{\mu_{Ag-Ag-\text{Apiaceae}-correction}}{\mu_{Cs-Cs-\text{Apiaceae}-correction}} = \frac{\mu_{Ag-Ag-\text{Apiaceae}}\cdot\lambda_{Ag-\text{Apiaceae}}\cdot\lambda_{Ag-\text{Apiaceae}}}{\mu_{Cs-Cs-\text{Apiaceae}}\cdot\lambda_{Cs-\text{Apiaceae}}\cdot\lambda_{Cs-\text{Apiaceae}}} = \frac{\mu_{Ag-Ag-\text{Apiaceae}}}{\mu_{Cs-Cs-\text{Apiaceae}}}\lambda^2_{Ag-\text{Apiaceae}}$$

$$\lambda_{Ag-\text{Apiaceae}} = \sqrt{\frac{\mu_{Cs-Cs-\text{Apiaceae}}}{\mu_{Ag-Ag-\text{Apiaceae}}}}$$

Eventually, to construct the table with the grape genome as a reference, all grape genes were listed in the first column. Each grape gene may have two additional colinear genes in its genome due to WGT event, and two other columns in the table listed this information. For a grape gene, when there was a corresponding colinear gene in an expected location, a gene ID was filled in a cell of the corresponding column in the table.

When it was missing, often due to gene loss or translocation in the genome, the cell contained a dot. For the lettuce genome, with whole-genome triplication (WGT), we assigned three columns. For the carrot, coriander or celery genome, each affected by two paleo-polyploidization events, we assigned four columns. Therefore, the table had 48 columns, reflecting layers of tripled and then fourfold homology due to recursive polyploidies across the genomes.

6.1.4 Reconstruction of ancestral karyotypes of Apiales plants

The colinearity of compared genomes could reflect the karyotype change and even to uncover the trajectories of the formations of their ancestors. Based on the homologous dot-plots, we selected the four compared genomes presented in the phylogenetic locations and deduced their ancestral chromosomes at the important evolutionary periods, eg. before the divergent nodes and the periods before or after different polyploidizations. With the potential existent theory showed in the dotplots of two compared genomes, the extant chromosomes came from the interaction of ancestral chromosomes, which usually include the following cases, the "crossover" appeared in the arms of two interacted chromosomes, the "end to end joint" appeared in the end of chromosomes' arms, also "nested chromosome fusion" showed in one chromosome inserted into another one completely. Most extant chromosome suffered more than one kind of interaction within their evolutionary history, especially after once or more rounds of polyploidizations.

6.2 Results

6.2.1 Gene colinearity within and among genomes

Homologous colinearity of existing genomes is an important clue to reveal the evolution of complex genomes. Using ColinearScan (Wang et al., 2006), we inferred colinear genes within and between celery and other reference genomes, which provides a function for evaluating the statistical significance of blocks of colinear genes (Supplementary Table 25). For the blocks with four or more colinear genes, we found 22,433 duplicated genes pairs in celery. For the colinear regions containing more than 10 gene pairs, celery (9,834 pairs reside in 394 blocks) has larger number than grape, which has 7,275 pairs residing in 286 blocks (Supplementary Table 25).

In addition, we indicated that the colinearity between genomes is much better than within each genome (Supplementary Table 25). For example, there were only 117, 108,

and 166 colinear gene pairs residing in the longest duplicated blocks in celery, coriander, and carrot, respectively. However, 864 and 794 colinear gene pairs reside in longest duplicated block between celery and coriander, celery and carrot, respectively (Supplementary Tables 25-30).

6.2.2 Two paleo-polyploidization events

By constructing the homologous dotplot between genomes (Supplementary Figs. 11-12), and comparing the homologous chromosome regions of celery, coriander, carrot, lettuce, and grape, we found that after the differentiation of celery and lettuce, two consecutive whole-genome duplication (WGD) events occurred in the ancestral Apiaceae genome.

We characterized the synonymous substitution divergence (Ks) between each colinear gene pair, which showed a clear bimodal structure with two distinct sets in celery, one with Ks distribution peaking at about 0.58 and another peaking at 1.03 (Fig. 2), indicating at least two large-scale genomic duplication events, named as Apiaceae α and ω events, respectively (Supplementary Fig. 15; Supplementary Table 31). We also inferred colinear genes and characterized Ks distribution in other plant genomes. The peaks with larger Ks values in all grape, lettuce, coriander, and carrot genomes correspond to the γ, as repeatedly reported previously (Jaillon et al., 2007; Paterson et al., 2012; Wang et al., 2016b).

To date the WGT event in the celery lineage, we performed evolutionary rate correction to the evolutionary rates (Supplementary Fig. 15; Supplementary Table 32). Here, different from previous practices (Wang et al., 2017b; Wang et al., 2016c), we performed a two-step rate correction. Based on the fact that celery, carrot, and coriander shared two extra polyploidizations after the split with lettuce, and the different evolutionary rates of these two polyploidizations, we conducted two rounds of rate correction. In the first step, we managed to correct evolutionary rate by aligning the Ks distributions of celery, coriander, lettuce and carrot γ duplicates to that of grape γ duplicates, which have the smallest Ks values. Then, according to the result that celery with the slower rate during both the two extra polyploidizations, we re-corrected the evolutionary rates of celery α produced duplicates with coriander as the reference.

Eventually, we inferred that the celery paralogs had a corrected Ks distribution peaking at 0.36 for α event and 0.71 for ω event. Assuming that the γ occurred 115–130 Mya with Ks distribution peaking at 1.256(Jiao et al., 2012; Vekemans et al., 2002), these two events have occurred 34-38, 66-77 Mya. Notably, the lettuce WGT-produced paralogs had a corrected Ks distribution peaking at 0.64 (59-66Mya), showing that the Asteraceae-common WGT event was between the two paleo-polyploidizations events of Apiaceae. In addition, the celery-coriander and celery-carrot splits were inferred to have occurred 11–13 Mya, 20-22 Mya, respectively (Fig. 2). The estimated time was consistent with estimation by MCMCtree in PAML software (Supplementary Fig. 16). The Apiaceae species split from lettuce at 82-93 Mya (Fig. 2; Supplementary Fig. 15).

6.2.3 Multiple alignment

With the grape genome as a reference, we produced a table to store inter- and intra-genomic homology information (Supplementary Tables 26-30). First, we filled in all grape gene IDs in the first column of the table, then added gene IDs from celery and other genome column by column, species by species according to the colinearity inferred by above alignments. As noted above, if no gene lost, a grape gene would have 3 orthologous genes in lettuce, and 4 in each of an Apiaceae plant (celery, coriander, and carrot) genome. When a species contained a gene showing colinearity with a grape gene, a gene ID was filled into an appropriate cell in the table. When a species did not have an expected colinear gene, often due to gene loss, translocation or insufficient assembly, a dot (signifying missing) was filled into the appropriate cell. For grape, lettuce, carrot, coriander, and celery there were allocated 16 (1+3+4x3) columns in the table. Moreover, due to their shared the WGT (γ), each chromosomal segment would repeat three times in each genome. Based on homology inferred in grape, we therefore extended the table to 48 columns (Supplementary Fig. 13). Eventually, we constructed a table of celery and other plant genes reflecting three polyploidizations and all salient speciation. In summary, the table summarized results of multiple-genome and event-related alignment, reflecting layers of tripled and/or doubled homology due to recursive polyploidizations.

6.2.4 Genomic fractionation

We analyzed celery gene loss rates by referring to the grape, coriander, carrot, and grape genomes. Using the grape as the reference, celery gene loss rates as to different

grape chromosomes varied from 54% (grape chromosomes 8) to 80% (grape chromosomes 9) (Supplementary Tables 33-34; Supplementary Fig. 17a). Using the carrot as the reference, celery gene loss rates varied from 42% (carrot chromosomes 6) to 57% (carrot chromosomes 9) (Supplementary Tables 33-34; Supplementary Fig. 17b). Using the coriander as the reference, celery gene loss rates varied from 43% (coriander chromosome 3) to 58% (coriander chromosome 11) (Supplementary Tables 33-34; Supplementary Fig. 17c).

Furthermore, the observed gene loss numbers were fitted by using different density curves of geometry distribution (Supplementary Fig. 18). The F-test was performed, and the P-value were 0.944, 0.939, and 0.892 for celery as compared with carrot, coriander, and grape, respectively (Supplementary Table 35). The retention of duplicated genes reside in celery was detected using the grape, coriander, and carrot as references, respectively (Supplementary Fig. 18).

6.2.5 RNA-seq analyses

6.2.5.1 Summary of sequencing data quality

The clean data of 3 tissues (root, leaf, petiole) of celery totally produced 74.02 Gb data (Supplementary Table 36). The clean data of 3 different colors (green, white, and red) of celery were 66.18 Gb (Supplementary Table 37).

6.2.5.2 Alignment analysis

We used the software HISAT to perform genomic positioning analysis for the filtered sequences(Kim et al., 2015). The total mapped rates of 3 tissues were more than 95%, and the uniquely mapped rates were more than 90% (Supplementary Table 38). Similar, there was the same trends for the 3 different stem-colored celery (Supplementary Table 39).

6.2.5.3 Gene expression analysis

We adopted the HTSeq to analysis the gene expression level(Anders et al., 2015). In order to make the different genes and different experiments comparable, FPKM (Fragments Per Kilobase of transcript sequence per Millions base pairs) was used to estimate gene expression levels(Trapnell et al., 2010), which took into account the effect of sequencing depth and gene length (Supplementary Tables 40-41). In general, the

FPKM value of 0.1 or 1 was used as thresholds for judging whether or not a gene is expressed. We compared gene expression levels under different conditions by FPKM.

6.2.5.4 RNA-seq quality assessment

The correlation of gene expression between samples is an important indicator to test the accuracy of the experiment. The closer the correlation coefficient is to 1, the higher the similarity in expression patterns between samples. We required that the biological repeat sample relative coefficient R^2 to be at least greater than 0.8 (Supplementary Fig. 19).

6.2.5.5 Differentially expressed genes (DEGs)

The differential expression analysis was mainly divided into the following three parts.

1) Normalize the readcount;
2) Calculating the hypothesis test probability (p-value);
3) Multiple hypothesis test calibration was performed to obtain the FDR value. We used the DESeq program to conduct DEGs analyses with padj<0.05(Anders and Huber, 2010).

6.2.5.6 Differential expressed genes analysis

The FPKM values of DEGs under different experimental conditions were used for hierarchical clustering analysis (Supplementary Fig. 20). Different colors represented different clustering group. The gene expression patterns in the same group were similar, and may participate in the similar biological process. The common or specific DEGs among different tissues or different celery varieties with different stem colors were shown by venn diagrams (Supplementary Fig. 21). We conducted the GO enrichment analyses of DEGs between any two tissues of celery or between any two varieties of celery (Supplementary Figs. 22-23). In addition, we conducted the KEGG enrichment analyses of DEGs between any two tissues of celery or between any two varieties of celery (Supplementary Figs. 24-25).

6.2.6 Gene expression balance analyses

We conducted the gene expression bias analyses using the RNA-Seq of 3 tissues (root, petiole, and leaf) and 3 varieties (different-colored petioles, including green, red and white) of celery (Supplementary Tables 36-41). Homoeologous regions produced by celery were grouped in subgenome A1-A4 as to the mapped grape chromosomes. Here, the higher

expression means that the gene expression in one subgenome was more than twice of the mean of gene expression in other 3 subgenomes. The lower expression means that the gene expression in one subgenome was less than twice of the mean of gene expression in other 3 subgenomes. Approximately balanced gene expression was observed between duplicated copies of chromosomes produced in ω and Apiaceae α.

Among all 4 subgenomes using grape as reference, 1.08%-1.71% duplicated genes showed a clear higher expression, 11.31%-13.35% duplicated genes showed a clear lower expression, and 85.44%-87.62% duplicated genes showed no significant difference in the celery root gene expression (Supplementary Fig. 26a; Supplementary Table 42). A total of 1.1%-1.63% duplicated genes showed a clear higher expression, 11.14%-13.0% duplicated genes showed a clear lower expression, and 85.86%-87.24% duplicated genes showed no significant difference in the celery petiole gene expression (Supplementary Fig. 26b; Supplementary Table 42). A total of 0.86%-1.44% duplicated genes showed a clear higher expression, 10.93%-12.81 % duplicated genes showed a clear lower expression, and 86.0%-87.63% duplicated genes showed no significant difference in the celery leaf gene expression (Supplementary Fig. 26c; Supplementary Table 42). A total of 1.21%-1.77% duplicated genes showed a clear higher expression, 10.99%-13.17% duplicated genes showed a clear lower expression, and 85.62%-87.21% duplicated genes showed no significant difference in the white variety of celery gene expression (Supplementary Fig. 26d; Supplementary Table 42). A total of 0.97%-1.70% duplicated genes showed a clear higher expression, 10.69%-12.57% duplicated genes showed a clear lower expression, and 86.46%-87.62% duplicated genes showed no significant difference in the red variety of celery gene expression (Supplementary Fig. 26e; Supplementary Table 42). A total of 0.93%-1.66% duplicated genes showed a clear higher expression, 10.28%-12.03% duplicated genes showed a clear lower expression, and 86.81%-88.06% duplicated genes showed no significant difference in the green variety of celery gene expression (Supplementary Fig. 26f; Supplementary Table 42).

6.2.7 Celery chromosomes representing the Apiaceae proto-chromosomes

We reconstructed the Apiaceae proto-chromosomes and their evolutionary trajectories to extant chromosomes (Fig. 3). Actually, we found that the Apiaceae proto-chromosomes could be represented by the celery chromosomes.

Using homologous gene dotplots, we characterized the correspondence between genomes of Apiaceae plants and grape (Supplementary Fig. 12). The undisturbed integrity of celery chromosomes Ag1-5 and Ag8 could be evidenced by each of them having complete correspondence to one of carrot chromosomes (Supplementary Fig. 12a). Therefore, they could be used to represent the Apiaceae proto-chromosomes, at least with the information so far.

The proto-integrity of the other celery chromosomes is supported by homology with grape chromosomes (Fig. 3a; Supplementary Fig. 12b). Taking celery chromosome Ag10 as an example, ignoring permuted correspondence due to reciprocal DNA inversions, to its ~3/4 length Ag10 shared orthology with grape Vv13, at the meantime paralogous to Vv6 and Vv8 due to the γ WGT (Supplementary Fig. 12b). In contrast, the same Ag10 region corresponds to different regions in Dc3, Dc4, and Dc6 (Supplementary Fig. 12a). These showed that the Ag10 most likely preserved much the proto-chromosome structure, while the Dc3, Dc4, and Dc6 were reconstructed chromosomes after their split. The remaining part of Ag10, merged from Vv16 (Supplementary Fig. 12b), was shared with the other Apiaceae (Supplementary Fig. 12c-e). Putting together, Ag10 could represent an Apiaceae proto-chromosome.

Formation of carrot chromosomes. Continuingly exploiting the orthologous correspondence between genomes, we managed to reconstruct the ancestral karyotypes on key evolutionary nodes and evolutionary trajectories to produce extant chromosomes (Fig. 3a). Firstly, starting from the 11 Apiaceae proto-chromosomes, renamed as R1-11, corresponding to Ag1-9 orderly, we inferred how the carrot and coriander chromosomes formed. We found that Dc7-9 preserved the integrity of proto-chromosomes, R1, R5, and R8, ignoring some intra-chromosome inversions. The other five carrot chromosomes were each reconstructed after its split from the other Apiaceae plants. Specifically, a crossing-over between R6 and R11 produced Dc2 and an intermediate chromosome D6I (Fig. 3b). Intermediate chromosomes are only tentatively named to show their existence in the extant chromosome. R9 has orthology in Dc1 and Dc6, while Dc1 or Dc6 has orthology to more celery chromosomes. Considering Dc6 was a reconstructed chromosome after their split, most likely Dc1 is also a reconstructed chromosome in the carrot. Similar the other carrot chromosomes, Dc2 and Dc5, seemed reconstructed.

Specifically, a crossing-over between R6 and R11 produced Dc2 and an intermediate chromosome tentatively named as D6I, which then sequentially crossed-over with R7 and R9 to produce two intermediates Dc6II and D1I. D1I crossed-over with R9 to produce D1 and an intermediate D6III. D6III and D6II joined end to end to produce D6IV and a satellite chromosome S1. D6IV and R10 crossed to produced D6 and D3II, with D3II crossed over with R3 to produce D4 and an intermediate D3III. D3III joined end to end with D3I, which was produced by a crossing-over between R2 and R4 to form D5. During the end-end joining, D3 and a satellite chromosome S2 was produced. Grossly, during the formation of carrot chromosomes, two putative satellite or B chromosomes (S1-2), each formed mainly two telomeres, might have produced but lost, resulting in chromosome number reduction.

Formation of coriander chromosomes. The trajectories to form coriander chromosomes were showed in Fig. 3. By checking carrot and celery chromosome orthology, we inferred the Apiaceae proto-chromosomes R1-10 (Ag1-10). C5 and C7 were completely succeeded from their ancestral chromosomes R8 and R5. With the homologous gene dotplot between coriander and celery, we managed to deduce the formation of the other extant 9 coriander chromosomes (Fig. 3c; Supplementary Fig. 12). R4 and R11 crossed-over to produce two intermediate of C1I and C10I. C10I then crossed over with R1 to produce to C9I and C3I. C9I crossed over R6 to produce C9 and C11. C3I crossed over with a mediate C3II, which was by-produced in the formation process C4 by the crossover between R3 and R7, to produce C3III and C10II. C10II crossed over R9 to generate C10III and C6I. C6I and C3III crossed over to produce C6 and an intermediate C3IV. C3IV crossed over C8I, which was generated by the cross-over between R2 and R10 to produce C2, to generate C8 and C3. C10III combined with C1I to form C10 and C1.

The Apiaceae proto-chromosomes R1-10 were compared to grape chromosomes to reconstruct karyotypes before and after ω and Apiaceae-α polyploidizations (Fig. 3a). Nineteen grape chromosomes could be used to reconstruct 21 proto-chromosomes of early eudicot plants (A1-A7; B1-B7; C1-C7), tripled from seven pre-ECH proto-chromosomes: E1-E7 (Fig. 3a). Repetitive co-occurrence of the 21 post-ECH chromosomes (represented by grape chromosomes) in the celery chromosomes permitted deductions about the timing

of rearrangements. That is, if two or more grape chromosomes showed corresponding homology four times to celery chromosomes, they most likely had merged before the ω (Fig. 3d). In contrast, if two or more grape chromosomes showed corresponding homology only two times in celery chromosomes, they most likely had merged after the ω but before the Apiaceae-α. For example, the post-ECH chromosomes A5, A1, and A2 coincided in each of Ag1, Ag5, Ag6, and Ag8, which could be explained by their fusion into a proto-chromosome P1 before the ω (Fig. 3d). A segment of A5 unexpectedly appearing in Ag9 but not in Ag6 as part of a P1 duplicate could be explained by accidental crossing-over between the P1 duplicate and a P5 duplicate, mainly formed by A6 and the part of B5 (Fig. 3e,f). In contrast, A7 appeared twice in homologies with Ag5 (or R5) and Ag8 (or R8), but not in Ag1 (or R1) or Ag6 (or R6), which implied that after ω as part of another proto-chromosome P7, A7 fused with P1, and formed a relatively recent chromosome Q2 before Apiaceae-α (Fig. 3d,f). After the Apiaceae-α, Q2 duplicated to produce Q2a and Q2b, with the former crossing-over with an intermediate chromosome R4I to produce R4, and with the latter crossing-over with Q9b (formed by steps of fusion or crossing-over) to make R3 (Ag8) and R8 (Ag8) (Fig. 3g).

By checking the homologous dotplot between grape and celery, we managed to deduce the karyotype and proto-chromosome formation before the Apiales whole-genome duplication. Actually, we inferred 8 chromosomes at node P, and found 14 step of changes along with their formation. The core eudicot had 21 chromosomes at node H after the whole-genome triplication shared by major eudicots, originated from the ancestral 7 haploid chromosomes at node E (Fig. 3e). After then, Apiales underwent a polyploidization closely and its ancestral genome reorganization significantly from the dotplot between grape and its extant genome (Supplementary Fig. 12e; Supplementary Table 43), from which we could traced back to the details of the formation of the ancestral chromosomes at different significant evolutionary nodes and we finally got their trajectories of its formation of their karyotypes (Fig. 3e-g). During the trajectory from node H to P, the reorganization within this period mainly included 13 times of “end to end joint” signed with “EJ” and two times of crossover signed with a cross and an arrow. A1 and A5 jointed from end to end and formed into the mediate chromosome P1I at step one, which then jointed to A2 triplicated from E2 and got the P1 at the node P at step two.

Likely, C3 and C7 also jointed into one mediate and then jointed C2 got an P6VI at step three, which would be used to combined another mediate chromosome and finally formed P6 at node P at step four and eight. Continually, C6 and C5 interacted into two mediate chromosomes (P6I and P6II) and separately attended into two breaches at step five, within which the latter one then jointed with C4 and formed another mediate P6III. While P6I jointed another mediate P2I originated from the crossover between B7 and A3, and they then jointed into P2 at step 8. The by-produced P5I finally jointed with the former P6III and got P6IV, which then joined with B6 and P6VI and finally formed its P6 at step eleven. B3 successively jointed B1, C1 and B4 after three times of end to end joint, and finally formed P3. The left P5 was simply formed by the joint between A6 and B5.

Then, during the process from node P to node Q, the ancestral genome changed from 8 to 10 and fairly included 6 times of end to end joint during its 8 main steps of reorganizations (Fig. 3f). Likely, we deduced the trajectory from the homologous dotplot between grape and celery, and the homologous blocks showed the clues to reflect their shared homologous parts within Apiaceae-α or ω. After ω, the ancestral chromosome doubled into "a" and "b" right after. From the trajectory from node P to Q, P1a jointed P2a and P8a and formed into Q1 at node Q along with step one and step two. Likely, Q2, Q9, Q10 and Q 3 all generated from two ancestral chromosomes simply end to end joint, while the left ones just completely inherited from their ancestral chromosomes (Fig. 3f).

The trajectory from node Q with 10 chromosomes to R with 11 chromosomes was exclusively depicted in Fig. 3g. We totally deduced 19 steps of reorganizations and mainly included 13 times of crossover and 5 times of end to end joint signed with "EJ". Followed with the former trajectory from node P to Q, the genome doubled its chromosomes signed with "a" and "b" after Apiaceae-α. At step one in the trajectory, Q9A and Q10a interacted with each other and formed into R2 and a mediate R4I, which lately got crossover with Q2a and formed into R4 at node R and another mediate R5I. R5I then jointed with Q8a and Q4a and generated R5 at node R along with step three to six. Likely, the following trajectories of the formation of each chromosome were showed at Fig. 3g along with the left steps, and finally formed the genomes appeared at node R.

Eventually, we inferred the formation from ECH chromosomes of 8 P chromosomes before the ω, about 10 Q chromosomes after the diploidization following ω and before the

Apiaceae-α, and about 11 R chromosomes after diploidization following the Apiaceae-α that formed the extant Apiaceae chromosomes (Fig. 3a).

6.2.8 Comparative analyses of transcription factor families

A total of 2,090 transcription factors (TFs) genes were identified in the celery genome, and classified into 62 families (Fig. 4; Supplementary Table 44). MYB gene family (240) was the largest among all predicted TF families in celery, followed by bHLH (131) and AP2/ERF (129) gene families, and they were mainly involved in resisting stress, growth, and development in plants. Comparatively, we identified 2,186, 2,102, 2,632, 4,111, 2,330, and 2908 TF genes in grape, Arabidopsis, lettuce, ginseng, carrot, and coriander genomes, respectively classified into 63, 63, 61, 60, 61, and 63 families (Supplementary Table 44). There were 6, 5, and 3 large families with the gene number more than 100 in coriander, carrot, and celery genome, respectively (Supplementary Fig. 27,28).

After performing normalization, we found that the fold change of 6, 1, 3, 1, and 3 gene families were larger than 2 in celery as compared to grape, Arabidopsis, lettuce, ginseng, and carrot, respectively (Fig. 4a; Supplementary Table 44). In addition, the fold change of 2, 4, 3, 1, 4, and 4 gene families were less than 0.5 in celery compared to the grape, Arabidopsis, lettuce, ginseng, carrot, and coriander, respectively (Fig. 4a; Supplementary Table 44). The fold change of some gene families, such as nucleotide-binding (NBS), was less than 0.5 in celery compared with grape, Arabidopsis, lettuce, carrot, and coriander. The fold change of growth-regulating factors (GRF) gene family, was less than 0.5 in celery compared with lettuce, carrot, and coriander, while more than twice in celery compared with grape, Arabidopsis, and ginseng. The fold change of far-red-impaired response (FAR1) gene family, was less than 0.5 in celery compared with grape and ginseng, while more than twice in celery compared with lettuce and carrot. The fold change of signal transducer and activator of transcription (STAT), was less than 0.5 in celery compared with Arabidopsis, lettuce, carrot, and coriander.

To further understand the expansion and contraction of these gene families in the evolution, we constructed the phylogenetic trees using protein sequences of these genes. We found FAR1 gene family significantly expanded in ginseng, and accounted for 59% of all FAR1 genes in these 7 species (Supplementary Fig. 29). However, the GRF gene

family significantly contracted in grape. The number of GRF genes was 3, 5, 105, 14, 55, 98, and 21 in grape, Arabidopsis, lettuce, ginseng, carrot, coriander, and celery, respectively (Supplementary Table 44; Supplementary Fig. 29).

In addition, we conducted expression analyses of these gene families in celery using RNA-seq datasets, including 3 tissues (root, petiole, leaf) and 3 varieties with different-colored petiole (green, white, red). We found that some genes showed different expression patterns in these tissues or varieties although they belonged to the same gene family. Interestingly, we found that most (90.47%) GRF family genes of celery were not expressed in these tissues or varieties (Supplementary Fig. 30).

NBS gene family

Celery has the fewest NBS disease resistance genes. The number of NBS genes was 442, 166, 392, 215, 148, 189, and 62 in grape, Arabidopsis, lettuce, ginseng, carrot, coriander, and celery, respectively (Fig. 4; Supplementary Tables 44-47; Supplementary Figs. 31-33). The NBS family genes were mainly classified into 3 groups, TIR-NB-ARC-LRR (TNL), CC-NB-ARC-LRR (CNL), and RPW8-NB-ARC-LRR (RNL) type, and most genes grouped into the former two groups. In celery, there were 10, 44, and 8 NBS genes in TNL, CNL, and RNL types, respectively (Supplementary Table 45). There were more genes for CNL type than TNL type in celery, coriander, carrot, ginseng, and grape. However, it is reverse in both lettuce and Arabidopsis.

GRF gene family

The Growth-regulating factor (GRF) family is a plant-specific transcription factors, which contains two highly conserved protein domains, WRC (Trp–Arg–Cys) and QLQ (Gln–Leu–Gln) (Rodriguez et al., 2016). GRFs are identified for their roles in stem and leaf development, flower and seed formation, anthers development, root development, reproductive development, senescence, and developmental plasticity in response to external cues (Kim and Tsukaya, 2015; Lee et al., 2018; Omidbakhshfard et al., 2015; Rodriguez et al., 2016). In addition, GRF transcripts are regulated by microRNA miR396 (Casadevall et al., 2013; Omidbakhshfard et al., 2015).

Here, we identified the GRF gene family in celery, coriander, carrot, lettuce, and grape. The fold change of GRF gene family was less than 0.5 times in celery compared with lettuce, carrot and coriander (Fig. 5). However, the GRF gene family was

significantly contraction in grape, and only accounted for ~1% of all GRF genes in these 7 species (Supplementary Fig. 29). The number of GRF genes was 3, 5, 105, 14, 55, 98, and 21 in grape, Arabidopsis, lettuce, ginseng, carrot, coriander, and celery, respectively. In addition, the expression analyses showed that most (90.47%) GRF family genes were not expressed in the 3 tissues or 3 varieties (Supplementary Fig. 30).

FAR1 gene family

The far-red-impaired response (FAR1) gene has reduced responsiveness to continuous far-red light, but responds normally to other light wavelengths. The FAR1 gene encodes a novel nuclear protein specific to phytochrome A signaling, which consists of at least four genes in Arabidopsis(Hudson et al., 1999). FAR1 and FHY3 (far-red elongated hypocotyls 3) are two homologous proteins, which are essential for phytochrome A regulated far-red responses in Arabidopsis(Lin and Wang, 2004). In addition, they have crucial functions in plant growth and development. FAR1 and FHY3 and are the founding members of the FRS (FAR1-RELATED SEQUENCE) and FRF (FRS-RELATED FACTOR) families, which are conserved among land plants(Ma and Li, 2018).

Here, we identified the FAR1 gene family in celery, coriander, carrot, lettuce, and grape. The fold change of FAR1 gene family was over than 2 times in grape compared with celery (Fig. 4). The number of FAR1 genes was 41, 17, 6, 131, 5, 10, and 11 in grape, Arabidopsis, lettuce, ginseng, carrot, coriander, and celery, respectively. We found FAR1 gene family was significantly expansion in ginseng, and accounted for 59% of all FAR1 genes in these 7 species (Supplementary Fig. 29). However, the FAR1 gene family was significantly contraction in lettuce and carrot, only accounted for 3% and 2% of all FAR1 genes in these 7 species. In addition, we found that some genes showed high expression level in both 3 tissues and varieties, such as *Ag3G01584.1*, compared with other genes, although they belonged to the same gene family (Supplementary Fig. 30).

STAT gene family

STAT (Signal Transducer and Activator of Transcription) proteins are a family of latent cytoplasmic transcription factors, which are activated by cytokines and growth factors. The STAT translocate to the nucleus, bind to specific promoter elements of target genes and regulate their transcription (Heim, 2003). The STATs have been identified as a

part of a signaling pathway that initiates in the plasma membrane but quickly translocate to the cytoplasm and to the nucleus to regulate the target genes (Lee and Gao, 2005). The STAT signaling pathway is one of the seven common pathways that control cell fate decisions during animal development (Wang and Levy, 2012). STATs are known in many non-plant species, and act as intracellular intermediaries between extracellular ligands and activation of target genes (Richards et al., 2000).

Here, we identified the STAT gene family in these 7 species. The fold change of STAT gene family was less than 0.5 times in celery compared with Arabidopsis, lettuce, carrot, and coriander. The number of STAT genes was 1, 3, 3, 0, 3, 3, and 1 in grape, Arabidopsis, lettuce, ginseng, carrot, coriander, and celery, respectively (Supplementary Fig. 29).

7. References

Adey, A., Kitzman, J.O., Burton, J.N., Daza, R., Kumar, A., Christiansen, L., et al. (2014) In vitro, long-range sequence information for de novo genome assembly via transposase contiguity. *Genome Res* **24**, 2041-2049.

Anders, S. and Huber, W. (2010) Differential expression analysis for sequence count data. *Genome Biol* **11**, R106.

Anders, S., Pyl, P.T. and Huber, W. (2015) HTSeq--a Python framework to work with high-throughput sequencing data. *Bioinformatics* **31**, 166-169.

Bairoch, A. (2005) From sequences to knowledge, the role of the Swiss-Prot component of UniProt. *Molecular & Cellular Proteomics* **4**, S2-S2.

Bairoch, A. and Apweiler, R. (2000) The SWISS-PROT protein sequence database and its supplement TrEMBL in 2000. *Nucleic Acids Research* **28**, 45-48.

Bao, W., Kojima, K.K. and Kohany, O. (2015) Repbase Update, a database of repetitive elements in eukaryotic genomes. *Mob DNA* **6**, 11.

Benson, G. (1999) Tandem repeats finder: a program to analyze DNA sequences. *Nucleic Acids Res* **27**, 573-580.

Birney, E., Clamp, M. and Durbin, R. (2004) GeneWise and Genomewise. *Genome Res* **14**, 988-995.

Burton, J.N., Adey, A., Patwardhan, R.P., Qiu, R., Kitzman, J.O. and Shendure, J. (2013) Chromosome-scale scaffolding of de novo genome assemblies based on chromatin interactions. *Nat Biotechnol* **31**, 1119-1125.

Camacho, C., Coulouris, G., Avagyan, V., Ma, N., Papadopoulos, J., Bealer, K. and Madden, T.L. (2009) BLAST+: architecture and applications. *BMC Bioinformatics* **10**, 421.

Casadevall, R., Rodriguez, R.E., Debernardi, J.M., Palatnik, J.F. and Casati, P. (2013) Repression of growth regulating factors by the microRNA396 inhibits cell proliferation by UV-B radiation in Arabidopsis leaves. *Plant Cell* **25**, 3570-3583.

Chan, P.P. and Lowe, T.M. (2019) tRNAscan-SE: Searching for tRNA Genes in Genomic Sequences. *Methods Mol Biol* **1962**, 1-14.

Chin, C.S., Alexander, D.H., Marks, P., Klammer, A.A., Drake, J., Heiner, C., et al. (2013) Nonhybrid, finished microbial genome assemblies from long-read SMRT sequencing data. *Nat Methods* **10**, 563-569.

Chin, C.S., Peluso, P., Sedlazeck, F.J., Nattestad, M., Concepcion, G.T., Clum, A., et al. (2016) Phased diploid genome assembly with single-molecule real-time sequencing. *Nat Methods* **13**, 1050-1054.

Edgar, R.C. and Myers, E.W. (2005) PILER: identification and classification of genomic repeats. *Bioinformatics* **21 Suppl 1**, i152-158.

Emms, D.M. and Kelly, S. (2019) OrthoFinder: phylogenetic orthology inference for comparative genomics. *Genome Biol* **20**, 238.

Etherington, G.J., Ramirez-Gonzalez, R.H. and MacLean, D. (2015) bio-samtools 2: a

package for analysis and visualization of sequence and alignment data with SAMtools in Ruby. *Bioinformatics* **31**, 2565-2567.

Gemayel, R., Cho, J., Boeynaems, S. and Verstrepen, K.J. (2012) Beyond Junk-Variable Tandem Repeats as Facilitators of Rapid Evolution of Regulatory and Coding Sequences. *Genes-Basel* **3**, 461-480.

Haas, B.J., Delcher, A.L., Mount, S.M., Wortman, J.R., Smith, R.K., Hannick, L.I., et al. (2003) Improving the Arabidopsis genome annotation using maximal transcript alignment assemblies. *Nucleic Acids Research* **31**, 5654-5666.

Haas, B.J., Salzberg, S.L., Zhu, W., Pertea, M., Allen, J.E., Orvis, J., White, O., Buell, C.R. and Wortman, J.R. (2008) Automated eukaryotic gene structure annotation using EVidenceModeler and the Program to Assemble Spliced Alignments. *Genome Biol* **9**, R7.

Heim, M.H. (2003) The STAT Protein Family. In: *Signal Transducers and Activators of Transcription (STATs): Activation and Biology* (Sehgal, P.B., Levy, D.E. and Hirano, T. eds), pp. 11-26. Dordrecht: Springer Netherlands.

Hudson, M., Ringli, C., Boylan, M.T. and Quail, P.H. (1999) The FAR1 locus encodes a novel nuclear protein specific to phytochrome A signaling. *Genes Dev* **13**, 2017-2027.

Jaillon, O., Aury, J.M., Noel, B., Policriti, A., Clepet, C., Casagrande, A., et al. (2007) The grapevine genome sequence suggests ancestral hexaploidization in major angiosperm phyla. *Nature* **449**, 463-467.

Jiao, Y., Leebens-Mack, J., Ayyampalayam, S., Bowers, J.E., McKain, M.R., McNeal, J., et al. (2012) A genome triplication associated with early diversification of the core eudicots. *Genome Biol* **13**, R3.

Jo, H. and Koh, G. (2015) Faster single-end alignment generation utilizing multi-thread for BWA. *Biomed Mater Eng* **26 Suppl 1**, S1791-1796.

Katoh, K. and Standley, D.M. (2013) MAFFT multiple sequence alignment software version 7: improvements in performance and usability. *Mol Biol Evol* **30**, 772-780.

Kent, W.J. (2002) BLAT -- The BLAST-Like Alignment Tool. *Genome Research* **4**, 656-664.

Kim, D., Langmead, B. and Salzberg, S.L. (2015) HISAT: a fast spliced aligner with low memory requirements. *Nat Methods* **12**, 357-360.

Kim, J.H. and Tsukaya, H. (2015) Regulation of plant growth and development by the GROWTH-REGULATING FACTOR and GRF-INTERACTING FACTOR duo. *J Exp Bot* **66**, 6093-6107.

Korf, I. (2004) Gene finding in novel genomes. *BMC Bioinformatics* **5**, 59.

Kumar, S., Stecher, G., Suleski, M. and Hedges, S.B. (2017) TimeTree: A Resource for Timelines, Timetrees, and Divergence Times. *Mol Biol Evol* **34**, 1812-1819.

Lee, S.J., Lee, B.H., Jung, J.H., Park, S.K., Song, J.T. and Kim, J.H. (2018)

GROWTH-REGULATING FACTOR and GRF-INTERACTING FACTOR Specify Meristematic Cells of Gynoecia and Anthers. *Plant Physiol* **176**, 717-729.

Lee, S.o. and Gao, A.C. (2005) STAT3 and Transactivation of Steroid Hormone Receptors. In: *Vitamins & Hormones* pp. 333-357. Academic Press.

Li, H., Handsaker, B., Wysoker, A., Fennell, T., Ruan, J., Homer, N., et al. (2009) The Sequence Alignment/Map format and SAMtools. *Bioinformatics* **25**, 2078-2079.

Lin, R. and Wang, H. (2004) Arabidopsis FHY3/FAR1 gene family and distinct roles of its members in light control of Arabidopsis development. *Plant Physiol* **136**, 4010-4022.

Ma, L. and Li, G. (2018) FAR1-RELATED SEQUENCE (FRS) and FRS-RELATED FACTOR (FRF) Family Proteins in Arabidopsis Growth and Development. *Front Plant Sci* **9**, 692.

Marcais, G. and Kingsford, C. (2011) A fast, lock-free approach for efficient parallel counting of occurrences of k-mers. *Bioinformatics* **27**, 764-770.

Mayer, C., Leese, F. and Tollrian, R. (2010) Genome-wide analysis of tandem repeats in Daphnia pulex--a comparative approach. *BMC Genomics* **11**, 277.

Melters, D.P., Bradnam, K.R., Young, H.A., Telis, N., May, M.R., Ruby, J.G., et al. (2013) Comparative analysis of tandem repeats from hundreds of species reveals unique insights into centromere evolution. *Genome Biol* **14**, R10.

Mulder, N.J. and Apweiler, R. (2008) The InterPro database and tools for protein domain analysis. *Curr Protoc Bioinformatics* **Chapter 2**, Unit 2 7.

Nawrocki, E.P. and Eddy, S.R. (2013) Infernal 1.1: 100-fold faster RNA homology searches. *Bioinformatics* **29**, 2933-2935.

Ogata, H., Goto, S., Sato, K., Fujibuchi, W., Bono, H. and Kanehisa, M. (1999) KEGG: Kyoto Encyclopedia of Genes and Genomes. *Nucleic Acids Research* **27**, 29-34.

Omidbakhshfard, M.A., Proost, S., Fujikura, U. and Mueller-Roeber, B. (2015) Growth-Regulating Factors (GRFs): A Small Transcription Factor Family with Important Functions in Plant Biology. *Mol Plant* **8**, 998-1010.

Parra, G., Bradnam, K. and Korf, I. (2007) CEGMA: a pipeline to accurately annotate core genes in eukaryotic genomes. *Bioinformatics* **23**, 1061-1067.

Paterson, A.H., Wendel, J.F., Gundlach, H., Guo, H., Jenkins, J., Jin, D., et al. (2012) Repeated polyploidization of Gossypium genomes and the evolution of spinnable cotton fibres. *Nature* **492**, 423-427.

Peska, V. and Garcia, S. (2020) Origin, Diversity, and Evolution of Telomere Sequences in Plants. *Front Plant Sci* **11**, 117.

Price, A.L., Jones, N.C. and Pevzner, P.A. (2005) De novo identification of repeat families in large genomes. *Bioinformatics* **21 Suppl 1**, i351-358.

Richards, D.E., Peng, J. and Harberd, N.P. (2000) Plant GRAS and metazoan STATs: one family? *Bioessays* **22**, 573-577.

Rodriguez, R.E., Ercoli, M.F., Debernardi, J.M. and Palatnik, J.F. (2016) Chapter 17 -

Growth-Regulating Factors, A Transcription Factor Family Regulating More than Just Plant Growth. In: *Plant Transcription Factors* (Gonzalez, D.H. ed) pp. 269-280. Boston: Academic Press.

Sambrook, J. and Russell, D.W. (2006) Purification of Nucleic Acids by Extraction with Phenol:Chloroform. *Cold Spring Harbor Protocols* **2006**, pdb.prot4455.

Seppey, M., Manni, M. and Zdobnov, E.M. (2019) BUSCO: Assessing Genome Assembly and Annotation Completeness. *Methods Mol Biol* **1962**, 227-245.

Somanathan, I. and Baysdorfer, C. (2018) A bioinformatics approach to identify telomere sequences. *Biotechniques* **65**, 20-25.

Stamatakis, A. (2014) RAxML version 8: a tool for phylogenetic analysis and post-analysis of large phylogenies. *Bioinformatics* **30**, 1312-1313.

Stanke, M. and Morgenstern, B. (2005) AUGUSTUS: a web server for gene prediction in eukaryotes that allows user-defined constraints. *Nucleic Acids Res* **33**, W465-467.

Tarailo-Graovac, M. and Chen, N. (2009) Using RepeatMasker to identify repetitive elements in genomic sequences. *Curr Protoc Bioinformatics* **Chapter 4**, Unit 4 10.

Trapnell, C., Williams, B.A., Pertea, G., Mortazavi, A., Kwan, G., van Baren, M.J., Salzberg, S.L., Wold, B.J. and Pachter, L. (2010) Transcript assembly and quantification by RNA-Seq reveals unannotated transcripts and isoform switching during cell differentiation. *Nat Biotechnol* **28**, 511-515.

Vekemans, X., Beauwens, T., Lemaire, M. and Roldan-Ruiz, I. (2002) Data from amplified fragment length polymorphism (AFLP) markers show indication of size homoplasy and of a relationship between degree of homoplasy and fragment size. *Mol Ecol* **11**, 139-151.

Walker, B.J., Abeel, T., Shea, T., Priest, M., Abouelliel, A., Sakthikumar, S., et al. (2014) Pilon: an integrated tool for comprehensive microbial variant detection and genome assembly improvement. *PLoS One* **9**, e112963.

Wang, J., Sun, P., Li, Y., Liu, Y., Yu, J., Ma, X., et al. (2017a) Hierarchically aligning 10 legume genomes establishes a family-level genomics platform. *Plant physiology* **174**, 284.

Wang, J., Sun, P., Li, Y., Liu, Y., Yu, J., Ma, X., et al. (2017b) Hierarchically Aligning 10 Legume Genomes Establishes a Family-Level Genomics Platform. *Plant Physiol* **174**, 284-300.

Wang, J., Yuan, J., Yu, J., Meng, F., Sun, P., Li, Y., et al. (2019) Recursive Paleohexaploidization Shaped the Durian Genome. *Plant Physiol* **179**, 209-219.

Wang, X., Guo, H., Wang, J., Lei, T., Liu, T., Wang, Z., et al. (2016a) Comparative genomic de-convolution of the cotton genome revealed a decaploid ancestor and widespread chromosomal fractionation. *New Phytologist* **209**, 1252-1263.

Wang, X., Guo, H., Wang, J., Lei, T., Liu, T., Wang, Z., et al. (2016b) Comparative genomic de-convolution of the cotton genome revealed a decaploid ancestor and

widespread chromosomal fractionation. *New Phytol* **209**, 1252-1263.

Wang, X., Shi, X., Hao, B., Ge, S. and Luo, J. (2005) Duplication and DNA segmental loss in the rice genome: implications for diploidization. *New Phytologist* **165**, 937-946.

Wang, X., Shi, X., Li, Z., Zhu, Q., Kong, L., Tang, W., Ge, S. and Luo, J. (2006) Statistical inference of chromosomal homology based on gene colinearity and applications to Arabidopsis and rice. *BMC bioinformatics* **7**, 447.

Wang, X., Wang, J., Jin, D., Guo, H., Lee, T.H., Liu, T. and Paterson, A.H. (2015) Genome Alignment Spanning Major Poaceae Lineages Reveals Heterogeneous Evolutionary Rates and Alters Inferred Dates for Key Evolutionary Events. *Molecular plant* **8**, 885-898.

Wang, X., Wang, Z., Guo, H., Zhang, L., Wang, L., Li, J., Jin, D. and Paterson, A.H. (2016c) Telomere-centric genome repatterning determines recurring chromosome number reductions during the evolution of eukaryotes. *New Phytol* **205**, 12.

Wang, Y. and Levy, D.E. (2012) Comparative evolutionary genomics of the STAT family of transcription factors. *JAKSTAT* **1**, 23-33.

Wang, Y., Tang, H., Debarry, J.D., Tan, X., Li, J., Wang, X., et al. (2012) MCScanX: a toolkit for detection and evolutionary analysis of gene synteny and collinearity. *Nucleic Acids Research* **40**, e49-e49.

Waterhouse, R.M., Seppey, M., Simao, F.A. and Zdobnov, E.M. (2019) Using BUSCO to Assess Insect Genomic Resources. *Methods Mol Biol* **1858**, 59-74.

Xu, Z. and Wang, H. (2007) LTR_FINDER: an efficient tool for the prediction of full-length LTR retrotransposons. *Nucleic Acids Res* **35**, W265-268.

Yang, Z. (2007) PAML 4: phylogenetic analysis by maximum likelihood. *Mol Biol Evol* **24**, 1586-1591.
